# Long-Term Effects of Interprofessional Biopsychosocial Rehabilitation for Adults with Chronic Non-Specific Low Back Pain: A Multicentre, Quasi-Experimental Study

**DOI:** 10.1371/journal.pone.0118609

**Published:** 2015-03-13

**Authors:** Jana Semrau, Christian Hentschke, Jana Buchmann, Karin Meng, Heiner Vogel, Hermann Faller, Hartmut Bork, Klaus Pfeifer

**Affiliations:** 1 Friedrich-Alexander-University Erlangen-Nürnberg, Institute of Sport Science and Sport, Erlangen, Germany; 2 University of Würzburg, Department of Medical Psychology, Medical Sociology, and Rehabilitation Sciences, Würzburg, Germany; 3 Reha-Zentrum am Sankt Josef-Stift, Sendenhorst, Germany; University of Glasgow, UNITED KINGDOM

## Abstract

**Background:**

Improvement of the long-term effectiveness of multidisciplinary ortho-paedic rehabilitation (MOR) in the management of chronic non-specific low back pain (CLBP) remains a central issue for health care in Germany. We developed an interprofessional and interdisciplinary, biopsychosocial rehabilitation concept named “PASTOR” to promote self-management in adults with CLBP and compared its effectiveness with the current model of MOR.

**Methods:**

A multicentre quasi-experimental study with three measurement time points was implemented. 680 adults aged 18 to 65 with CLBP were assed for eligibil-ity in three inpatient rehabilitation centres in Germany. At first the effects of the MOR, with a total extent of 48 hours (control group), were assessed. Thereafter, PASTOR was implemented and evaluated in the same centres (intervention group). It consisted of six interprofessional modules, which were provided on 12 days in fixed groups, with a total extent of 48 hours. Participants were assessed with self-report measures at baseline, discharge, and 12 months for functional ability (primary outcome) using the Hannover Functional Ability Questionnaire (FFbH-R) and vari-ous secondary outcomes (e.g. pain, health status, physical activity, pain coping, pain-related cognitions).

**Results:**

In total 536 participants were consecutively assigned to PASTOR (n=266) or MOR (n=270). At 12 months, complete data of 368 participants was available. The adjusted between-group difference in the FFbH-R at 12 months was 6.58 (95% CI 3.38 to 9.78) using complete data and 3.56 (95% CI 0.45 to 6.67) using available da-ta, corresponding to significant small-to-medium effect sizes of d=0.42 (p<0.001) and d=0.10 (p=0.025) in favour of PASTOR. Further improvements in secondary out-comes were also observed in favour of PASTOR.

**Conclusion:**

The interprofessional and interdisciplinary, biopsychosocial rehabilita-tion program PASTOR shows some improvements of the long-term effectiveness of inpatient rehabilitation in the management of adults with CLBP. Further insights into mechanisms of action of complex intervention programs are required.

**Trial Registration:**

ClinicalTrials.gov NCT02056951

## Background

Low back pain remains the leading musculoskeletal disease that is responsible for years lived with disability worldwide [1]. Based on the duration of an episode, low back pain that occurs for at least 12 weeks is defined as chronic [2]. The prevalence of disabling chronic low back pain has been estimated up to 11% [3,4]. Best estimates for Germany suggest that approximately 11% of the adult population is affected by disabling chronic back pain [5]. An economic estimate indicated that the average annual cost per person suffering from low back pain is € 1.322 in Germany [6]. Additionally, in cases where no specific pathological cause can be identified for low back pain the term “non-specific” has been established [2,7].

Musculoskeletal disorders, mainly chronic non-specific low back pain, are the most common indication for referral to a standard MOR in Germany [8]. MOR is a central component of the medical rehabilitation system in Germany and is carried out mainly as inpatient rehabilitation with an average duration of 22 days [9]. The treatment approach is based on the framework of the International Classification of Functioning, Disability and Health (ICF) [9,10] with a primary focus on improved functional health and return to work. MOR was not specifically developed for the treatment of CLBP, therefore important risk factors (e.g. catastrophizing, distress, fear avoidance beliefs) for the development of CLBP might be not explicitly addressed [11–13]. Furthermore, to date there are only a few controlled trials investigating the long-term effects of standard inpatient MOR in adults with CLBP in Germany [14–17], and its` long-term effectiveness on important outcome parameters (i.e. pain and function) has not yet been established [18].

Current international guidelines and reviews [2,7,19,20] recommend intensive multidisciplinary biopsychosocial rehabilitation (MBR) with a functional restoration approach in cases of disabling CLBP. This treatment approach is explicitly based on the assumption that the maintenance of CLBP and associated disability is determined by the interaction of biological, psychological and social factors [21]. Therefore, the overall objective is to restore physical, psychological, social and occupational functioning [22,23]. Previous evidence supported short term effectiveness of MBR in improving pain intensity and disability [19].

A recently published Cochrane Review [20] reported moderate to low quality evidence that MBR is more effective than usual care and physical treatment in reducing pain and disability at long-term although the reported effect sizes are small. How MBR programs should be designed (e.g. content and optimal sequence of contents, methods, intensity, therapist-patient relationships) to further improve outcomes at long-term and which patient groups benefit most is not entirely clear. Studies that compared two different active MBR programs might help to develop our understanding about relevant key factors of such programs to improve long-term effectiveness. Most of the available studies which compared two MBR programs [14–16,24–33] were provided in an outpatient setting or a combination of outpatient and inpatient setting with a longer duration or focused only on one or two components of the MBR program or did not use cognitive-behavioural theories and health behaviour change theories. It is not clear to what extent the results of international studies with different health care systems can be generalised to a 3-week inpatient rehabilitation setting to improve the long-term effectiveness.

Based on the available evidence from systematic reviews regarding effective active treatment approaches in the management of CLBP until 2006 [22,34–38], as well as the consensus of an interprofessional expert panel, best practice recommendations for objectives, treatment principles, and components were developed for the German rehabilitation setting [39]. These best practice recommendations lead to the multiprofessional development of an interprofessional and interdisciplinary rehabilitation program named „PASTOR”, with a biopsychosocial approach for inpatient rehabilitation of individuals with CLBP. In summary, the novel program PASTOR considered a) cognitive-behavioural models such as the fear-avoidance model [12,40] and the avoidance-endurance model [41] b) theories of health behaviour change such as the health action process approach [42,43] as well as c) quality criteria for patient education programs such as standardised manual, fixed groups, interactive education format and multiprofessional program development [44]. The overall objective of PASTOR was to promote active self-management of CLBP. The main components of PASTOR were biopsychosocial health education, behavioural exercise therapy, cognitive-behavioural psychological therapy, as well as workplace related information.

The major aim of the present study was to analyse the long-term effectiveness of PASTOR for participants with CLBP compared to the standard inpatient MOR in Germany. We hypothesized that in adults with CLBP the rehabilitation program PASTOR would result in a significantly higher increase in functional ability 12 months after completion of the program in comparison to the standard inpatient MOR. We further hypothesized that PASTOR would lead to significantly larger improvements regarding pain-related cognitions, pain coping strategies, physical activity, health-related quality of life, and back pain episodes compared to the standard inpatient MOR.

## Methods

### Study design and process

The protocols for this trial and supporting TREND checklist are available as supporting information; see S1 Checklist, S1 Protocol and S2 Protocol.

A multicentre prospective quasi-experimental control group design was implemented with three time points of measurement; at the beginning and end of rehabilitation, as well as 12-month follow-up. The study took place in three inpatient rehabilitation centres in Germany between January 2008 and December 2010. Originally four rehabilitation centres were planned to be involved. One of them dropped out at the beginning of the implementation phase due to internal organisational issues.

A randomized design was not feasible because both rehabilitation programs could not be carried out simultaneously in the rehabilitation centres due to staff and spatial requirements. Therefore, we used a quasi-experimental design, controlling for potential confounders. Participants were assigned to the study group consecutively at the time of admission into the participating rehabilitation centres („cohort design with cyclical turnover“[45]).

After a preparation phase (January 2008–June 2008), the control phase (July 2008–December 2008) commenced. During the control phase data was gathered at the beginning (t1) and the end (t2) of MOR (control group). Participant recruitment in the control phase started from July 2008 until December 2008, and the 12-month follow-up lasted from July 2009 until December 2009. Subsequently, PASTOR (intervention group) was implemented in all three rehabilitation centres between January 2009 and May 2009 during the implementation phase. During the intervention phase (June 2009–December 2009) data was gathered at the beginning (t1) and the end (t2) of PASTOR. Participant recruitment in the intervention phase started from June 2009 until December 2009, and the 12-month follow-up lasted from June 2010 until December 2010.

### Participants

**Inclusion and exclusion criteria.** In each rehabilitation centre, a rehabilitation physician invited eligible persons with CLBP to participate in the study. These persons, originating from different regions in Germany, had applied for an MOR referring to the regulation of the German pension insurance. Each application was accompanied by a general practitioner’s report on diagnostic findings (“assignment diagnosis”). Based on the European guidelines´ definition of chronic non-specific low back pain (www.backpaineurope.org), subjects were regarded as eligible if they had pain that persisted for at least three months, that was localized below the costal margin and above the inferior gluteal folds without referred leg pain, and which was not caused by a specific known pathology [2]. Based on these criteria, persons with the following assignment diagnoses (ICD-10, German version) from their general practitioners were included in the study: M51.2-M51.9, M53.8, M53.9 and M54.4-M54.9.

Persons with at least one of the following exclusion criteria were not recruited: age below 18 years or over 65 years, specific underlying diagnosis of back pain (e. g. radicular symptoms, myelopathy, formerly performed spine surgeries, inflammatory deformations of the spine), considerably reduced health status (e.g. comorbidities such as severe heart disease), considerably reduced sight and hearing, severe psychiatric condition as secondary diagnosis, inability to speak German, current application for early retirement or invalidity pension.

**Recruitment procedure.** The assignment of participants to the study groups took place at the time of admission into the participating rehabilitation centres. During the control phase participants were assigned to the control group, and during the intervention phase participants were assigned to the intervention group (see above). Potential participants who met the inclusion criteria were identified and informed about the study by their rehabilitation physician during the medical interview at the beginning of the inpatient rehabilitation period. Participants willing to participate were asked to give their signed informed consent on the next day. After the participants’ signed consent form (informed consent) was submitted, program enrolment took place. The distribution and collection of coded questionnaires at the beginning and end of the inpatient rehabilitation period was adjusted to each of the centres internal structures and admission processes.

The subjects received their 12-month follow-up questionnaires by postal letters through the rehabilitation centres. Additionally, postal reminders were sent after three weeks if the participants had not returned the questionnaire.

Every participating rehabilitation centre received a small reward (€ 50.00) for participants with complete data for all measurement time points (t1-t3). The purpose of this reward was to increase the support of the rehabilitation centres management in the recruitment process. The participants did not receive any reward.

### Ethical Aspects

The independent research ethics committee of the Medical Faculty at Friedrich-Alexander-University of Erlangen-Nürnberg (Re.-No. 3807) granted ethical approval for this study. The study was conducted according to the strict ethical and data protection demands of the German Pension Insurance (sponsor of this study) as well as according to the recommendations of the World Medical Association (Declaration of Helsinki: [46]).

Information for the participants and their agreement on study participation was included in the „informed consent“. According to the national data protection laws all personal data were treated as confidential and were used only for scientific purposes.

### Trial Registration

This trial has been registered at ClinicalTrials.gov (http://clinicaltrials.gov) under NCT02056951 after enrolment of participants started. This study was planned in 2006 and received a public funding from the German pension insurance in 2007. A trial registration was not required by the German pension insurance or the independent research ethics committee of the Medical Faculty at Friedrich-Alexander-University of Erlangen-Nürnberg. The public awareness, that the registration of all studies meeting the WHO definition of a clinical trial should be considered as a scientific, ethical and moral responsibility (http://www.who.int/ictrp/faq/en/), has increased in Germany during the last years. However, during the planning period of this trial (2006–2007) the registration of studies that evaluated the effectiveness of interventions with a primary educational focus within the German rehabilitation setting was not a necessary and well-established procedure in Germany. Since 2010 all interventional studies of our working group are registered in the trial registry ClinicalTrials.gov. Furthermore, the authors confirm that all ongoing and related trials for this intervention are registered.

### Interventions

**Control Group: multidisciplinary orthopaedic rehabilitation (MOR).** During the control phase, subjects received standard inpatient MOR in the participating rehabilitation centres, which lasted on average 23 days (SD ± 3.5 days). The central objectives of MOR were to improve functional health as described in the International Classification of Health (ICF) with the main focus on restoring and improving work ability [9,10]. A multiprofessional team consisting of the following qualified professions: physicians, psychologists, sport therapists, physiotherapists, occupational therapists, masseurs, social workers, dieticians and nurses delivered the MOR program. These professionals carried out various interventions from the physical and psychological dimensions as described by Guzman et al. [22]. This included health education lectures with information about health and health behaviour, exercise therapy and back school (predominately with a traditional biomedical approach), physical treatments (e.g. massage), psychological interventions in groups and individual counselling when necessary, and rehabilitation/social counselling. At the beginning of the rehabilitation period a differential diagnostic procedure by the attending physician took place, which formed the basis for assigning a detailed treatment plan to each subject. The treatment plan was provided, which guided the rehabilitation process and described the interventions distributed over the whole day during the rehabilitation stay. The total extent of therapy during MOR was 48 hours on average with three to five interventions on each treatment day. These 48 hours included five to seven hours for required individual treatments (e.g. additional PT session for a shoulder problem). The particular interventions within MOR were carried out mainly in open groups. Accordingly, group sessions of single interventions were open to new participants and the number of participants in each unit varied. The interventions of MOR were not specifically related to each other in terms of objectives, contents, methods or media. None of the rehabilitation centres had implemented a specific integrative interprofessional and interdisciplinary rehabilitation program before. More information about components of the standard inpatient rehabilitation is available as (S1 Intervention).

**Intervention group.** PASTOR was matched to the MOR in the control group with respect to the admission and assignment procedures, and the total duration with an average of 22 days (SD ± 2.3 days).

**Overall objectives of PASTOR.** The overall objective of PASTOR was the development of active self-management of CLBP through biopsychosocial patient education about low back pain, the introduction of physical activity with an emphasis on promoting positive experiences with exercises and the long-term maintenance of physical activity as well as to promote coping strategies when dealing with CLBP.

**Modules of PASTOR.** PASTOR consisted of six interprofessional therapy modules with sessions of 30 to 90 minutes in duration. In total, PASTOR comprised an extent of 48 hours on average. In each rehabilitation centre, the sessions of the six modules were distributed over 12 days during the inpatient rehabilitation period, which lasted on average 22 days (SD ±2.3 days). They took place in fixed groups (eight to twelve patients) and each module was led by a specific member of the rehabilitation team. Table 1 constitutes an overview of central themes and contents of the modules of PASTOR. Additionally, there were regular interprofessional team meetings once a week. Physical interventions (e.g. massages) were limited to two sessions per week per person. S2 Intervention and S3 Intervention show the distribution of each module over the 12 days.

**Table 1:** Description of PASTOR.

**Module: Education about low back pain (ELBP)**
**Sessions**: 5; **Duration**: 30–60min; **Profession**: physician
**Contents**:
Introduction to the program, introduction of the rehabilitation team, information about the specific goals and expectations for the rehabilitation stay, education about prevalence, course, and risk factors for the development of acute low back pain (ELBP1, 60minutes)
Education about characteristics of low back pain, including; the lack of pathomorphological causes, possibilities and limitations of diagnostic procedures, risk factors for chronic pain development, consequences of pain, and pain memory (ELBP 2, 45minutes)
Passive and active therapy options to deal with low back pain; the spine as a fascinating and strong system (ELBP 3, 45minutes)
Reflection on patient experiences during the rehabilitation program, counselling regarding recurring pain episodes ("flare ups“), and Red Flags (ELBP 4, 30minutes) final discussion and information about aftercare (ELBP 5, 30minutes)
**Module: Behavioural exercise therapy 1 (BET 1)**
**Sessions**: 12; **Duration**: 90min; **Profession**: physical therapist
**Contents**:
Repetition of key messages of ELBP sessions (BET 1, session 1 to 12)
Active play to get to know each other as well as encouraging positive exercise experiences (BET 1, session 1 to 12)
Education about positive health effects of physical activity on the body, mood and well-being, Recommendations for health-enhancing physical activities (BET1, session 4 to 5; session 9)
Education about the effects of physical activity on the relationship between pain and mood, “physical activity as active pain coping strategy” (BET1, session 6 to 8)
Introduction of walking and functional callisthenics and developing control strategies for self-directed execution (e.g. objective vs. subjective methods to measure physical activity intensity level, training documentation, exercise planning) (BET1, session 1 to 4)
Practicing lumbar stabilization strategies, activation of deep and global trunk muscles during everyday life and work related physical activities (BET1, session 6 to 8)
Action planning and coping planning during the rehabilitation stay (BET1, session 5 to 6) and for home (BET1, session 10 to 11) based on the previous experiences during the rehabilitation stay (see reflection in BET1, session 9)
Reflection on the practice of individually planned health-enhancing physical activities and sport activities during the rehabilitation stay (BET1, session 9, see BET2)
Discussion of aftercare services (BET1, session 12)
More information about a similar BET program, which differs only in duration, is available elsewhere [47]
**Module: Behavioural exercise therapy 2 (BET 2)**
**Sessions**: 11; **Duration**: 45–60min; **Profession**: physical therapist
**Contents**:
Consolidation of movement and regulation competencies for self-directed health-enhancing physical activities/ exercises (e.g. Walking, Nordic-Walking, strength training, aqua-jogging) under therapist supervision (BET2 session 1 to 5)
Individual selection of one to two health-enhancing physical activities/ exercises and self-directed performance of the chosen physical activities or exercises during the rehabilitation period (BET2, session 6 to 11)
**Module: Coping with pain (CWP)**
**Sessions**: 6, **Duration**: 60min, **Profession**: psychologist
**Contents**:
Pain perception and pain management, control of attention (CWP1)
Mutual influence of thoughts, feelings/emotions, and body reactions on pain (pain circle); maladaptive and adaptive thoughts when dealing with pain (CWP2)
Health-enhancing effects of relaxation and physical activity on pain cycle, the perception of pleasure and enjoyment (CWP3)
Fear avoidance behaviour and endurance behaviour, dealing with light and intense pain (CWP4)
Stress, work satisfaction and low back pain (CWP5)
Dealing with „flare ups“or recurring pain episodes, planning individual coping strategies for everyday life (CWP6)
**Module: Relaxation (R)**
**Sessions**: 11; **Duration**: 30–45min; **Profession**: psychologist
**Contents**:
Education about the mode of action and effects of progressive muscle relaxation as well as self-directed practicing and documentation (R1-R4)
Self-directed practicing of relaxation (R4-R11)
**Module: Workplace related information (WRI)**
**Sessions**: 2; **Duration**: 60min; **Profession**: physician or social worker
**Contents**:
Rehabilitation and retirement, social medical concepts, assessment of performance (WRI 1)
The Disabilities Act (SGB IX), Participation benefits and possibilities of vocational rehabilitation (WRI 2)

In summary, the differences between PASTOR and the standard inpatient MOR in Germany are characterized by, a) an integrative combination of profession related modules within a comprehensive and consistent treatment approach [48], b) an interprofessional and collaborative teamwork [49] based on profession related modules, c) the use of standardized methods, media and materials by all professions in the therapeutic team and d) a highly structured and detailed manual for the entire treatment process (see http://www.forschung-patientenorientierung.de/files/pastor_etm_2012_1.pdf (German version). Further key characteristics of PASTOR are described as (S2 Intervention).

### Outcomes assessment

Primary and secondary outcome measures were defined in accordance with international recommendations [50–52] by standardised, reliable and valid self-report questionnaires. In the current study we present the following main primary and secondary outcomes (see Table 2).

**Table 2:** Primary and secondary outcome measures.

Domain/Outcome measure	Questionnaire/Items	Assessment time point			Reference
**Primary Outcome**		t1	t2	t3	
**Physical functioning**	Hannover Functional Ability Questionnaire (FFbH-R)	x	x	x	[53]
**Secondary Outcomes**					
**Pain** Pain intensity	Numeric Rating Scale (NRS)	x	x	x	[54]
**Physical activity**	Freiburg Questionnaire of physical activity (FFkA)	x		x	[55]
**Health related quality of life**	Short-Form-12 (SF-12)	x	x	x	[56]
**Pain related cognitions, emotions and behaviour** Cognitive and behavioural pain coping strategies	Pain Management Questionnaire (FESV)	x	x	x	[57]
Fear-avoidance and endurance-related responses to pain	Avoidance-Endurance Questionnaire (AEQ)	x	x	x	[58]
**Others**Demographic characteristics	sex, age, self-reported work status, severely handicapped	x			[59]

t1 = baseline, t2 = end of rehabilitation, t3 = 12-month follow-up

As our primary outcome we used the level of functional ability 12 months after the end of inpatient rehabilitation measured by the Hannover Functional Ability Questionnaire (FFbH-R) [53]. Our secondary outcome measures referred to pain-related cognitions, emotions and behaviour, pain, physical activity and health-related quality of life (see Table 2). Further detailed information on the used instruments is available as (S1 Measurement Instruments).

### Sample Size and Power Calculation

Both study groups received an intensive and active treatment. Therefore, we expected small between-group differences at the end of rehabilitation (t2) and at 12 months (t3). Sample power (Software: G-Power 3.0) concerning the primary outcome was approximated for an analysis of covariance at t3 with a small effect size of Cohen’s d = 0.3, a 2-sided α = 0.05 and a test power of 1-β = 0.8. This approximation results in a sample size of at least 176 participants per group. Anticipating a dropout rate of 40%, it was necessary to include 588 participants (n = 294 per group).

### Blinding

Due to the quasi-experimental study design it was impossible to blind the staff of the participating rehabilitation centres because both programs in the control and intervention phase were performed in the same rehabilitation centres and were administered by the same staff.

Participants of the study were masked regarding the study group. They were informed through the rehabilitation staff and in the “informed consent” that the effectiveness of two rehabilitation programs was compared and that both programs met current scientific standards and were appropriate to improve health status. During the study period, participants were not informed as to whether they were a member of the control group or intervention group.

Furthermore, based on the quasi-experimental design contamination effects (e.g. exchange about intervention contents) between both study groups were prevented by finishing the control phase before starting the implementation of the new program PASTOR and intervention phase.

The researchers who performed the statistical data analyses were not blinded regarding the study groups.

## Statistical Methods

Baseline differences between both study groups were analysed by use of two-sample t-tests for parametric data and the chi-square test for nominal data. The same statistical procedures were used to compare differences at baseline and at the end of rehabilitation in demographic and clinical characteristics between participants with available data at 12 months and those lost to follow-up at 12 months.

To assess the long term treatment effect of PASTOR against MOR we used analysis of covariance (ANCOVA) of functional ability measured with the FFbH-R at the 12-month follow-up, adjusted for baseline differences, as primary analysis. To distinguish direct rehabilitation effect from this overall effect we additionally analysed group differences at end of rehabilitation in a separate ANCOVA. The hypotheses were a priori defined as confirmatory. We tested them in a closed test procedure [60], to exclude an inflation of the α-error rate. The same ANCOVA tests were carried out for all secondary outcomes adjusting for baseline measures as well as for baseline score of the primary outcome (FFbH-R). All tests in secondary outcomes are regarded exploratory. The primary and secondary analyses were performed as per protocol analyses. For the primary outcome (FFbH-R) all cases with available data at all measurement time points were included in the primary analysis. For secondary outcomes all cases with missing primary outcome were excluded.

For all between group differences adjusted mean differences with 95% confidence intervals, the level of significance (p<0.05, 2-sided) and the effect size Cohen’s d [61] are reported.

For the description of longitudinal effects within both groups effect sizes (ES) are reported [62]. ES was calculated as mean change from baseline to 12-month follow-up divided by the pooled standard deviation of baseline and 12-month follow-up measurements weighted by the respective sample size.

As sensitivity analysis (not included in the original study protocol) a saturated 3x2-factorial linear mixed effects model (LMM) with three assessment occasions (t1; t2; t3) and two groups (control group and intervention group) as fixed effects was used. This analysis was performed according to the intention to treat strategy. Due to the nature of this type of field research with possibly high rates of missing values due to dropout and incompletely filled questionnaires, LMM would include as much data as available compared to the ANCOVA specified in the study protocol, which only includes complete cases. Additionally, the LMM was used to explore different trends of several outcomes during the course of time. Changes in outcomes over time are represented by the linear slope of time variable per study group. To compare the efficacy of intervention vs. control group their interaction effect is of main interest. Because of the substantial disparity in duration and quality of the inpatient rehabilitation period (22 days on average) and the follow-up period (12 months), separate slopes for these two periods are estimated by a model with fractional polynomial b-splines of degree l = 1 (linear), which is equivalent to a factorial dummy coding of measurement occasions. Accordingly the slopes of both groups can be compared in both periods and overall simultaneously whilst controlling for statistically significant differences at baseline. To allow for random individual variability within the hierarchical structure of measurements nested in individuals we considered the intercept and both slopes of the two periods as random effects, and assumed an unstructured covariance matrix.

Based upon the estimated change from baseline to one year follow-up the proportions of participants with improved, equal and worsened trajectories were calculated [63]. Following this method, participants were defined to have equal trajectories, if change ranges within half a standard deviation of the baseline measurement. Improvement and worsening were defined above or below this line, respectively.

All statistical analyses were done with SPSS version 22 (SPSS, Inc., Chicago, Illinois) and the R environment of statistical computing version 3.0.2 (R Foundation for Statistical Computing, Vienna, Austria).

## Results

### Participants’ characteristics

**Fig 1:**
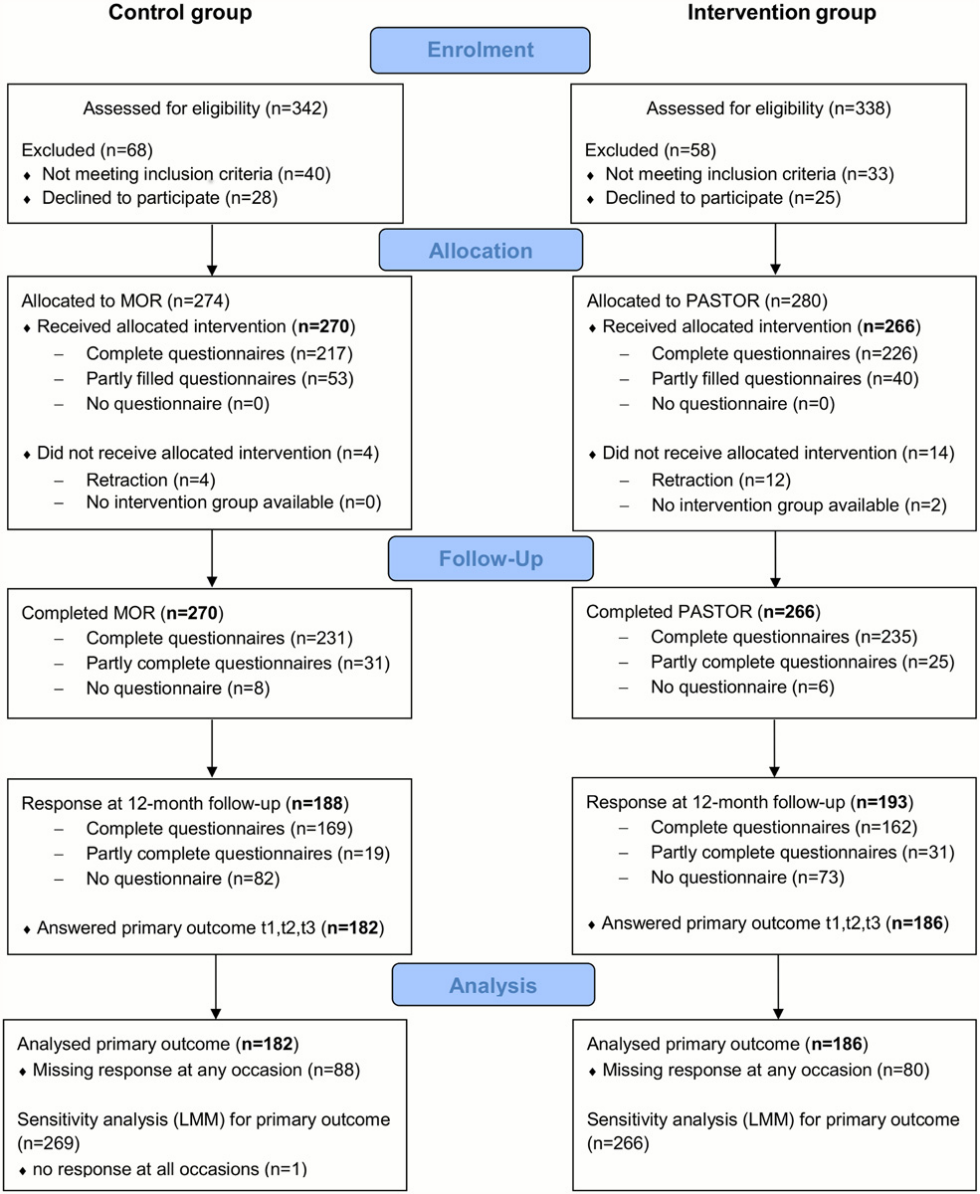
Flowchart.

Fig. 1 shows the flow of participants for the control group and intervention group throughout the study.

The mean age of participants was 49 years (SD = 8.2) and 275 of the 536 (51%) participants were female. Most demographic and clinical baseline characteristics were similar in both study groups (see Table 3). However, participants in the control group reported a lower physical component score (SF12) (p<0.001), as well as higher functional disability (FFbH-R) (p = 0.004).

**Table 3:** Baseline characteristics of participants consecutively assigned to treatment groups.

	Number Control group/ Intervention group	Control group	Intervention group
**Demographics**			
Age (years) M (SD)	270/266	49.3 (8.2)	48.9 (8.0)
Sex, female %	270/266	48.5	54.1
Currently in paid employment %	268/258	90.7	92.2
On sick leave at the beginning of rehabilitation %	267/263	16.9	13.7
Time off work for back pain last six month M (SD)	269/260	20.4 (37.3)	19.5 (39.7)
Severely handicapped pass %	270/265	5	3
**Physical functioning**			
Functional ability (FFbH-R) M (SD)	267/265	67.1 (19.0)	71.6 (17.3)
**General health**			
Physical component (SF 12) M (SD)	242/250	36.1 (8.4)	39.2 (8.7)
Mental component (SF 12) M (SD)	242/250	47.2 (10.8)	47.2 (11.6)
**Back Pain**			
Back pain intensity (NRS) M (SD)	267/266	5.9 (1.6)	5.7 (1.7)
**Physical activity**			
Total physical activity (hours per week) (FFkA) M (SD)	270/266	8.2 (6.0)	8.0 (5.5)

Data in the third and fourth column are either mean values (M) with standard deviation (SD) or percentages as specified in the first column; FFbH-R = Hannover Functional Ability Questionnaire, FFkA = Freiburg Questionnaire of physical activity, SF 12 = Short Form 12, NRS = Numeric Rating Scale

### Non-response and lost to follow-up

No differences were found between study participants and those who were not willing to participate in the study (non-responders) regarding sex and age.

There were few significant differences in demographic and clinical characteristics at baseline between participants with available data at 12 months and those lost to follow-up. For those lost to follow-up, a higher percentage reported that they were not currently employed (14.4%) compared to those with available data (6.2%). They also reported more sick leave days during the past six months due to back pain (27 days [SD 44.2]) compared to study participants with available data (17 days [SD 35.6]). A higher proportion of those lost to follow-up, approximately 7%, intended to claim disability pension compared to 3% of study participants with available data. Those lost to follow-up showed at baseline also a lower mental component score (SF12) (44.9 [SD 11.6] vs. 48.1 [SD 10.0]), slightly higher anxiety/ depression (AEQ) (2.4 [SD 1.3] vs. 2.1 [SD 1.2]) and lower positive mood (AEQ) (3.3 [SD 1.3] vs. 3.5 [SD 1.2]) compared to those with available data.

Regarding differences in clinical characteristics at the end of rehabilitation, data showed that those lost to follow-up had slightly lower physical component score (SF12) (42.0 [SD 8.7]) compared to participants with available data (43.7 [SD 8.8]), and slightly higher pain intensity (NRS) (4.7 [SD 2.0] vs. 4.1 [SD 1.9]). Those lost to follow-up reported at the end of rehabilitation also slightly lower action-oriented coping (FESV) (16.8 [SD 4.8] vs. 17.7 [SD 4.4]), cognitive restructuring (FESV) (15.7 [SD 4.6] vs. 16.9 [SD 4.2]), slightly higher anxiety/ depression (AEQ) (1.6 [SD 1.2] vs. 1.4 [SD 1.1]) and lower positive mood (3.9 [SD 1.2] vs. 4.2 [SD 1.1]) compared to those with available data. Study participants were significantly more likely to be satisfied (7.6 out of 10 [SD 1.8]) with the rehabilitation stay, than those lost to follow-up (6.9 out of 10 [SD 2.2]).

### Primary Outcome

In the primary analysis (ANCOVA) the adjusted between-group difference at the end of rehabilitation for functional ability (FFbH-R) was 4.53 (95% CI 1.91 to 7.16) with a significant small-to-medium effect size of d = 0.36 (p = 0.001). After the 12-month follow-up the adjusted between-group difference was 6.58 (95% CI 3.38 to 9.78), equating to a significant small-to-medium effect size of d = 0.42 (p<0.001) (see Table 4).

From baseline to the 12-month follow-up there was a small within-group mean change in functional ability (ES = 0.12) in the control group, and a moderate within-group mean change (ES = 0.41) in the intervention group.

**Table 4:** ANCOVA (primary analysis)—primary outcome functional ability (FFbH-R).

Outcome	Number Control group/ Intervention group	Control group M (SD)	Intervention group M (SD)	adjusted mean difference (95% CI) at the end and at 12 months	between-group effects (ANCOVA)	
					*P*	d
**Baseline (t1)**	182/186	66.30 (19.64)	72.32 (16.81)			
**End of rehabilitation (t2)**	182/186	70.94 (18.90)	79.26 (15.04)	4.53 (1.91 to 7.16)	**0.001**	**0.36**
**12-month follow-up (t3)**	182/186	68.86 (22.00)	79.26 (16.17)	6.58 (3.38 to 9.78)	**<0.001**	**0.42**

M = mean, SD = standard deviation; CI = confidence interval; ANCOVA = analysis of covariance; *P* = significance value; bold = significant between-group difference (*P*<0.05); d = Effect size Cohen’s d for the between group difference; FFbH-R = Hannover Functional Ability Questionnaire

The secondary analysis (LMM) showed no significant (p = 0.459) adjusted between-group difference from baseline to the end of rehabilitation (0.90; 95% CI-1.48 to 3.28). For the second phase, end of rehabilitation to the 12-month follow-up, the adjusted between-group difference was 2.67 (95% CI 0.01 to 5.32) with a small effect size of d = 0.09 (p = 0.049). For the complete phase from baseline to the 12-month follow-up the adjusted between-group difference was 3.56 (95% CI 0.45 to 6.68), equating to a significant small effect size of d = 0.10 (p = 0.025) (see Table 5).

From baseline to the 12-month follow-up there was a small within-group mean change in functional ability (ES = 0.08) in the control group, and a moderate within-group mean change (ES = 0.45) in the intervention group.

**Table 5:** LMM (secondary analysis)—primary outcome functional ability (FFbH-R).

Outcome	Number Control group/ Intervention group	Control group mean change	Intervention group mean change	adjusted mean difference (95% CI)	*P*	d
**t1—t2**		5.07	5.97	0.91 (-1.43 to 3.24)	0.447	0.03
**t2—t3**		-2.43	0.34	2.67 (0.01 to 5.32)	**0.049**	**0.09**
**total (t1—t3)**	269/266	2.69	6.25	3.56 (0.45 to 6.67)	**0.025**	**0.10**

LMM = linear mixed model; t1 = baseline, t2 = end of rehabilitation, t3 = 12-month follow-up; CI = confidence interval, P = significance value; bold = significant between-group difference (P<0.05); d = Effect size Cohen’s d for the between group difference; FFbH-R = Hannover Functional Ability Questionnaire

**Fig 2:**
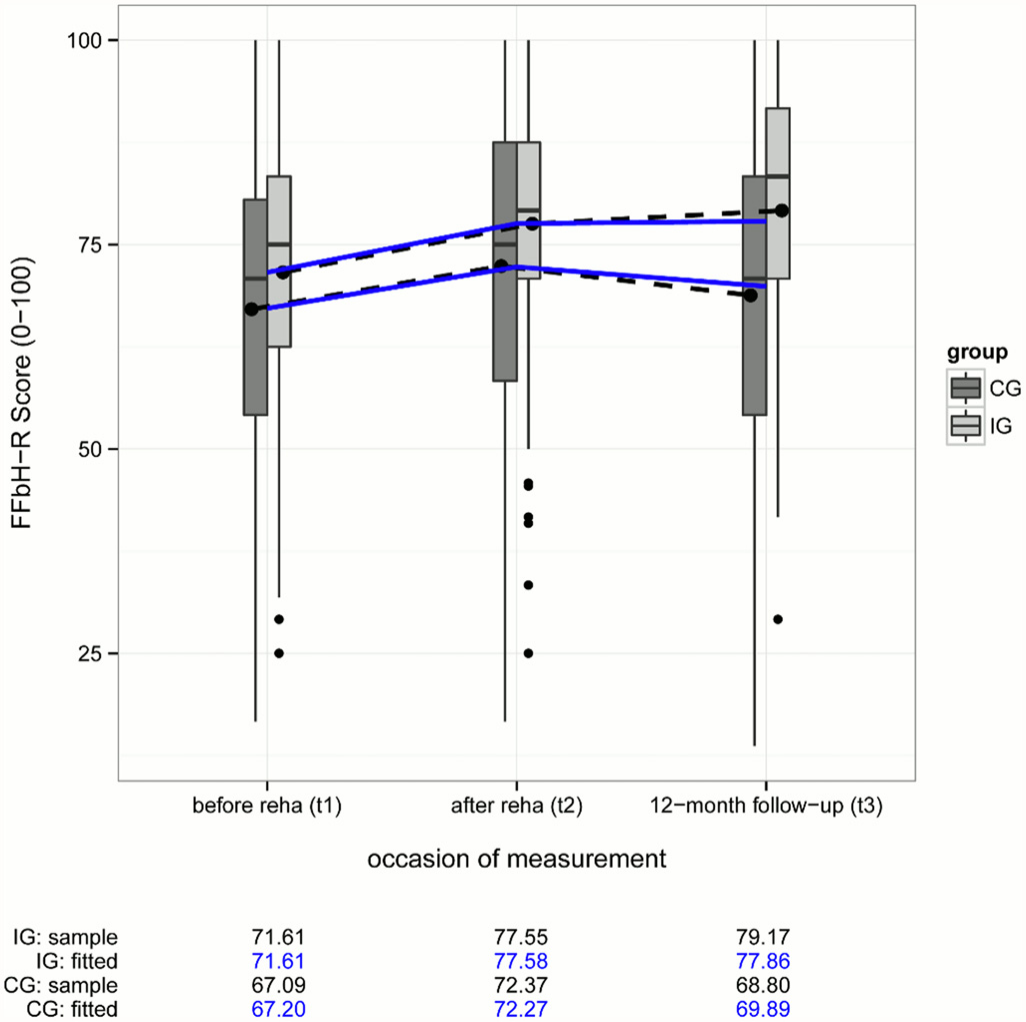
Boxplots and mean changes for FFbH-R in the CG and IG. CG = control group, IG = intervention group; FFbH-R = Hannover Functional Ability Questionnaire

In Fig. 2 boxplots for functional ability (FFbH-R) in the control and intervention group are displayed for each measurement occasion. Moreover marginal sample mean trajectories are represented by dashed lines with black points as mean values. Blue lines show the estimated trajectories from the linear mixed model analysis, which correct for attrition bias under a “missing at random” assumption. Means and estimated values are additionally indicated as numbers underneath.

Based on half a standard deviation from baseline to the 12-month follow-up 68 participants (intervention group) vs. 55 participants (control group) improved their functional ability, 103 participants (intervention group) vs. 92 participants (control group) did not change, and 21 (intervention group) vs. 39 (control group) worsened.

### Secondary Outcomes

In the ANCOVA there were significant adjusted between-group differences in favour of PASTOR for physical health status (SF 12), all cognitive and behavioural pain coping strategies (FESV) as well as the most fear-avoidance responses (AEQ) with the subscales help-/hopelessness, anxiety/depression, avoidance of social activities and physical activities at the end of rehabilitation, as well as at the 12-month follow-up (Tables 6–8). Regarding endurance responses (AEQ), there was a significant adjusted between-group difference in favour of PASTOR only for humor/distraction at the end of rehabilitation, and at the 12-month follow-up (Table 8).

**Table 6:** ANCOVA (primary analysis)—secondary outcomes SF 12, NRS, FFkA.

Outcome	Number Control group/ Intervention group	Control group M (SD)	Intervention group M (SD)	adjusted mean difference (95% CI) at the end and at 12 months	between-group effects (ANCOVA)	
					*P*	d
**Physical health status (SF 12)**						
Baseline (t1)	156/162	36.84 (8.09)	39.30 (8.71)			
End of rehabilitation (t2)	156/162	41.80 (9.07)	46.25 (7.38)	2.99 (1.44 to 4.54)	**<0.001**	**0.43**
12-month follow-up (t3)	156/162	40.27 (9.46)	44.22 (8.70)	2.61 (0.79 to 4.42)	**0.005**	**0.32**
**Mental health status (SF 12)**						
Baseline (t1)	156/162	47.78 (10.99)	48.70 (11.02)			
End of rehabilitation (t2)	156/162	54.61 (8.42)	55.17 (7.34)	0.06 (-1.50 to 1.63)	0.937	0.06
12-month follow-up (t3)	156/162	47.85 (11.58)	50.17 (10.37)	1.70 (-0.28 to 3.68)	0.093	0.19
**Low back pain intensity (NRS)**						
Baseline (t1)	179/184	5.78 (1.49)	5.63 (1.66)			
End of rehabilitation (t2)	179/184	4.30 (1.91)	3.94 (1.81)	-0.21 (-0.53 to 0.12)	0.213	0.13
12-month follow-up (t3)	179/184	4.79 (2.16)	4.22 (1.97)	-0.44 (-0.83 to-0.05)	**0.027**	**0.24**
**Sport activity (hours/week) (FFkA)**						
Baseline (t1)	182/186	1.19 (1.64)	1.44 (1.98)			
12-month follow-up (t3)	182/186	1.62 (2.04)	2.19 (2.19)	0.50 (0.10 to 0.90)	**0.015**	**0.26**
**Total physical activity (hours/week) (FFkA)**						
Baseline (t1)	182/186	8.52 (6.13)	8.12 (5.05)			
12-month follow-up (t3)	182/186	11.05 (6.30)	11.67 (6.01)	0.87 (-0.30 to 2.04)	0.146	0.16

M = mean, SD = standard deviation; CI = confidence interval; ANCOVA = analysis of covariance; *P* = significance value; bold = significant between-group difference (P<0.05); d = Effect size Cohen’s d for the between group difference; SF 12 = Short Form 12, NRS = Numeric Rating Scale, FFkA = Freiburg Questionnaire of physical activity

**Table 7:** ANCOVA (primary analysis)—secondary outcome FESV.

Outcome	Number Control group/ Intervention group	Control group M (SD)	Intervention group M (SD)	adjusted mean difference (95% CI) at the end and at 12 months	between-group effects (ANCOVA)	
					*P*	d
**Action-oriented coping**						
Baseline (t1)	181/183	14.91 (4.95)	14.65 (4.68)			
End of rehabilitation (t2)	181/183	16.61 (4.65)	18.80 (3.90)	2.35 (1.54 to 3.16)	**<0.001**	**0.60**
12-month follow-up (t3)	181/183	15.90 (4.85)	17.88 (4.01)	2.21 (1.37 to 3.04)	**<0.001**	**0.55**
**Subjective coping competence**						
Baseline (t1)	180/183	16.16 (4.37)	16.81 (4.28)			
End of rehabilitation (t2)	180/183	17.04 (3.95)	19.15 (3.00)	1.84 (1.20 to 2.49)	**<0.001**	**0.60**
12-month follow-up (t3)	180/183	16.76 (4.37)	18.44 (3.72)	1.44 (0.66 to 2.21)	**<0.001**	**0.40**
**Cognitive restructuring**						
Baseline (t1)	180/183	14.22 (4.81)	14.57 (4.39)			
End of rehabilitation (t2)	180/183	15.46 (4.30)	18.26 (3.51)	2.72 (1.98 to 3.47)	**<0.001**	**0.77**
12-month follow-up (t3)	180/183	15.22 (4.46)	16.95 (3.98)	1.67 (0.87 to 2.48)	**<0.001**	**0.43**
**Counter activities**						
Baseline (t1)	181/185	12.27 (4.37)	12.44 (4.47)			
End of rehabilitation (t2)	181/185	13.05 (4.23)	15.67 (3.84)	2.55 (1.83 to 3.27)	**<0.001**	**0.62**
12-month follow-up (t3)	181/185	12.16 (4.38)	14.89 (4.07)	2.73 (1.94 to 3.53)	**<0.001**	**0.71**
**Mental distraction**						
Baseline (t1)	181/184	10.55 (4.25)	11.29 (4.67)			
End of rehabilitation (t2)	181/184	11.71 (4.41)	14.55 (4.70)	2.55 (1.69 to 3.40)	**<0.001**	**0.62**
12-month follow-up (t3)	181/184	10.96 (4.56)	13.81 (4.72)	2.66 (1.78 to 3.54)	**<0.001**	**0.63**
**Relaxation**						
Baseline (t1)	181/184	11.92 (5.18)	11.97 (4.86)			
End of rehabilitation (t2)	181/184	14.31 (4.98)	16.22 (4.50)	1.91 (1.06 to 2.77)	**<0.001**	**0.46**
12-month follow-up (t3)	181/184	13.87 (5.00)	15.01 (4.77)	1.20 (0.34 to 2.06)	**0.006**	**0.29**

M = mean, SD = standard deviation; CI = confidence interval; ANCOVA = analysis of covariance; *P* = significance value; bold = significant between-group difference (P<0.05); d = Effect size Cohen’s d for the between group difference; FESV = Pain Management Questionnaire

**Table 8:** ANCOVA (primary analysis)—secondary outcome AEQ.

Outcome	Number Control group/ Intervention group	Control group M (SD)	Intervention group M (SD)	adjusted mean difference (95% CI) at the end and at 12 months	between-group effects (ANCOVA)	
					*P*	d
***Subscales fear-avoidance response pattern***						
**Anxiety/depression**						
Baseline (t1)	174/176	2.18 (1.17)	2.09 (1.15)			
End of rehabilitation (t2)	174/176	1.54 (1.10)	1.14 (0.95)	-0.34 (-0.53 to-0.15)	**<0.001**	**0.39**
12-month follow-up (t3)	174/176	2.02 (1.33)	1.65 (1.14)	-0.29 (-0.52 to-0. 07)	**0.009**	**0.29**
**Help-/hopelessness**						
Baseline (t1)	180/180	2.20 (1.25)	2.05 (1.06)			
End of rehabilitation (t2)	180/180	1.91 (1.21)	1.47 (0.99)	-0.34 (-0.51 to-0.17)	**<0.001**	**0.41**
12-month follow-up (t3)	180/180	2.04 (1.32)	1.58 (1.05)	-0.34 (-0.55 to-0.13)	**0.002**	**0.34**
**Catastrophizing**						
Baseline (t1)	181/182	0.91 (1.18)	0.76 (1.09)			
End of rehabilitation (t2)	181/182	0.81 (1.10)	0.55 (0.90)	-0.15 (-0.31 to 0.01)	0.062	0.20
12-month follow-up (t3)	181/182	0.91 (1.31)	0.60 (0.92)	-0.21 (-0.40 to-0.01)	**0.043**	**0.21**
**Avoidance of physical activities when dealing with heavy pain**						
Baseline (t1)	163/158	4.05 (1.13)	3.95 (1.12)			
End of rehabilitation (t2)	163/158	3.83 (1.17)	3.35 (1.16)	-0.40 (-0.61 to-0.18)	**<0.001**	**0.41**
12-month follow-up (t3)	163/158	3.83 (1.20)	3.36 (1.11)	-0.36 (-0.57 to-0.15)	**0.001**	**0.40**
**Avoidance of social activities when dealing with heavy pain**						
Baseline (t1)	160/158	2.80 (1.33)	2.56 (1.42)			
End of rehabilitation (t2)	160/158	2.68 (1.44)	2.06 (1.25)	-0.46 (-0.68 to-0.23)	**<0.001**	**0.45**
12-month follow-up (t3)	160/158	2.55 (1.45)	1.90 (1.29)	-0.48 (-0.73 to-0.22)	**<0.001**	**0.41**
***Subscales endurance response pattern***						
**Positive mood**						
Baseline (t1)	180/178	3.42 (1.20)	3.57 (1.22)			
End of rehabilitation (t2)	180/178	4.03 (1.09)	4.39 (1.11)	0.27 (0.07 to 0.47)	**0.009**	**0.28**
12-month follow-up (t3)	180/178	3.60 (1.25)	3.91 (1.23)	0.21 (-0.03 to 0.44)	0.088	0.18
**Thought suppression**						
Baseline (t1)	181/182	3.45 (1.52)	3.43 (1.44)			
End of rehabilitation (t2)	181/182	3.43 (1.43)	3.24 (1.46)	-0.18 (-0.43 to 0.07)	0.163	0.14
12-month follow-up (t3)	181/182	3.31 (1.50)	3.22 (1.43)	-0.03 (-0.29 to 0.24)	0.854	0.06
**Humor/distraction when dealing with heavy pain**						
Baseline (t1)	170/164	2.64 (1.07)	2.71 (1.09)			
End of rehabilitation (t2)	170/164	2.83 (1.11)	3.32 (1.10)	0.44 (0.23 to 0.64)	**<0.001**	**0.45**
12-month follow-up (t3)	170/164	2.83 (1.11)	3.27 (1.13)	0.39 (0.18 to 0.60)	**<0.001**	**0.40**
**Pain persistence behaviour when dealing with heavy pain**						
Baseline (t1)	160/157	3.34 (0.97)	3.37 (0.96)			
End of rehabilitation (t2)	160/157	3.37 (0.94)	3.45 (0.88)	0.03 (-0.13 to 0.19)	0.715	0.06
12-month follow-up (t3)	160/157	3.34 (1.08)	3.44 (0.94)	0.07 (-0.13 to 0.26)	0.490	0.09

M = mean, SD = standard deviation; CI = confidence interval; ANCOVA = analysis of covariance; *P* = significance value; bold = significant between-group difference (P<0.05); d = Effect size Cohen’s d for the between group difference; AEQ = Avoidance Endurance Questionnaire

For catastrophizing (AEQ) and pain intensity (NRS), a significant difference in favour of PASTOR was only observed at the 12-month follow-up. There was also a significant between-group differences in favour of PASTOR for the sport activity sub score (FFkA) at the 12-month follow-up (Table 6, Table 8).

For mental health score (SF 12), and total activity sub score (FFkA), as well as further endurance related responses to pain (AEQ) there were no differences between both groups at the end of rehabilitation, as well as at the 12-month follow-up (Table 6, Table 8).

The LMM for secondary outcomes showed that all cognitive and behavioural pain coping strategies (FESV) and most of the fear-avoidance responses (AEQ), as well as the sport activity sub score (FFkA) improved significantly more in the intervention group than in the control group. Significant adjusted mean differences in these variables occurred mainly during the period of rehabilitation and remained stable over time. For physical health status (SF 12), mental health score (SF 12), pain intensity (NRS), total activity sub score (FFkA), as well as for most of the endurance related responses to pain (AEQ) no significant adjusted mean differences were observed. Results of the LMM for secondary outcomes are described in the S1 Table. In both study groups, no adverse events or unintended effects were reported.

## Discussion

PASTOR resulted in a statistically significant improved functional ability, which was assessed using the Hannover Functional Ability Questionnaire (FFbH-R; primary outcome) at the end of rehabilitation as well as after 12 months, compared to the standard inpatient MOR. Both study groups received an active rehabilitation program with comparable treatment intensity. Therefore, the observed small between-group difference at 12 months is a notable amount.

Furthermore, the LMM showed that a significant small between-group difference in the change score of the primary outcome occurred during the longer phase from the end of rehabilitation to the 12-month follow-up. While the mean functional ability in MOR decreased, the mean functional ability in PASTOR was stabilized or slightly improved. However, the confidence intervals showed wide heterogeneity in the differences between both treatment groups.

Additionally, the significant between-group difference in the primary outcome at the end of rehabilitation in favour of PASTOR shown by the ANCOVA, could not be supported by the LMM. Different results are supposed to be primarily due to data availability for the two analyses at the end of rehabilitation. Data availability was considerably higher in LMM in comparison to a complete case ANCOVA, which also removes patients lost to follow-up at 12 months.

Overall, the results confirm our primary hypothesis that the rehabilitation program PASTOR increases functional ability more than standard inpatient MOR, and develops its effectiveness especially in the long-term.

With regards to the secondary outcomes, there were significant changes in favour of PASTOR in cognitive and behavioural pain coping strategies, as well as in most fear avoidance responses to pain. The LMM revealed that these significant changes occurred during the phase of rehabilitation, and remained stable over time. In contrast, endurance related responses did not change in both groups with the exception of humor/distraction. After 12 months a significant small between-group difference was also found in both analyses for sport activities favouring PASTOR.

The ANCOVA revealed further significant small between-group differences for pain intensity, physical health status, catastrophizing, and positive mood in favour of PASTOR, but this was not supported by the LMM.

### Possible mechanisms of action

**Long-term improvement of the primary outcome and secondary outcomes.** Improvement of self-reported functional ability may be achieved by physical reconditioning through exercise, although evidence is inconsistent [64,65]. There are either no or only weak relationships between changes in physical function measures and changes in function or pain in participants with CLBP after exercise therapy [64], and no evidence could be found that one type of exercise is more effective than another to improve clinical outcomes [66]. It has been discussed that psychosocial factors might act as an obstacle to disability reduction and recovery through different pathways [12,40,41]. The proportion of exercise therapy between both study groups did not differ. In contrast to MOR, there was a strong theory driven focus in all modules of PASTOR on influencing modifiable psychological and psychosocial risk factors for the development of CLBP. There is also evidence that persons with disabling CLBP probably show lower levels of physical activity [67]. Promoting a physical active lifestyle with appropriate levels of physical activity might therefore reduce disability in this study population. But, adherence to prescribed exercises and recommended levels of physical activity [68] is generally low and the use of strategies to improve adherence has been recommended [38,47,66,69–71]. In contrast to the exercise therapy in MOR, was the exercise therapy module in PASTOR explicitly based on a behavioural approach to promote a physically active lifestyle [71].

On the one hand, this together might have influenced the favourable short- and long-term improvements in all pain coping strategies, and might have also contributed to the development of a more adaptive response pattern to pain [41], as well as higher sport activity in the PASTOR group at 12 months. On the other hand, there was no considerable difference between both groups regarding pain intensity. This might be related to the only moderate levels of pain intensity in participants of both groups which was decreased in both groups in the long-term. Beyond that, direct pain relief was a major aim of MOR. In contrast, participants of PASTOR were informed throughout the program by all professions that the primary focus was improvement of self-management in dealing with CLBP rather than pain relief. Hence, pain intensity might be not an important barrier for disability reduction in this study population.

Overall, we suppose that explicit targeting of proximal modifiable psychosocial factors as well as determinants of behaviour change to promote a physically active lifestyle [7,38,64,69,70,72] has contributed to the more distal stabilization or slightly improved functional ability in the long-term with PASTOR, compared to a decrease at the same time in MOR. But, further evidence is still needed to prove causal relationships between different factors described in cognitive-behavioural models [12,40,41]. Evidence is also insufficient to understand how different maladaptive pain response patterns in individuals with CLBP can be modified best by different contents and treatment strategies and how this is related to improvements in function and pain [7,40,41,64,72,73].

**Key characteristics of PASTOR.** Overall, PASTOR was explicitly directed to improve individual self-management of CLBP through a) biopsychosocial education about low back pain, b) promoting a physically active lifestyle, and c) training of active coping strategies when dealing with CLBP. There was a strong focus of integrating treatments from different dimensions rather than adding them to each other. This is better described as interprofessional and interdisciplinary rehabilitation than multidisciplinary rehabilitation [49,74].

We hypothesized that such a program with these objectives would be able to improve the long-term self-reported functional ability. Therefore, we chose functional ability assessed with the FFbH-R as our primary outcome. Furthermore, functional ability was considered a global measure of improved self-management in dealing with CLBP. The degree of integration of the key characteristics of PASTOR, as shown in the S2 Intervention that covered the intervention description, might have contributed to the larger improvements of participant outcomes.

Treatment intensity, which includes about 100 hours of therapy, was also discussed as a decisive factor for the effectiveness of multidisciplinary treatments [22]. But, in our study treatment intensity of both study groups was well below this time volume with an average total extent of 48 hours. That treatment intensity is not a decisive factor for treatment effects is also supported by Kamper et al. [20].

In summary, with the quasi-experimental design of this trial and the complex intervention structure we are not able to identify which mechanisms influenced the outcome measures.

### Comparison with other relevant studies

There are only few studies available that compared two active inpatient rehabilitation programs. It is well known that there is much heterogeneity in methodological aspects e.g. differences in defined patient groups, study designs, content of treatments, comparison groups, study settings, outcome measures and instruments used [19,20,22]

In contrast to our results, a systematic review [19] provided moderate evidence favouring multidisciplinary biopsychosocial rehabilitation in terms of reduced pain in the short-term when compared to other active treatments, but did not find evidence for long-term effects on disability. Only one included study [26], rated with a moderate risk of bias, reported a decrease in pain and disability index at 12 months in favour of MBR compared to standard multidisciplinary rehabilitation.

A randomized controlled study with a heterogeneous patient population [15] and a controlled study [14] compared MBR with MOR in the German rehabilitation setting. While Mangels et al. [15] found no difference on a pain disability index measure in the long-term, Dibbelt et al. [14] reported significant effects on function and pain after ten months in favour of the multidisciplinary biopsychosocial rehabilitation. Another controlled study in Switzerland showed a significant slight improvement in function in favour of standard multidisciplinary rehabilitation compared to an interdisciplinary pain management program at six months and no difference in pain at mid-term [24]. In these studies [14,15,24,26] the treatments were similar in duration, approximately three weeks, and were carried out in an inpatient setting.

In summary, evidence for long-term effects on function with multidisciplinary biopsychosocial rehabilitation is inconsistent and none of these studies reported a small-to-medium between-group difference in function between two active inpatient rehabilitation treatments at 12 months as noted in our trial. The adjusted between-group difference in our trial on the FFbH-R was below 10%, which is generally in line with effect sizes that could be achieved in CLBP research [19,20]. In contrast to the comparisons reported by Kamper et al. [20] and van Middelkoop et al. [19] we compared two active inpatient rehabilitation programs. However, it is difficult to draw a final conclusion about the clinical relevance of our noted long-term between-group difference in function in favour of PASTOR as long as there is no consensus about the required magnitude of differences for clinical relevance between two active treatment groups [75].

### Comparison of long-term within-group changes of function

We observed only a small within-group mean change in functional ability in the long-term in our control group, which is similar to those reported in other trials [14,18]. For the intervention group a moderate within-group mean change in functional ability at 12 months could be established which is similar to the moderate effect on function after ten months for the intervention group in the trial of Dibbelt et al. [14]. To date there is no consensus about a minimal clinical important change to compare our primary outcome, which was assessed with the FFbH-R [63,76]. There are several methods to define a minimal clinical important change [76]. One option within anchor or distribution based methods [77], respectively, is to calculate different types of changes based on half a standard deviation (trajectories) [63]. Using this approach [63,78], the proportion of participants that reported a clinically important change in function was somewhat higher in the intervention group (PASTOR 35%; MOR 30%). Furthermore, the proportion of participants who reported a decline in function was noticeably higher in the control group (PASTOR 11%; MOR 21%). The proportion of participants that reported no clinically important change in function was similar in both study groups (PASTOR 54%; MOR 49%), which might be related to the only moderate functional limitations of the study population. Although PASTOR was developed for the rehabilitation setting, it could be argued that PASTOR in comparison to MOR worked more as a prevention strategy that reduces the risk of developing higher disability in the long run in this moderately impaired study population. Another finding is that function was stabilized and slightly improved after completion of PASTOR, especially during the longer phase. Thus far, none of the other studies [14,15,24,26] reported this development for function or any other outcome measure over time.

### Strengths and limitations

The strengths of our study include a standardised, detailed, manualized, theory-based and evidence-based interprofessional and interdisciplinary, biopsychosocial rehabilitation program, a high treatment integrity according to the study protocol supported by the detailed manualized procedure and intensive training of health professionals, delivery of the interventions by the same professionals in both study groups, no contamination between both study groups due to the study design, the use of LMM as sensitivity analyses as well as to explore the changes of outcome measures in different phases within the rehabilitation process, an acceptable recruitment rate, high response rate at the end of rehabilitation and an acceptable response rate after 12 months, as well as public and independent funding of the study.

**Study population and methodological aspects.** There was an approximately 9% lower than expected enrolment of study participants that might be a result of the lower than expected proportion of participant which fulfil the inclusion criteria or the dropout of one rehabilitation centre.

For our primary data analysis, we used the ANCOVA as it was defined in the study protocol. We further considered only complete cases, which is a very traditional approach with several disadvantages including an underestimation or overestimation of true effects depending on the patterns of missing values. We therefore used the LMM as a sensitivity analysis, where at least all available information was included. Most of the results of ANCOVA were confirmed by the LMM. Differences in some outcome measures between both statistical analyses might have been caused by the different numbers of analysed participants. The different results between the ANCOVA and the LMM regarding the primary outcome show that the ANCOVA overestimated the effects at the end of rehabilitation, as well as at the 12-month follow-up. This implies that participants with complete data were different from participants lost at follow-up. Hence, we cannot exclude that reported effects are prone to selection bias. The LMM analysis is more reliable, because it adjusts for missing data that could be explained by previous measurement occasions of the according outcome. Thus, we can not exclude that found effects are prone to selection bias due to informative missing data. However, a comparison between participants with complete data and those lost at follow-up regarding the primary outcome showed no significant differences at baseline or at the end of rehabilitation.

It might also be, that those participants who were lost at follow-up had improved or worsened secondary outcomes (informative missing data). In our study approximately 30% of participants were lost to follow-up at 12 months, which is generally comparable to other studies in the German rehabilitation setting [14,16,79]. Those lost to follow-up showed slightly worse results in a few socio-demographic and clinical outcomes at the end of rehabilitation. Based on these minor differences, as well as on the robustness of the used statistical procedures, we assume that the LMM analyses of the data provide a good estimate of the true effects.

Additionally, we cannot exclude that the analyses of a variety of secondary outcome measures might have increased the risk of significant effects by chance. Therefore, secondary outcome should be viewed as exploratory.

**Study design.** A limitation of our study might be that we used a quasi-experimental design instead of a randomized controlled design (RCT), because the simultaneous implementation of both complex rehabilitation programs in the participating rehabilitation centres was not possible due to organisational requirements (e.g. human resources, spatial resources). Another disadvantage of an RCT with simultaneous implementation of both study groups in the rehabilitation centres would have been a possible mixing of contents between the two treatment conditions. It might be that effects found in controlled quasi-experimental studies are overestimated due to a selection bias (difference between study groups caused by recruitment procedures). However, Furlan et al. [80] showed that controlled studies in contrast to randomized controlled studies rather underestimated the effects or estimated it equally, when comparing surgery and conservative treatment in the management of low back pain.

**Blinding of therapists and therapist-patient relationship.** Further limitations of our study include: no blinding of the health professionals in the rehabilitation centres because all professionals involved in the study carried out both treatments and were intensively trained for the PASTOR program; no blinding of participants, but they were informed that the effectiveness of two rehabilitation programs was compared and that both programs met current scientific standards and were appropriate to improve health status. We also did not control for therapist effects or therapist-patient relationship [81,82]. There was no variability of therapists between both treatments, because the same health professionals delivered both treatments. However, it might be that the intensive training procedure increased the motivation of the health professionals and improved their skills in delivering an intensive interprofessional and interdisciplinary, biopsychosocial treatment which might have positively influenced outcomes of participants [81–85].

### Clinical and research implications

PASTOR was implemented “top down” in three participating inpatient rehabilitation centres over a period of three to five months. The effort associated with the implementation of the program with regard to the establishment of fixed groups, planning of human and spatial resources, and the interprofessional training of the rehabilitation team is affordable. Since the project completion, PASTOR was continued in a shorter version in one rehabilitation centre. Another rehabilitation centre in which the usual care approach was pursued after the end of the study period has planned to reintroduce PASTOR. Single elements of PASTOR and part of the media and materials are still used in all rehabilitation centres even after the project ended. As inhibiting factors for a sustained implementation of PASTOR the rehabilitation teams mentioned temporal, spatial (rooms for fixed groups) and personnel barriers (number of psychologists). For the sustained implementation of complex rehabilitation programs such as PASTOR it seems to be useful to identify barriers and promoting factors within each rehabilitation centre, as well as to develop a “bottom up” implementation strategy in each rehabilitation centre with participation of stakeholders from the management of the rehabilitation centre, payers, and researchers. By using such a “bottom-up” approach, the dropout of one of the rehabilitation centres due to personnel barriers in our study may have been avoided. The current research is heterogeneous in terms of how effective programs can be sustainably implemented in health care settings and which factors influence sustainability [86]. Therefore, future research should focus on factors that promote or hinder the sustainable implementation of rehabilitation programs in health care settings, as well as how these factors interact and how they can be modified best.

As a limitation in our study we did not assess costs of both interventions. Cost-effectiveness analyses are urgently needed in order to justify the implementation of newly developed programs in routine care. Available routine data for the year 2013 show that in Germany a single rehabilitation treatment for physical diseases with duration of three weeks costs 2685€ on average [87]. As both interventions in our study require similar resources we assume that the costs for PASTOR are comparable to the costs of MOR. In PASTOR face-to-face times between one therapist and a single patient are reduced in favour of group interventions. This might further decrease the costs of PASTOR compared to MOR. The costs of implementation of PASTOR should also be taken into account e.g. expenditure of time for the preparation, implementation and reworking of train-the-trainer workshops, personnel costs, travel costs and costs for materials (e.g. Flipcharts, Cards). We did not assess those costs systematically, but they can estimated a 4000€ to 6000€ depending upon the conditions in particular rehabilitation centres. Furthermore benefits in terms of reduced direct (e.g. medications, health care consumption) and indirect (e.g. days of sick leave, early retirement) costs should be balanced against program costs. Those aspects of return-on-investment should be a focus in further studies.

We were able to show a small treatment effect in the long-term in self-reported function in favour of PASTOR compared to MOR in a well-defined and homogeneous study population with CLBP and moderate functional limitations. Whether these effects also occur in more impaired individuals with CLBP cannot be answered and should be investigated in future studies. The reported difference between both groups as well as the longitudinal changes within each group encourage further exploration into how inpatient rehabilitation programs with a biopsychosocial approach should be designed to improve the treatment effectiveness and which individuals with CLBP might benefit most from such an intensive treatment approach. From our point of view, using the terms interprofessional and interdisciplinary rehabilitation versus multidisciplinary rehabilitation is not just a different label for the same treatment. Therefore, a clear definition and description of interprofessional and interdisciplinary biopsychosocial rehabilitation programs, their key components, and underlying approaches is necessary to gain deeper insights into the mechanisms of treatment action. Research designs, which allow the exploration of causal mechanisms for treatment effectiveness of such programs, are also required.

The comparability of the German health care setting with 3-week inpatient rehabilitation programs with health care settings of other countries might be limited. In most other countries rehabilitation programs are either provided in an outpatient setting over a longer time period (>5 weeks) or as a combination of inpatient and outpatient treatment also over a longer period. Therefore, the generalisability of our results to other health care settings might be limited. However, the implementation of a high quality interprofessional and interdisciplinary rehabilitation program with a biopsychosocial approach combined with a systematic patient selection approach is realisable in different health care settings and is a promising way to improve the effectiveness in the treatment of CLBP.

In sum, PASTOR improved functional ability and a wide range of secondary outcome measures in the long-term to a greater extent compared to MOR. However, the reported effect sizes are small and the clinical relevance remains discussable. PASTOR seems to be one step into the right direction to improve the effectiveness of inpatient rehabilitation in the treatment of participants with CLBP. Further insights into mechanisms of action of complex intervention programs are required.

## Supporting Information

S1 Checklist TREND Checklist. (PDF)

S1 Protocol Trial Protocol. (PDF)

S2 Protocol Trial Protocol. (PDF)

S1 Intervention Control group. (PDF)

S2 Intervention Intervention group. (PDF)

S3 Intervention Intervention group therapy plan. (PDF)

S1 Measurement Instruments (PDF)

S1 Table LMM results secondary analysis. (PDF)

S1 Ethical Approval Ethical Approval German. (PDF)
