# Supplementary material for: Long-Term Effects of Interprofessional Biopsychosocial Rehabilitation for Adults with Chronic Non-Specific Low Back Pain: A Multicentre, Quasi-Experimental Study
Source: PLoS One. 2015 Mar 13;10(3):e0118609. doi: 10.1371/journal.pone.0118609 (PMC4359119; doi:10.1371/journal.pone.0118609)
Supplement: S1 Protocol — (PDF) [file pone.0118609.s002.pdf]

To the office of the
Ethical review committee of the
Medical Faculty at
Friedrich-Alexander-
University of Erlangen-Nürnberg
Krankenhausstr. 12
91054 Erlangen

Application
to the Ethical review committee of the Medical Faculty

Please fill in the German language. Please tick as appropriate. For multi-centre studies with „Pre-votum" a competent Ethical review committee formed under state law, a shortened application is available under: http://www.ethik.med.uni-erlangen.de *(connection votum)*

Application for assessment of a research project
(No drug examination)

Please submit 9-fold, including attachments as well as 1 time as electronic version

Title of the project:
„Interprofessional Biopsychosocial Rehabilitation to Optimize Inpatient Multidisciplinary Orthopedic Rehabilitation for Chronic Low Back Pain "

I. Project management

1. Name of the principal investigator responsible at the University of Erlangen-Nürnberg: Prof. Dr. phil. Klaus Pfeifer

   Working at: Institute of Sport Science and Sport, Division Exercise and Health
   Telephone: +49 (0)9131.85.28 106
   E-Mail: klaus.pfeifer@fau.de

   Sport scientist, key areas: rehabilitation, health-related physical activity and exercise, Chair „Exercise and Health",
   Experience in examination: number of studies: responsible for 10, partly responsible for approximately 40

2. a) Further researchers on-site:
   Jana Hofmann, M.A. (Research asisstance)

   b) Further centres (in case of multicentric studies)
   University of Wuerzburg
   Department of Medical Psychology, Medical Sociology, and Rehabilitation Sciences Würzburg
   Chair: Prof. Dr. med. Dr. phil. Hermann Faller
   Licence to practise medicine: Heidelberg, 1981
   Experience in examination: number of studies: responsible for: 11, partly responsible for approximately 50

Further researchers on-site:
Dr. Heiner Vogel, Dr. Karin Meng, Dipl.-Psych. Jana Buchmann

Participating rehabilitation centres:
- Asklepios Klinik Schaufling (CA Dr. med. H. Bork)
- Frankenklinik Bad Kissingen (CA Dr. med. E. Kottmann)
- Klinik Franken Bad Steben (CA Dr. med. B. Geigner)
- Klinik Porta Westfalica Bad Oeynhausen (CA Dr. med. Ostermann)

3. Does this application refer to a project that has already been examined by the Ethical review committee of the Friedrich-Alexander-University Erlangen-Nürnberg?
☐ Yes (please provide the code) ☒ No

II. Research project

1. Planned start for the study: 01/01/2008 (information gathering: 01/07/08) // expected end: 31/12/2010
Length of participation in the study for every single person: 12 months, 3 weeks

2. **Abstract of the project** *(maximum of 1.5 pages):*

Objective.The objectives of the present project are the development of a specific interprofessional rehabilitation program to promote active self-management of chronic non-specific low back pain, as well as to compare its effectiveness with the current multidisciplinary orthopaedic rehabilitation.
Research questions. The primary research question refers to the effectiveness of the interprofessional rehabilitation program compared to the multidisciplinary orthopaedic rehabilitation. The focus is directed on factors that relate to active self-management, e.g. self-reported functional abillity, pain related cognitions, physical activity. *Primary hypothesis:* Adults with chronic non-specific low back pain who participate in an interprofessional rehabilitation program will show significantly improved functional ability 12 months after completion of the program in comparison to adults with chronic non-specific low back pain who participate in the standard multidisciplinary orthopaedic rehabilitation. Secondary hyptheses refer to mechanisms of action of the interprofessional rehabilitation program and its effectiveness regarding important risk factors for the development of chronic non-specific low back pain (Yellow Flags).
Study design/ methods. A multicentre prospective quasi-experimental control group design with three time points of measurement will be performed. Temporal sampling with consecutive inclusion of adults with chronic non-specific low back pain are first assigned to a control condition (usual care; n = 294), and after subsequent implementation of the interprofessional rehabilitation program, to an intervention condition (interprofessional rehabilitation, n = 294). To evaluate the effectiveness data of participant are recorded at baseline, discharge, and 12 months.
Description of the interventions. The central objective of inpatient multidisciplinary orthopedic rehabilitation (MOR) (control group) is to improve functional health with the main focus on restoring and improving work ability. A MOR lasts on 23 days with a total extent of therapy of 48 hours on average. MOR is provided by a multiprofessional team consisting of physicians, psychologists, sport therapists, physiotherapists, occupational therapists, masseurs, social workers, dieticians and nurses. The interventions are carried out mainly in open groups. Multidisciplinary rehabilitation includes interventions from the physical and psychological dimensions: health education, exercise therapy back school,

physical treatments, psychological interventions in groups and individual counselling, as well as rehabilitation/social counselling.
The intervention group consists of an interprofessional and interdisciplinary inpatient rehabilitation program with a biopsychosocial approach. The program is matched to the MOR with respect to the total duration and total extent of therapy, the included professions and the interventions dimensions (physical, psychological).This program, that is based on existing preliminary work (AG Rehabilitation, 2006), is carried out on twelve days during a three-week rehabilitation. The overall objective of PASTOR is the development of active self-management of chronic non-specific low back pain. The program covers six interprofessional therapy modules with sessions of 30 to 90 minutes in duration: Education about low back pain (ELBP), Behavioural exercise therapy 1 (BET 1), Behavioural exercise therapy 2 (BET 2), Coping with pain (CWP), Relaxation (R), and Workplace related information (WRI).
The differences between interprofessional rehabilitation and multidisciplinary orthopedic rehabilitation are characterized by, a) an integrative combination of profession related modules within a comprehensive and consistent treatment approach, b) an interprofessional and collaborative teamwork based on profession related modules, c) the use of standardized methods, media and materials by all professions in the therapeutic team d) a highly structured and detailed manual for the entire treatment process. The modules are carried in fixed groups with eight to twelve participants.
Outcomes. Primary outcome is the level of functional ability 12 months after the end of inpatient rehabilitation measured by the Hannover Functional Ability Questionnaire (FFbH-R). Secondary outcome measures include pain-related cognitions, emotions and behaviour, pain intensity, physical activity, health-related quality of life, time off work and medical visits due to low back pain during the last six month.
Sample. N=588; Participants with chronic non-specific low back pain with the following assignment diagnoses M54.4 – M54.9 as well as M51.2 – M51.9 and M53.8 – M53.9 according to ICD-10.
Sample Size and Power Calculation. Sample power (Software: G-Power 3.0) concerning the primary outcome is approximated for an analysis of covariance at t3 with a small effect size of Cohen’s d=0.3, a 2-sided $\alpha=0.05$ and a test power of $1-\beta=0.8$. This approximation results in a sample size of at least 352 participants with complete data for both study groups (n=176 per group). Anticipating a dropout rate of 40%, it is necessary to include 588 participants (n=294 per group).
Data acquisition, -keeping and –transfer.
Potential participants in the control phase as well as in the intervention phase who met the inclusion criteria were identified and informed about the study by their rehabilitation physician during the medical interview at the beginning of the inpatient rehabilitation period. In the preparation phase, all employees of the participating rehabilitation centers will receive intensive training in respect of all procedures that are relevant to the recruitment process. Structural and process procedures for the use of the patient information and informed consent, distribution and collection of questionnaires as well as its sending to the study centre will be rehearsed and automated; the entire questionnaire survey will be done by the rehabilitation centres. The scientific research institutes (University of Erlangen, University of Würzburg) only receive anonymous data.

3. **Study related interventions** and all necessary deviations from the usual routine:

The interprofessional rehabilitation program shows formal and didactic deviations from the standard multidisciplinary orthopedic rehabilitation. The modules are carried out in fixed groups with eight to twelve participants within a comprehensive and consistent treatment approach. In each rehabilitation centre, the sessions of the six modules were distributed over 12 days during the inpatient rehabilitation period, which lasted on

average three week. By means of an intensive interprofessional biopsychosocial training for all health professions before the start of the program and regular team meetings during the rehabilitation process close exchange-/cooperation relations are promoted between the involved professions. Furthermore a modification of the weighting of single interventions and contents are achieved compared with the usual care. For example, standard exercise therapy often follows a biomedical approach to improve physical fitness. In contrast, the behavioral exercise therapy module within the interprofessional rehabilitation program is directed to improve positive exercise experiences, to reduce pain related cognitions, as well as to promote a physically active lifestyle. Furthermore, the training and practice of active coping strategies to improve self-management of chronic non-specific low back pain for a sustained healthy acitve lifestyle are important key components of the interprofessional rehabilitation.
The interprofessional rehabilitation program is carried out in a standardised form and in fixed groups, so that in comparison to the daily routine individual differences in the quality and quantity of the rehabilitation treatment hardly will occur. The interindividual variability is low.

4. Will the study be carried out according to the recommendations of the World Medical Association (Declaration of Helsinki, revised version on the 48th WMA General Assembly, Somerset West, Republic of South Africa, October 1996) Please indicate if any other evaluation options have been exhausted.

This study is conducted according to the recommendations of the World Medical Association (Declaration of Helsinki, revised version on the 52nd WMA General Assembly, Edinburgh, Scotland, October 2000), as well as to the Guidelines for good clinical practice (ICH). Information for the participants about aims, content and further key aspects of the study and their agreement on study participation will be included in the „informed consent", a written document. Furthermore they are informed that participation in the study is voluntary, refusal to participate will invole no disadvantages or loss of benefits, the withdrawal of the study is possible at any time without loss of benefits, and the data processing is anonymous. According to the national data protection laws all personal data were treated as confidential and were used only for scientific purposes. Individual data (assignment lists, pseudonymisation) are not combined with the data set and remain within the participating rehabilitation centres; questionnaires are forwarded anonymously to the scientific research institutes (University of Erlangen, University of Würzburg). After finishing the study the assignment list will be deleted in order to anonymise the study data.

There is no alternative evaluation possibility!

5. Mode of the research project:
Is the research project a
- ☐ A diagnostic test?
- ☒ A therapeutic test?
- ☐ A compatibility test?
- ☐ A solely scientific experiment?

6. Legal foundations
a) Is this study about an experiment designed to explore or detect clinical or pharmacological effects of medcation or to identify side effects or to examine resorption, distribution, metabolism or excretion **with the aim to gain information about the harmlessness or effectiveness of the medication** (clinical testing of medication according to §§ 40 Medicines Act)?
No, this is not the case!

Please give reasons. Explanations to the application form for evaluation of clinical medicine testing according to §§ 40 AMG can be found under the following link http://www.ethik.med.uni-erlangen.de/AMG-Studien.htm

b) Is this study about a clinical test according to §§ 20 Medizinproduktegesetz (Medicine product Act)?
☐ Yes ☒ No

Please describe. Does a CE certification of the medcal product exist? Are there any additional invasive or other straining experiments being carried out?

c) Does this study contain plans according to §§ 8 Gesetz zur Regelung des Transfusionswesens (German regulation of transfusion Act)?
☐ Yes ☒ No

7. Is this study about an experiment according to

§§ 23 of the German Radiation Protection Act?	☐ Yes	☒ No
§§ 28 of the German X-ray regulations?	☐ Yes	☒ No

Please describe. In case the answer is yes: Will the necessary official expert's reports be obtained?

8. Study type:
☒ Open
☐ Blind
☐ Doubleblind
☒ Comparing
☐ Randomised
☒ Multicentric
☒ Field study
☐ Pilot study

9. Scientific/academic rationale of the project, in particular:

a. Aims of the study

Based on a structured rehabilitation program with a biopsychosocial approach and an integrative linking of different treatment components, the major aim of the present study is to evaluate the long-term effectiveness of such an interprofessional rehabilitation program compared to the standard multidisciplinary orthopedic rehabilitation (usual care) to promote self-management (e.g. functional ability, quality of life, pain related cognitions) of chronic non-specific low back pain.

b. Presentation of the current state of knowledge

International reviews recommend intensive multidisciplinary biopsychosocial rehabilitation in cases of disabling chronic non-specific low back pain (Guzmán et al. 2004, Hayden et al. 2005, Ostelo et al. 2005, van Tulder et al. 2003, Schonstein et al. 2003). In contrast, to date there ar only few national intervention studies, that have evaluated the effectiveness of multidisciplinary biopsychosocial rehabilitation for the treatment of adults

with disabling chronic non-specific low back pain. Two uncontrolled studies with a pre-post design report several positive effects of outpatient multidisciplinary biopsychosocial rehabilitation in adults with chronic non-specific low back pain (Schöps et al. 2000, Pfingsten and Hildebrandt, 2001). Furthermore, two reviews summarise the currently available evidence for the effectiveness of multidisciplinary rehabilitation in Germany (Hüppe and Raspe 2003, 2005). In the mostly uncontrolled studies only short-term intervention effects (pre-post) are reported, usually with very low effect sizes. Hüppe and Raspe (2005) underline, that controlled studies which assess the mid- and long-term effects of a multidisciplinary rehabilitation compared to no treatment or usuale care are lacking. Only one controlled study compared multidisciplinary biopsychosocial rehabilitation with multidisciplinary orthopedic rehabilitation in the German rehabilitation setting, and reported significant effects on function and pain after ten months in favour of the multidisciplinary biopsychosocial rehabilitation. Compared to a functional restoration approach with standard exercise therapy to improve physical fitness, especially resource-oriented knowledge and behavior modification, as well as to promote positive experiences with exercise seem to be important components to improve long-term effectiveness (e.g. Kleist et al. 2001). Further preliminary results of on-going studies which assess the effects of multidisciplinary biopsychosocial rehabilitation named „behavioral medical rehabilitation“ compared to standard multidisciplinary rehabilitation also point in this direction (Mehnert et al. 2007, Schwarz et al. 2007). Overall, the few so far existing monocentric studies indicate that the expenditure to implement an interprofessional rehabilitation program is feasible and promising. Nonetheless, the purposeful integrative linking of knowledge-, behavior- and activity-related interventions has not been established in clinical routine so far. The development of national disease management guidelines (KTL evaluations, Gülich et al. 2003) as well as formative evaluations of the health training concept of the German pension insurance (Worringen et al. 2006) imply quality and quantity deficits in the implementation of patient education in medical rehabilitation.

10. Details of Benefit-Risk-Relation

a. What are the expected benefits of the study?

aa) For participants?

The interprofessional rehabilitation program includes evidence-based interventions to manage chronic non-specific low back pain, and it considers well-defined quality criteria for patient education programs in Germany. Furthermore it is characterized by a stronger patient orientation and therefore it is supposed to improve the individual success of treatment.

Chronic non-specific low back pain is a leading cause of disability worldwide. It affects all areas of life, e.g. social and work participation. Several theories and models, as well as results of randomised controlled trials emphasize the interplay between biological, psychological and social factors in the development of chronic pain (e.g. fear-avoidance model). Beyond simple additive effects, this refers especially to the relevance of mutually reinforcing between these factors. It is assumed, that the implementation of an interprofessional rehabilitation program improves self-management competencies to a greater extent compared to the standard multidisciplinary orthopaedic rehabilitation. This covers, for example, reduced fear-avoidance-beliefs, reduced further pain related cognitions (e.g. catastrophizing), adaptive pain coping strategies, improved self-efficacy, and the promotion of a physically active lifestyle to reduce symptoms of physical deconditioning.

Consequently, expected long-term results among the mayority of participants of the intervention group are: improved self-reported functional ability, reduced pain intensity, and improved quality of life.

ab) for medicine?

- Introduction as interprofessional rehabilitation program for the German pension insurance
- Development of trainer manuals for the implementation of the interprofessional rehabilitation program in the routine of inpatient rehabilitation (e.g. contents, media, patient information, organisational assistance)
- Development and implementation of a training program for the rehabilitation team (Train-the-Trainer), transferring of the concept into curricula of relevant German organisations (e.g. DGOOC, DVGS, ZVK)
- Optimisation of quality and quantity in the inpatient orthopedic rehabilitation of chronic non-specific low back pain by current scientific standards

ac) for science (e.g. results, which do not immediately serve therapeutic purposes)

- Improved evidence on short-and long-term effects of interprofessional rehabilitation programs in chronic non-specific low back pain
- Exploration of underlying mechanisms of action
- Publications in national and international scientific journals

b. **What are the risks for persons who take part in the study?**

ba) What kind of risks? What is the nature oft he risks? Risk estimation, forseeable risks attached to the treatment or any other study-related processes that might be used (including pain, inconvenience, problems, violations of personal integrity and measures to avoid and/or treat unpredictable/unwanted incidents)

- Evidence-based interventions will be carried out. The main focus is on the introduction of an interprofessional approach, as well as on a didactic modification with an interactive education format. There are no risks expected for the study participants.

bb) How probable is it that the risks are realized? How secure can the probability be estimated?

Not applicable.

c. **In your opinion, why is the potential risk defensible in relation to the expected benefit?**

Not applicable.

d. Is there an evaluation of interim findings to recognize a trend?

☐ Yes ☒ No

e. Have been criteria defined that would lead to a change or termination of the study.

☐ Yes, which? ☒ No

11. In case of clinical testings according to MPG ***(not applicable)***

12. a) Is cooperation with a statistician intended?? ☐ Yes ☒ No

b) What statistical methods will be used?

The data analysis follows the usual standards in the evaluation of quasi-experimental control group design. To assess the long term treatment effect of interprofessional rehabilitation against multidisciplinary orthopedic rehabilitation, t-test or analysis of covariance (ANCOVA) respectively of functional ability measured with the FFbH-R at the 12-month follow-up will be used, adjusted for baseline differences, as primary analysis. Further possible baseline differences between both study groups in secondary variables (e.g. socio-demographic, medical) will be also addresses via regression analyses or analysis of covariance (Vickers, 2005). The data analyses will be supported by the methodology consulting of the Department of Medical Psychology, Medical Sociology, and Rehabilitation Sciences Würzburg.

13. a) Is this a multi-centre study (i.e. a study carried out by one single study plan but in different study centres and therefore carried out by different examiners)?
☒ Yes ☐ No

b) Have there been/Are there other places that carry out studies with the same or similar design?/ ☒ Yes, where? ☐ No

Effectiveness studies concerning medical rehabilitation or patient education programs implemented in medical rehabilitation about chronic low back pain (i.a. Bandemer-Greulich et al., 2008; Bosse et al., 2007; Greitemann et al., 2006; Mehnert et al., 2007; Schwarz et al., 2007; Metaanalyse: Hüppe & Raspe, 2003, 2005).

14. Who initiated the study?

Prof. Dr. Klaus Pfeifer (University Erlangen), Dr. phil. Vogel (University Würzburg) and Dr. med. Bork (German Society for Orthopedics and Orthopedic Surgery DGOOC, Asklepios Rehabilitation centre Schaufling)

15. Who funds the study? *(Please indicate if you applied for funding at a third, non-public party. If yes, which amount?)*

This project is funded in the module two of the first funding phase by the German Pension Insurance (Deutsche Rentenversicherung Bund) within the Research funding for care-oriented research "Chronic diseases and patient orientation" (http://www.forschung-patientenorientierung.de/index.php/projects/first-funding-phase/module-two-phase-1/pastor-pfeifer.html) provided by German Federal Ministry of Research (BMBF), German Pension Insurance, Statutory health insurance, and Association of Private Health Insurance e.V. (funds: 311,484.00€).

16. The expense allowance for the assessment (http://www.ethik.med.uni-erlangen.de/kosten.htm) is performed by (please name contact):

Prof. Dr. Klaus Pfeifer; Office: Frau Jutta Preischl, Telephone: +49 (0)9131/85-28790

III. Disclosures on the study participants

1. Number *(in case of comparing studies please indicate the division in groups)*

- 588 participants in total
- 294 study participants each in the intervention and control group

In case of Zero-hypothesis-studies:
Was there a formal estimation of sample size?
☒ Yes ☐ No

2. Age and sex (please indicate the age of the participant and the maximum and minimum limit seen as exclusion criteria)

We aim at a ratio of male to female participants which is representative to the German inpatient rehabilitation of chronic non-specific low back pain. The defined age range includes participants between 18 and 65 years. Age-specific and gender-specific effects of the intervention will be tested.

3. Are the participants:
   - ☐ Healthy people
   - ☐ Pregnant or breastfeeding women
   - ☐ Children and teenagers
   - ☒ Relevant diseased (please indicate desease and it's stage)
   - ☐ Persons who suffer from other diseases? (Especially: psychological diseases which evoque doubts of the legal competence or the capacity to understand the wrongfulness of an act)

Participants with chronic non-specific low back pain with the following assignment diagnoses (ICD-10, German version): M51.2-M51.9, M53.8, M53.9 and M54.4-M54.9. The stage of chronicity is determined during the study; moderator analyses will be performed with regard to the effectiveness of the intervention.

4. Which further criteria of inclusion are intended (e.g. permitted additional medicine)?

   There are no futher criteria of inclusion.

5. Which further criteria of exclusion are intended (e.g. advanced kidney or liver insufficiency, forbidden additional medicine etc.)?

- specific underlying diagnosis of back pain (e. g. radicular symptoms, myelopathy, formerly performed spine surgeries, inflammatory deformations of the spine)
- considerably reduced health status (e.g. comorbidities such as severe heart disease)
- age below 18 years or over 65 years
- inability to speak German
- considerably reduced sight and hearing (not corrected)
- severe psychiatric condition as secondary diagnosis
- current application for early retirement or invalidity pension (§§ 51 SGB V)

6. Are people who have legally or officially been sent to a mental institution supposed to take part in the study?
   ☐ Yes ☒ No

7. Are people who have already taken part in other studies supposed to take part in this study?

☐ Yes ☒ No
How long ago does the last participation have to be?

8. Concerning studies with minors ***(irrelevant)***
-----

9. Insurace of the participants
Are you taking out insurance for the participants?
☐ Yes (*please provide the police with informations about the insurance and the amount of insurance benefit)*
☒ No

10. Obligation to secrecy/ Data privacy protection
Are the doctor's duty to maintain confidentiality and the regulations concerning data privacy protection observed?

Yes, the regulations concerning data privacy protection are observed. The participants are informed about any disclosure of medical details and have to approve to the data transmission; release from obligation to secrecy in this case (see patient information)

11. Payment for participants
Are the participants supposed to get a payment (expense allowence or the like)?
☐ Yes, in the amount of EUR ☒ No

11. How are the participants supposed to be **informed** about the nature, significance and scale of the study?

The patients are provided with information in written (see patient information) and oral form. There will be possibilities to ask questions to the responsible rehabilitation centre employees or to the research team of the study.

12. How are the participants supposed to give their consent to the participation in the study?

Written consent is a requirement for the study participation (see consent).

I know that even after a positive evaluation of the project by the Ethics commission of the Medical Faculty of the University Erlangen-Nürnberg the medical and legal responsibility while carrying out the project stays unreservedly with the management.

Erlangen/Nürnberg
Date ....

Signature of the applicant

(Name in block capitals)

Signature of the management of the institution that is responsible for the project.
Agreeing to the research project:

Date..............

Signature of the management

Institution

____

(Name in block capitals)

EK_Oct06

References

AG Rehabilitation [Böhle, E., Bork, H,. Brüggemann, S., Greitemann, B., Hildebrandt, J., Hofmann, J., Kladny, B., Pfeifer, K] im Experten-Panel der Bertelsmann-Stiftung (2006). Qualitätsmanagement im Versorgungsprozess von Patienten mit Rückenschmerzen. „Best-Practice“ - Empfehlungen zu Zielsetzungen, Inhalten und Methoden der Rehabilitation von Patienten mit chronifizierenden oder chronischen Rückenschmerzen. Projektbereicht bei der Bertelsmann-Stiftung.

Bandemer-Greulich, B., Bosse, B., Fikentscher, E., Konzag, T. A., Bahrke, U. (2008). Wirksamkeit psychologischer Interventionen auf die Schmerzverarbeitung innerhalb einer orthopädischen Rehabilitation von chronischen Rückenschmerzen. Psychther Psych Med, 58, 32-37.

Bosse, E., Fikentscher, E., Bandemer-Greulich, U., Müller, K., Bahrke, U., (2007). "Multimorbide" Rückenschmerzpatienten - Charakterisierung und Erfolg innerhalb der stationären orthopädischen Rehabilitation. Phys Med Rehab Kuror, 17, 197-202.

Greitemann et al. (2006). Integriertes Orthopädisch-Psychosomatisches Konzept zur medizinischen Rehabilitation von Patienten mit chronischen Schmerzen des Bewegungsapparates – Langfristige Effekte und Nachhaltigkeit eines multimodalen Programms zur Aktivierung und beruflichen Umorientierung. Z Orthop, 144, 255–266.

Gülich, M., Engel, E.-M., Rose, S., Klosterhuis, H., & Jäckel, W.H. (2003). Leitlinienentwicklung in der Rehabilitation von Rückenschmerzen – Phase 2: Ergebnisse einer KTL-Analyse. Die Rehabilitation 42, 109-117.

Guzmán et al. (2004). Multidisciplinary bio-psycho-social rehabilitation for chronic back-pain (Cochrane Review). In: The Cochrane Library, Issue 3. Chichester, UK: John Wiley & Sons, Ltd.

Hayden et al. (2005). Systematic Review: Strategies for Using Exercise Therapy To Improve Outcomes in Chronic Low Back Pain. Annual Intern Medicine, 142, 776–785.

Hoffman, B.M., Papas, R.K., Chatkoff, D.K. & Kerns, R.D. (2007). Meta-analysis of psychological interventions for chronic low back pain. Health Psychol. 26 (1), 1-9.

Hüppe, A. & Raspe, H. (2003). Die Wirksamkeit stationärer medizinischer Rehabilitation in Deutschland bei chronischen Rückenschmerzen: eine systematische Literaturübersicht 1980-2001. Rehabilitation 42, 143-154

Hüppe, A. & Raspe, H. (2005). Zur Wirksamkeit stationärer medizinischer Rehabilitation in Deutschland bei chronischen Rückenschmerzen: Aktualisierung und methodenkritische Diskussion einer Literaturübersicht. Rehabilitation 44, 24-33.

Kleist, B. et al. (2001). Work Hardening bei chronisch unspezifischen Rückenschmerzen in der stationären medizinischen Rehabilitation. Praxis Klinische Verhaltensmedizin und Rehabilitation, 14 (54), 145-150

Mehnert, A., Büttner, S., Sauer, C., Willmann, U., Bernhardt, R., Höcker, A., Jacobi, C., Herbold, D. & Koch, U. (2007). Wirksamkeit eines integrierten verhaltensmedizinischen orthopädischen Rehabilitationskonzepts hinsichtlich psychosozialer Erfolgsparameter – eine multizentrische Evaluationsstudie. In: Deutsche Rentenversicherung Bund (Hrsg.). Gesund älter werden – mit Prävention und Rehabilitation. DRV-Schriften: Bd. 72, S. 379-381.

Ostelo R.W.J.G., van Tulder, M.W., Vlaeyen, J.W.S., Linton, S.J., Morley, S.J. & Assendelft, W.J.J. (2005). Behavioural treatment for chronic low back pain. The Cochrane Database of Systematic Reviews, Issue1.

Pfingsten, M. & Hildebrandt, J. (2001). Die Behandlung chronischer Rückenschmerzen durch ein intensives Aktivierungskonzept - eine Bilanz von 10 Jahren. AINS 36: 580-589.

Schonstein et al. (2003. Work conditioning, work hardening and functional restoration for workers with back and neck pain (Cochrane Review) In: The Cochrane Library, Issue 3, Oxford: Update Software.

Schöps et al. (2000). Das Münchner Rücken-Intensiv-Programm (MÜRIP). Phys Med Rehab Kuror 10, 120-126.

Schwarz, S., Mangels, M., Holme, M. & Rief, W. (2007). Langzeitevaluation eines verhaltensmedizinischen Ansatzes in der orthopädischen Rehabilitation – eine randomisierte, kontrollierte Studie. In: Deutsche Rentenversicherung Bund (Hrsg.). Gesund älter werden – mit Prävention und Rehabilitation. DRV-Schriften: Bd. 72, S. 381-384.

van Tulder et al. (2003). Exercise therapy for low back pain (Cochrane Review) In: The Cochrane Library, Issue 3, 2003. Oxford: Update Software.

Vickers, A.J. (2005). Analysis of variance is easily misapplied in the analysis of randomized trials: a critique and discussion of alternative statistical approaches. Psychosomatic Medicine, 67, 652-655.

Worringen, U., Reinecke, A. & Mühlig, S. (2006). Das Gesundheitstrainingsprogramm der Deutschen Rentenversicherung Bund. RV aktuell, 53, 497-503.

Attachments

FRIEDRICH-ALEXANDER
UNIVERSITÄT
ERLANGEN-NÜRNBERG

PHILOSOPHISCHE FAKULTÄT
UND FACHBEREICH THEOLOGIE

Institute of Sport Science and Sport,
Division Exercise and Health
University of Erlangen-Nürnberg
Prof. Dr. phil. Klaus Pfeifer

Julius-Maximilians-
UNIVERSITÄT
WÜRZBURG

Department of Medical Psychology, Medical Sociology,
and Rehabilitation Sciences,
University of Würzburg
Prof. Dr. med. Dr. phil. Hermann Faller

Rehabilitation centre

Medical management

14.04.2008

Participant information sheet

For the research project „Interprofessional Biopsychosocial Rehabilitation to Optimise Inpatient Multidisciplinary Orthopaedic Rehabilitation for Chronic Low Back Pain“

Dear Participant,

We are very pleased to welcome you in our rehabilitation centre in [name]. You will receive all necessary and appropriate investigations and treatments that contribute to a significant improvement or restoration of your health and performance.

We are constantly striving to adapt our treatment program on the latest findings, and we also want to make a contribution. Therefore, we support the research project "Interprofessional Biopsychosocial Rehabilitation to Optimise Inpatient Multidisciplinary Orthopaedic Rehabilitation for Chronic Low Back Pain ", which is carried out by the Institute of Sport Science and Sport, University of Erlangen-Nürnberg and the Department of Medical Psychology, Medical Sociology, and Rehabilitation Sciences of the University of Würzburg in our rehabilitation centre.

Physicians and other employees of our rehabilitation centre are actively involved in the collection of your data and may disclose these informations to the two research institutes for their use in this research study. We kindly ask you to support us and therefore the research project.

Your participation in this study is voluntary (see information about voluntary nature). Before you decide whether or not to take part in the study you should read this information. It is very important that you read and understand the following participant information sheet. This form explains the study process.

Description of the research project

Prior to the treatment we will determine whether you are eligible to participate in the research project. If you are eligible and you decide to participate, data are collected as follows:

The process is based on your medical data for this rehabilitation, as well as on your data collected during the rehabilitation stay.

This also includes information that has been sent to our rehabilitation centre by third parties (e.g. payers or social service providers) to carry out the rehabilitation. These informations are taken from your medical record by employees of the rehabilitation centre. Furthermore, data collected during the study such as the exact procedure of your treatment from the treatment plan are needed. Without such data, the entire research project is not useful to carry out. Because information about you and your health is personal and private, it generally cannot be used in this research study without your written authorization. If you sign this form, it will provide that authorization (see Informed Consent Form).

The aim of this study is to evaluate the effectiveness of a rehabilitation program in the management of adults with chronic low back pain that has been developed according to the latest state of knowledge.

This rehabilitation program is characterized, by the fact that you will be jointly supervised together with other participants in unified groups. The different treatment elements (e.g. medical treatment, exercise therapy, relaxation, and practice of useful pain coping strategies) as well as the actions of the rehabilitation team are therefore particularly closely aligned with each other. The emphasis is on the use of therapy sessions with specific and coordinated contents, methods, media and materials in the treatment of chronic low back pain.

The study takes place in two phases. If you decide to participate in the study, you will be assigned depending on the study phase, either the rehabilitation centre‘s or the newly developed treatment program. If you do not wish to take part in this study, you will be provided with the established standard treatment available at our rehabilitation centre [name]. You will be provided with treatments (e.g. exercise therapy, physical therapy) to approximately the same extent as in the newly developed treatment program. Both treatment programs - our own and the newly developed - are equally appropriate to improve your health.

If you decide to participate in our research project, we would like to ask you to fill out a questionnaire at the beginning and end of the rehabilitation stay. Filling out a questionnaire is expected to take around 45 minutes of your time.

After a period of one year, we would like to ask you about your situation again. For this purpose, you will receive a questionnaire from our rehabilitation centre by post with a request to fill these and to send it back in the enclosed envelope to the research institute.

All your data will be collected under a research number that is printed on the questionnaire (see data protection information). Before you decide whether or not to take part in the study, we will explain the study process, responsible persons, as well as data flow and data protection.

Data protection information

All your information obtained in this study will be kept and handled in a confidential manner, in accordance with applicable laws and/or regulations. For this study, the research team from the University of Erlangen and Würzburg will not need to access information that can identify you

such as your name and address. As required by the data protection laws, data from you that will be collected during the study will be kept strictly separate from the information that can identify you.

After you have decided to participate in this study and you have signed the consent, rehabilitation centre employees involved in the research project record your name and address in a continuous numbered list (assignment list). The number (research number) before your name is the unique numerical code under which all your data will be collected. Thus, any information about you will have a code on it instead of your name. The assignment list will be kept in a secure place in the rehabilitation centre and access limited to rehabilitation centre employees. It will not be disclosed to the research team or to third parties such as the German pension insurance. Their only purpose is to establish contact with you and to collect your data under the code.

At the end of data collection, after about two years, we will delete information that can identify you (assignment list and the Informed Consent Form). Thus, no one is able to link the code with you anymore.

Only coded data, so called anonymised data, from the study will be transmitted to the research institutes for the purpose of analysis and will be archived, but your identity will not be revealed at any time.

The results of this research study may be presented at scientific or medical meetings or published in scientific journals. However, your identity will not be disclosed.

Information about voluntary nature

Participation in this research project is entirely voluntary. Your data will only be used, if you have signed the Informed Consent Form.

You may decline to participate or are free to withdraw from this study at any time without reason, without penalty or loss of benefits to which you are otherwise entitled; it is possible even when the Informed Consent Form has been signed.

Your decision to decline to participate or withdraw will not affect the standard of care you receive. If you do not wish to participate or if you withdraw from the trial, neither your current nor future rehabilitation treatment will be adversely affected.

If you do not wish to participate or if you withdraw from the trial, we will delete your name in the above described assignment list, and we will not contact you anymore.

If you withdraw, any data collected (e.g. questionnaires) from you up to your withdrawal will still be used for the study unless you declare otherwise.

We ask for your participation!

If you have read and clearly understand the foregoing informations and you decide to participate in this research project, we ask you to sign the attached Informed Consent Form and submit it to the office respectively to throw it into the provided answer box.

You can keep this participant information sheet in any case. You will be also given a copy of the Informed Consent Form.

If you have any questions, concerns, or complaints about this research project, its procedures, risks or benefits, or alternative courses of treatment, you should ask the below mentioned employees of the rehabilitation centre or [name 1] and [name 2] of the research team of the Universities of Erlangen-Nürnberg and Würzburg. You may contact these persons now or later.

Contact persons of the research team

Division Exercise and Health at the University Erlangen-Nürnberg

[Name, telephone, email] Prof. Dr. Klaus Pfeifer

Department of Medical Psychology, Medical Sociology, and Rehabilitation Sciences, University Würzburg

[Name, telephone, email] Prof. Dr. Dr. Hermann Faller

Contact persons of the rehabilitation centre

[Name, telephone, email]

Prof. Dr. Klaus Pfeifer	Prof. Dr. Dr. Hermann Faller	Medical management

FRIEDRICH-ALEXANDER UNIVERSITÄT ERLANGEN-NÜRNBERG
PHILOSOPHISCHE FAKULTÄT UND FACHBEREICH THEOLOGIE

Institute for sport science and sport,
Division Exercise and Health
University of Erlangen-Nürnberg
Prof. Dr. phil. Klaus Pfeifer

Julius-Maximilians-UNIVERSITÄT WÜRZBURG

Department of Medical Psychology, Medical Sociology,
and Rehabilitation Sciences,
University of Würzburg
Prof. Dr. med. Dr. phil. Hermann Faller

Rehabilitation centre

Medical management

Informed Consent Form

Full name: ______________________________ **Birth date: ___________________**

I have been informed about the purpose and nature of the study: ***„Interprofessional Biopsychosocial Rehabilitation to Optimise Inpatient Multidisciplinary Orthopaedic Rehabilitation for Chronic Low Back Pain“***.

This study is carried out in cooperation with the rehabilitation centre [name] and the Universities of Erlangen-Nürnberg and Würzburg. I have read and I understand the participant information sheet that describes the purpose and nature of this study.

I understand that personal information (name, birth date, address) or any other information that can identify me will be treated as strictly confidential and will not be disclosed to unauthorized third parties.

I understand that relevant sections of my medical data, data collected during the study (e.g. treatment plan), and data provided by the German Pension Insurance, may be looked at by employees of the rehabilitation centre [name], where it is relevant to my taking part in this research. I give permission for these employees to have access to the data. I understand that the data will be given to the scientific research institutes in an anonymous form where any information about me will have a code on it instead of my name. I have been informed that personal information will be deleted if they are no longer required for data collection.

I want to support the research project through my participation and agree to complete questionnaires that are given or sent to me.

I agree to take part in the above study.

Place and date: _______________________

Signature of the Participant: _______________________________

No participation in the study

For organisational reasons we kindly ask you to inform us if you do not wish to participate in this research project.
Please submit this sheet to the station [name]. Furthermore, it is of great interest for us to know the reasons for your non-attendance.
All information is strictly voluntary. But, they would help us to plan further studies.

Please tick below the reasons for your non-attendance (multiple answers are possible). Alternatively, you may also like to leave your personal reasons.

- ☐ I would like generally not support research projects.
- ☐ The completion of questionnaires is too laborious.
- ☐ I'm not sure because I do not have enough information.
- ☐ I do not want to participate in a new program.

Other reasons:
____

____

____
