# Supplementary material for: Long-Term Effects of Interprofessional Biopsychosocial Rehabilitation for Adults with Chronic Non-Specific Low Back Pain: A Multicentre, Quasi-Experimental Study
Source: PLoS One. 2015 Mar 13;10(3):e0118609. doi: 10.1371/journal.pone.0118609 (PMC4359119; doi:10.1371/journal.pone.0118609)
Supplement: S2 Protocol — (PDF) [file pone.0118609.s003.pdf]

3807

An die Geschäftsstelle der
Ethik-Kommission
der Medizinischen Fakultät
der FAU Erlangen-Nürnberg
Krankenhausstr. 12
91054 Erlangen

Antrag
an die Ethik-Kommission
der Medizinischen Fakultät

Eingegangen
22. April 2008
Ethik-Kommission
Medizinische Fakultät
Universität Erlangen-Nürnberg

Bitte ***in deutscher Sprache*** *ausfüllen,*
Zutreffendes bitte ankreuzen.
Für multizentrische Studien mit Vorvotum einer nach Landesrecht gebildeten zuständigen Ethik-Kommission können Sie das verkürzte Antragsformular verwenden, abzurufen unter: http://www.ethik.med.uni-erlangen.de *(Anschlussvotum)*

Antrag auf Beurteilung eines Forschungsprojektes (keine Arzneimittelprüfung)

bitte 9-fach einschließlich Anlagen einreichen sowie 1-mal in elektronischer Fassung

Titel des Projektes:
„Integrative Patientenschulung zur Optimierung der stationären Rehabilitation bei chronischem Rückenschmerz"

I. Projektleitung

1. Name des verantwortlichen Projektleiters an der FAU Erlangen-Nürnberg:
Prof. Dr. phil. Klaus Pfeifer

tätig im: Institut für Sportwissenschaft und Sport, AB Bewegung und Gesundheit
App.-Nr. 09131/8528-106
E-Mail klaus.pfeifer@sport.uni-erlangen.de

Sportwissenschaftler, Schwerpunkt: Rehabilitation, Gesundheitssport,
Professur für Bewegung und Gesundheit,
Prüferfahrung: Studienzahl : verantwortlich 10, mitverantwortlich ca. 40

2. a) Weitere Teilnehmer/innen vor Ort:
Jana Hofmann, M.A. (wissenschaftliche Mitarbeiterin)

b) Weitere Prüfzentren (bei multizentrischen Studien):
Universität Würzburg
Institut für Psychotherapie und Medizinische Psychologie
AB Rehabilitationswissenschaften
Leitung: Prof. Dr. med. Dr. phil. Hermann Faller
Approbation als Arzt: Heidelberg, 1981
Prüferfahrung: Studienzahl: verantwortlich 11, mitverantwortlich ca. 50

Weitere Untersucher vor Ort:
Dr. Heiner Vogel, Dr. Karin Meng, Dipl.-Psych. Jana Buchmann

Kooperationskliniken:
- Asklepios Klinik Schaufling (CA Dr. med. H. Bork)
- Frankenklinik Bad Kissingen (CA Dr. med. E. Kottmann)
- Klinik Franken Bad Steben (CA Dr. med. B. Geigner)
- Klinik Porta Westfalica Bad Oeynhausen (CA Dr. med. Ostermann)

3. Handelt es sich bei diesem Antrag um ein bereits von der Ethik-Kommission der Medizinischen Fakultät der Friedrich-Alexander-Universität Erlangen-Nürnberg begutachtetes Projekt?
☐ ja (bitte lfd. Nr. angeben) ☒ nein

II. Forschungsvorhaben

1. Geplanter Beginn der Studie: 01.01.2008 (Datenerhebung: 01.07.08)
// voraussichtliches Ende: 31.12.2010
Dauer der Studienteilnahme für den einzelnen Probanden: 12 Monate, 3 Wochen

2. Kurzer Abriss des Projektes *(maximal 1,5 Seiten)*:

Zielsetzung. Gegenstand des Projekts ist zum einen die Entwicklung eines spezifischen integrativen Patientenschulungsprogramms zum Aufbau von Selbstmanagementkompetenzen bei Patienten mit chronischen Rückenschmerzen und zum anderen die Evaluation dessen Wirksamkeit im Vergleich zum derzeit üblichen Vorgehen in der stationären Rehabilitation.
Fragestellungen. Die zentrale Fragestellung des Projekts bezieht sich auf die Wirksamkeit des integrativen Patientenschulungsprogramms im Vergleich zur Standardrehabilitation. Im Mittelpunkt der Betrachtung steht dabei das Ausmaß der relativen Auswirkungen auf, für das aktive Selbstmanagement relevante Faktoren, wie z.B. schmerzbedingte Funktionseinschränkungen, schmerzbezogene Kognitionen oder der Aufbau eines körperlich aktiveren Lebensstils.
Primäre Hypothese: Die Teilnahme an einem integrativen Patientenschulungsprogramm führt in der stationären Rehabilitation von Menschen mit chronischen Rückenschmerzen im Vergleich zum derzeit üblichen Vorgehen (usual care) zu einer stärkeren Reduktion von rückenschmerzbedingten Funktionseinschränkungen. *Nebenfragestellungen* betreffen die Wirkmechanismen des Schulungsprogramms sowie dessen Wirksamkeit in Abhängigkeit empirisch nachgewiesener Risiko- und Chronifizierungsfaktoren (Yellow Flags).
Studiendesign/ Methoden. Die Prüfung der Wirksamkeit erfolgt in einem multizentrischen, quasi-experimentellen Kontrollgruppendesign mit drei Messzeitpunkten. Zeitstichproben mit konsekutivem Einschluss von Patienten mit chronischen Rückenschmerzen werden zuerst einer Kontrollbedingung (usual care; n = 294) und nach anschließender Implementierung des neuen Behandlungsprogramms einer Interventionsbedingung (Integrative Patientenschulung; n = 294) zugewiesen. Patientendaten zur Prüfung der Wirksamkeit werden zu Rehabilitationsbeginn, -ende und in einer 12-Monats-Katamnese erfasst.
Beschreibung der Intervention. Die Intervention besteht aus einem spezifischen integrativen, d.h. interdisziplinär und multiprofessionell aufeinander abgestimmten Patientenschulungsprogramm. Das auf der Basis bestehender Vorarbeiten (AG Rehabilitation, 2006) entwickelte Programm wird an zwölf Tagen während einer dreiwöchigen Rehabilitation durchgeführt. Dabei soll sukzessive und in didaktisch sinnvoller Weise die individuelle Selbstmanagementkompetenz im Umgang mit Rückenschmerzen verbessert werden. Das

Programm besteht aus den folgenden fünf Bausteinen: Vermittlung von rückenschmerzbezogenem Wissen (RW), verhaltensbezogene Bewegungstherapie (VBT), Vermittlung von Coping-Strategien für den Umgang mit Rückenschmerz-Episoden sowie motivationaler und volitionaler Aspekte (Cop), Vermittlung eigenständig durchführbarer Entspannungsverfahren (E), Vermittlung arbeitsplatzbezogener Inhalte bzw. Informationen zu sozialmedizinischen Fragen (AI).
Outcomes. Primäres Zielkriterium ist der rückenschmerzbedingte Funktionsstatus ein Jahr nach der Rehabilitation. Sekundäre Zielgrößen sind Angst-Vermeidungsüberzeugungen, schmerzbezogene Kognitionen, Schmerzbewältigung, Rückenschmerz, körperliche Aktivität, psychische Beeinträchtigung, subjektive Gesundheit/Lebensqualität sowie Arbeitsfähigkeit und Inanspruchnahme medizinischer Leistungen.
Stichprobe. N = 588; Rehabilitanden mit chronischen unspezifischen Rückenschmerzen bei Hauptdiagnosen M54.4 – M54.9 sowie M51.2 – M51.9 und M53.8 - M53.9 nach ICD-10
Fallzahlenberechnung. Erforderlich sind 352 Probanden mit vollständigem Datensatz für Interventions- und Kontrollgruppe (bei geschätztem, kleinen Effekt von ES=0,30; angestrebter Signifikanz von 0,05 und Power von 0,80); bei erwartetem Drop Out von 40% müssen 294 Probanden pro Gruppe erreicht werden.
Datenerfassung, -haltung und –transfer. Die Rekrutierung der Studienteilnehmer erfolgt in den beteiligten Kliniken über die Aufnahme der Patienten im Rahmen einer mehrdimensionalen Indikationsstellung bei der ärztlichen Diagnostik sowohl in den Kontroll- als auch in den Interventionsphasen. In der Vorbereitungsphase werden die in den Kliniken tätigen Kolleginnen und Kollegen mit den für den Rekrutierungsprozess notwendigen Abläufen vertraut gemacht. Strukturen und Prozessabläufe für die Nutzung von Patienteninformationen und Einverständniserklärungen, der Fragebogenerhebung sowie deren Übersendung an das Studienzentrum werden erprobt und automatisiert; die Abläufe werden in einem schriftlichen Studienprotokoll definiert und dokumentiert. Die gesamte Fragebogenerhebung erfolgt durch die Kliniken. Die Forschungszentren erhalten lediglich anonymisierte Daten.

3. **Studienbezogene Maßnahmen** und alle erforderlichen Abweichungen von der üblichen Routine-Behandlung:

Das in der Intervention durchgeführte Programm weist formale und didaktische Abweichungen vom Standardprogramm in der medizinischen Rehabilitation auf. Die Durchführung des Programms findet in geschlossenen Gruppen statt und erfolgt auf Basis eines auf die Behandlungstage abgestimmten und in sich geschlossenen didaktischen Aufbaus. Dabei werden die Patienten an 12 Tagen während ihres dreiwöchigen Aufenthaltes mit einem Umfang von 48 Stunden intensiv betreut. Durch interdisziplinäre Schulungen und Teambesprechungen werden engere Austauschs-/Kooperationsbeziehungen zwischen den beteiligten Professionen gefördert. Darüber hinaus kommt es zu einer Veränderung der Gewichtung einzelner Therapiebausteine und -inhalte im Vergleich zur usual care. Beispielsweise beziehen sich die Inhalte und Methoden der Bewegungstherapie des entwickelten Patientenschulungsprogramms im Vergleich zu den üblichen, meist physisch betonten Zugängen insbesondere auf die Vermittlung positiver Bewegungserfahrungen, die Reduktion von Bewegungsangst und den Aufbau von Bewegungskompetenzen für eine eigenständige körperliche/ gesundheitssportliche Aktivität. Des Weiteren stehen die Vermittlung und Einübung aktiver Copingstrategien für den Umgang mit Rückenschmerz sowie die Verbesserung von Selbstmanagementkompetenzen für eine nachhaltigere Veränderung der Lebensgewohnheiten hin zu einem gesundheitlich aktiven Lebensstil im Zentrum der Behandlung.
Das zu prüfende Patientenschulungsprogramm wird in standardisierter Form und in geschlossenen Gruppen durchgeführt, so dass im Vergleich zur Routinebehandlung kaum interindividuelle Unterschiede in der Qualität und Quantität der Behandlungskomposition auftreten werden. Es besteht eine geringe interindividuelle Variabilität.

4. Wird die Studie gemäß der von der 48. Generalversammlung des Weltärztebundes in Somerset West revidierten Deklaration von Helsinki aus dem Jahre 1996 durchgeführt? Bitte angeben, ob alle anderen Erprobungsmöglichkeiten ausgeschöpft wurden.

Die Studie wird gemäß den Empfehlungen des Weltärztebundes (Deklaration von Helsinki, revidierte Fassung der 52. Generalversammlung, Oktober 2000, Edinburgh/Schottland) und den Leitlinien zur Guten Klinischen Praxis durchgeführt. Die Probanden werden in einem Informationsschreiben über die Ziele der Untersuchung und den eigenen Aufwand bei einer Teilnahme informiert und um Mitwirkung gebeten („informed consent"). Des Weiteren wird über die Freiwilligkeit, das Widerrufsrecht, die Anonymität der Datenverarbeitung sowie über einen Ausschluss von Nachteilen bei einer Teilnahmeverweigerung informiert. Alle Daten werden entsprechend der Richtlinien des Datenschutzgesetzes vertraulich behandelt und nur für wissenschaftliche Zwecke verwendet. Patientenbezogene Daten (Zuordnungslisten, Pseudonymisieren) werden nicht mit dem Datensatz zusammengeführt und verbleiben in den beteiligten Kooperationskliniken; die Daten (Fragebögen) werden in anonymer Form an die wissenschaftlichen Forschungsinstitute (Universität Erlangen, Universität Würzburg) weitergeleitet. Nach Beendigung der Studie wird die Zuordnungsliste gelöscht, so dass die Studiendaten anonymisiert sind.

Es sind keine alternativen Erprobungsmöglichkeiten möglich!

5. Art des Forschungsvorhabens:
Handelt es sich um
- ☐ eine diagnostische Prüfung?
- ☒ eine therapeutische Prüfung?
- ☐ eine Verträglichkeitsprüfung?
- ☐ einen ausschließlich wissenschaftlichen Versuch

6. Gesetzliche Grundlagen

a) Handelt es sich um eine Untersuchung, die dazu bestimmt ist, klinische oder pharmakologische Wirkungen von Arzneimitteln zu erforschen oder nachzuweisen oder Nebenwirkungen festzustellen oder die Resorption, die Verteilung, den Stoffwechsel oder die Ausscheidung zu untersuchen, **mit dem Ziel, sich von der Unbedenklichkeit oder Wirksamkeit des Arzneimittels zu überzeugen** (klinische Prüfung eines Arzneimittels nach §§ 40 Arzneimittelgesetz)?
Nein. Trifft nicht zu!

Bitte begründen. Erläuterungen zum Antrag auf Bewertung einer klinischen Arzneimittelprüfung nach § 40 AMG finden Sie unter http://www.ethik.med.uni-erlangen.de/AMG-Studien.htm

b) Handelt es sich um eine klinische Prüfung nach § 20 Medizinproduktegesetz (MPG)?
☐ ja ☒ nein

Bitte begründen. Liegt eine CE-Zertifizierung für das Medizinprodukt vor? Werden zusätzlich invasive oder andere belastende Untersuchungen durchgeführt?

c) Handelt es sich um ein Vorhaben nach § 8 des Gesetzes zur Regelung des Transfusionswesens (TFG)?
☐ ja ☒ nein

7. Handelt es sich um einen Versuch nach

§ 23 Strahlenschutzverordnung?	☐ ja	☒ nein
§ 28 Röntgenverordnung?	☐ ja	☒ nein

Bitte begründen. Falls ja: Werden die hiernach erforderlichen behördlichen Gutachten eingeholt?

8. Typ der Studie:
   - ☒ offen
   - ☐ blind
   - ☐ doppelblind
   - ☒ vergleichend
   - ☐ randomisiert
   - ☒ multizentrisch
   - ☒ Feldstudie
   - ☐ Pilotstudie

9. Wissenschaftliche Begründung des Projekts, insbesondere:

a. Erläuterung des Versuchsziels

Vor dem Hintergrund einer zielgerichteten Verknüpfung von wissens-, verhaltens- und bewegungsbezogenen Interventionsformen soll untersucht werden, in wie fern die Implementierung eines multimodalen, interdisziplinären Patientenschulungsprogramm zum Aufbau von Selbstmanagementkompetenzen im Vergleich zur üblichen Behandlung von chronischen, unspezifischen Rückenschmerzen in der stationären Rehabilitation zu differerierenden, kurz- und längerfristigen Effekten in zentralen Merkmalen, wie der rückenschmerzbedingten Funktionsfähigkeit, der subjektiven Lebensqualität sowie Angst-Vermeidungsüberzeugungen und schmerzbezogene Kognitionen führt.

b. Darstellung des bisherigen Wissensstandes

In internationalen systematischen Übersichtsarbeiten wird eine gute Evidenz für die Wirksamkeit multimodaler und interdisziplinärer Behandlungsprogramme in der Therapie chronischer Rückenschmerzen berichtet (Guzmán et al. 2004, Hayden et al. 2005, Ostelo et al. 2005, van Tulder et al. 2003, Schonstein et al. 2003). Demgegenüber existieren bislang nur wenige nationale Interventionsstudien hoher Qualität, die Ansätze eines interdisziplinären und multimodalen Vorgehens für die Behandlung von Patienten mit Rückenschmerzen umgesetzt und evaluiert haben. Für den Bereich der ambulanten Versorgung liegen z.B. nicht kontrollierte Studien mit einfachem Prä-Post-Design von Schöps et al. (2000) oder Pfingsten und Hildebrandt (2001) vor, die positive Wirkungen entsprechend gestalteter Interventionen zeigen. Die derzeit für den Bereich der stationären Rehabilitation vorhandenen Studien werden in den Übersichtsarbeiten von Hüppe und Raspe (2003, 2005) zusammengefasst und bewertet. In den zumeist nicht kontrollierten Studien werden lediglich kurzfristige Interventionseffekte mit meist relativ niedrigen Effektstärken berichtet. Nachhaltige Effekte konnten in keiner der vorliegenden Studien erzielt werden. Hüppe und Raspe (2005) weisen darauf hin, dass insbesondere kontrollierte Studien fehlen, die bei chronischen Rückenschmerzen mittel- bis langfristige Effekte einer stationären multimodalen Behandlung mit keiner Behandlung bzw. mit der normalen Grundversorgung vergleichen. Lediglich eine erste entsprechende Arbeit wurde

kürzlich von Greitemann et al. (2006) vorgelegt, die eine verbesserte langfristige Wirksamkeit eines zielgerichteten Interventionskonzeptes in einer quasi-experimentellen Vergleichsgruppenstudie in der stationären Behandlung chronischer Rückenschmerzen zeigen konnte. Dabei scheinen insbesondere die Aspekte der ressourcenbezogenen Wissensvermittlung und Verhaltensmodulation (Hoffmann et al. 2007) sowie die Vermittlung positiver Bewegungserfahrungen dem üblichen Ansatz des „Functional Restoration" mit immer noch stark somatischem Bezug (körperliches Training) (z.B. Kleist et al. 2001) überlegen zu sein. Auch erste Ergebnisse der laufenden Studien von Mehnert et al. (2007) oder Schwarz et al. (2007) zu Wirkungen verhaltensmedizinischer Interventionen weisen in diese Richtung. Insgesamt deuten die wenigen, bislang vorliegenden monozentrischen Studien daraufhin, dass der mit der Einführung entsprechender Konzepte notwendige organisatorische Mehraufwand in den Einrichtungen leistbar und Erfolg versprechend ist.
Bislang erfolgt in der Praxis der rehabilitativen Versorgung aber noch keine zielgerichtete Verknüpfung von wissens-, verhaltens- und bewegungsbezogenen Interventionsformen. Die KTL-Auswertungen im Rahmen der Entwicklung von Versorgungsleitlinien zur Rehabilitation von Rückenschmerzen (Gülich et al. 2003) lassen ebenso wie die formative Evaluation der Umsetzung des Gesundheitstrainings der DRV Bund (Worringen et al. 2006) auf Defizite in der Qualität und Quantität der Umsetzung von Patientenschulung in der medizinischen Rehabilitation schließen.

10. Angaben zur Nutzen-Risiko-Relation

a. Welcher Nutzen ist von den Ergebnissen der Studie zu erwarten

aa) für die Versuchsteilnehmer?

Das entwickelte integrative Patientenschulungsprogramms orientiert sich an bestehenden Qualitätskriterien und wissenschaftlicher Evidenz für Interventionen bei Rückenschmerz, beinhaltet eine stärkere Patientenorientierung und sollte somit den individuellen Behandlungserfolg verbessern.
Chronische Rückenschmerzen gehen einher mit einer für das Individuum schwer zu bewältigenden Krankheitslast, die sich äußerst negativ auf sämtliche Lebensbereiche, die soziale und berufliche Teilhabe auswirkt. Empirische Befunde und Theorien zum Prozess der Chronifizierung von Rückenschmerzen sprechen für die Relevanz von sich gegenseitig verstärkenden physiologischen und psychosozialen Faktoren, die über einfache additive Wirkungsgefüge hinausgehen (z.B. Fear-Avoidance-Modell). Es wird angenommen, dass die Implementierung eines multimodalen, integrativen Patientenschulungsprogramm zu einer stärkeren Aktivierung von Handlungsregulationsressourcen führt als die in der stationären Rehabilitation üblichen Behandlungsprogramme. Dazu zählen zum einen die Verringerung von Angst-Vermeidungsüberzeugungen sowie von maladaptiven Bewältigungsstrategien und auf der anderen Seite eine Erhöhung der Selbstwirksamkeit, eine stärkere Identifikation mit bzw. Bindung an körperliche Aktivität und somit die Verringerung von körperlicher Dekonditionierung, eine nachhaltigere Förderung von adaptiven kognitiven und behavioralen Strategien der Schmerzbewältigung.
Infolgedessen ist zu erwarten, dass sich insbesondere für das Gros der Teilnehmer der Interventionsgruppe relativ substantielle und nachhaltige Verbesserungen im Hinblick auf die alltägliche Funktionsfähigkeit, die Schmerzintensität, die schmerzbedingte, psychische Beeinträchtigung sowie die subjektive Lebensqualität im Allgemeinen ergeben.

ab) für die Heilkunde?

- Einführung als Patientenschulungsprogramm für die Deutsche Rentenversicherung

- Erstellung von Manualen/Handbüchern zur Umsetzung des spezifischen Patientenschulungsprogramms in der Praxis der stationären Rehabilitation (Inhalte, Medien, Patienteninformationen, Organisationshilfen etc.)
- Entwicklung und Umsetzung eines Schulungskonzepts für das Reha-Team (Train the Trainer-Seminare), Überführung der evaluierten Konzepte in Ausbildungscurricula von DGOOC, DVGS, ZVK etc.
- Erhöhung der Qualität und Quantität bei der stationären Behandlung von chronischem Rückenschmerz nach aktuellen wissenschaftlichen Standards

ac) für die Wissenschaft (z.B. Ergebnisse, die nicht unmittelbar therapeutischen Zwecken dienen)?

- Erweiterung der empirischen Befundlage zu kurz- und längerfristigen Effekten multimodaler, interdisziplinärer Patientenschulungsprogramme bei chronischem Rückenschmerz
- Überprüfung zugrunde liegender Wirkmechanismen
- Publikationen in nationalen und internationalen wissenschaftlichen Zeitschriften

b. **Mit welchem Risiko ist die Studie für die Versuchsteilnehmer verbunden?**

ba) Welcher Art sind die Risiken? Risikoeinschätzung, vorhersehbare Risiken der Behandlung und sonstiger studienbedingter Verfahren, die eingesetzt werden sollen (einschließlich Schmerz, Unannehmlichkeiten, Beschwerden, Verletzung der persönlichen Integrität und Maßnahmen zur Vermeidung und/oder zur Behandlung von unvorhersehbaren/ unerwünschten Ereignissen)

- Durch die Nutzung erprobter Therapiebausteine und der vorwiegend didaktischen Modifikation des Schulungs- und Rehabilitationsprogramms sind keine Risiken für die Studienteilnehmer anzunehmen.

bb) Mit welcher Wahrscheinlichkeit ist zu erwarten, dass sich die Risiken realisieren? Wie sicher ist die Wahrscheinlichkeit abschätzbar?

Entfällt.

c. **Warum ist das mögliche Risiko im Verhältnis zu dem zu erwartenden Nutzen Ihrer Ansicht nach vertretbar?**

Entfällt.

d. Werden Zwischenergebnisse ausgewertet, um einen Trend zu erkennen?
☐ ja ☒ nein

e. Sind Kriterien festgelegt worden, bei deren Eintreten der Versuch geändert oder abgebrochen werden soll? ☐ ja, welche? ☒ nein

11. Bei klinischen Prüfungen nach MPG: ***(nicht zutreffend)***

a. Welches Medizinprodukt soll geprüft werden?

b. Wird die klinische Prüfung von einer entsprechend qualifizierten und befugten Person geleitet, die mindestens eine zweijährige Erfahrung in der klinischen Prüfung von Medizinprodukten nachweisen kann? ☐ ja ☐ nein

c. Wurde (soweit erforderlich) eine dem jeweiligen Stand der wissenschaftlichen Erkenntnisse entsprechende biologische Sicherheitsprüfung oder sonstige für die vorgesehene Zweckbestimmung des Medizinproduktes erforderliche Prüfung durchgeführt? ☐ ja ☐ nein

d. Wurde (soweit erforderlich) die sicherheitstechnische Unbedenklichkeit für die Anwendung des Medizinproduktes unter Berücksichtigung des Standes der Technik sowie der Arbeitsschutz- und Unfallverhütungsvorschriften nachgewiesen? ☐ ja ☐ nein

e. Ist der Leiter der klinischen Prüfung über die Ergebnisse der biologischen Sicherheitsprüfung und die voraussichtlich mit der klinischen Prüfung verbundenen Risiken informiert worden? ☐ ja ☐ nein

12. a) Ist die Mitarbeit eines Statistikers vorgesehen? ☐ ja ☒ nein

b) Welche statistischen Methoden sollen benutzt werden?

Die Datenanalyse folgt den üblichen Standards bei der Auswertung quasi-experimenteller Kontrollgruppendesigns. Zur Analyse der Wirkeffekte – der Unterschiede zwischen der Kontroll- und der Interventionsgruppe zum dritten Messzeitpunkt – werden t-Test bzw. Kovarianzanalysen durchgeführt, bei denen evtl. vorliegende Baseline-Unterschiede in den Ausgangsmesswerten zwischen Interventions- und Kontrollgruppe als Kovariate in die Berechnung einbezogen werden. Sollten sich die Interventions- und Kontrollgruppe darüber hinaus trotz des gewählten Studiendesigns in bestimmten (soziodemografischen, medizinischen etc.) Variablen unterscheiden, werden diese Variablen über regressions- bzw. kovarianzanalytische Ansätze (Vickers, 2005) als weitere Kontrollvariablen in die Analyse einbezogen.
Die Datenanalyse wird durch die Methodenberatung des Arbeitsbereichs Rehabilitationswissenschaften der Universität Würzburg beratend unterstützt.

13. a) Handelt es sich um eine multizentrische Studie (d.h. eine nach einem *einzigen* Prüfplan durchgeführte Studie, die in mehr als einer Prüfstelle erfolgt und daher von mehr als einem Prüfer vorgenommen wird)? ☒ ja ☐ nein

b) Wurden/Werden an anderer Stelle Studien mit demselben oder einem ähnlichen Ziel durchgeführt? ☒ ja, wo? ☐ nein

Studien zur Wirksamkeit der medizinischen Rehabilitation bzw. von Patientenschulungsprogrammen in der medizinischen Rehabilitation bei chronischem Rückenschmerz (u.a. Bandemer-Greulich et al., 2008; Bosse et al., 2007; Greitemann et al., 2006; Mehnert et al., 2007; Schwarz et al., 2007; Metaanalyse: Hüppe & Raspe, 2003, 2005).

14. Wer hat die Studie initiiert?

Prof. Dr. Pfeifer (Universität Erlangen), Dr. Vogel (Universität Würzburg) und Dr. Bork (Deutsche Gesellschaft für Orthopädie und Orthopädische Chirurgie DGOOC, Asklepios Klinik Schaufling)

15. Wer finanziert sie? (*Bitte geben Sie an, ob Drittmittel von nichtöffentlicher Seite beantragt werden. Falls ja, in welcher Höhe?*)

Die Studie ist Teil des gemeinsamen Förderschwerpunkts des BMBF, der Deutschen Rentenversicherung, der Spitzenverbände der gesetzlichen Krankenkassen und des Verbands der privaten Krankenversicherung e.V. zur versorgungsnahen Forschung „Chronische Krankheiten und Patientenorientierung". Sie ist dem Modul 2 „Rehabilitative Versorgung der Deutschen Rentenversicherung" zugeordnet und wird durch das BMBF und die Deutsche Rentenversicherung Bund finanziert (Fördermittel: 311.484, 00 Euro).

16. Die Aufwandsentschädigung für die Begutachtung (http://www.ethik.med.uni-erlangen.de/kosten.htm) wird übernommen von (*bitte Ansprechpartner benennen*):

Prof. Dr. Klaus Pfeifer; Sekretariat: Frau Jutta Preischl, App.-Nr.: 09131/85-28790

III. Angaben zu den Versuchsteilnehmern

1. Anzahl (*bei vergleichenden Studien bitte Aufteilung auf Gruppen angeben*)

- insgesamt 588 Versuchsteilnehmer
- davon entfallen jeweils 294 Probanden auf Interventions- und Kontrollgruppe

Bei Nullhypothesen-basierten Studien:
Wurde eine formale Fallzahlschätzung vorgenommen?
☒ ja ☐ nein

2. Alter und Geschlecht (*bitte geben Sie das Alter der Versuchsteilnehmer sowie die als Ausschlusskriterien vorgesehenen Ober- und Untergrenzen an*)

Es wird ein für die stationäre Rehabilitation von chronischen Rückenschmerzen repräsentatives Verhältnis von teilnehmenden Männern und Frauen angestrebt. Das definierte Altersspektrum beläuft sich auf Personen zwischen 18 und 65 Jahren. Es werden alters- und geschlechtsspezifische Interventionseffekte geprüft.

3. Status: Handelt es sich bei den Versuchsteilnehmern um
   - ☐ gesunde Personen
   - ☐ schwangere oder stillende Frauen
   - ☐ Kinder oder Jugendliche
   - ☒ einschlägig Erkrankte (*bitte geben Sie die Krankheit und das Stadium an*)
   - ☐ Personen, die an anderen Krankheiten leiden? (Insbesondere: psychische Krankheiten, die Zweifel an der Geschäfts- oder Einsichtsfähigkeit begründen)

Rehabilitanden mit chronischen und chronisch rezidivierenden unspezifischen Rückenschmerzen mit Reha-Hauptdiagnose (ICD-10: M54.4 – M54.9 sowie M51.2 – M51.9 und M53.8 - M53.9). Das Chronifizierungsstadium wird im Rahmen der Studie bestimmt; es werden Moderatoranalysen hinsichtlich der Wirksamkeit der Intervention vorgenommen.

4. Welche sonstigen **Einschlusskriterien** (z.B. erlaubte Begleitmedikation) sind vorgesehen?

Es sind keine weiteren Einschlusskriterien vorgesehen.

5. Welche sonstigen **Ausschlusskriterien** (z.B. fortgeschrittene Nieren- oder Leberinsuffizienz, verbotene Begleitmedikation etc.) sind vorgesehen?

- eindeutige zugrunde liegende spezifische Ursache für den Rückenschmerz (z.B. radikuläre Symptomatik, Myelopathie etc.)
- bereits erfolgte Operationen an der Wirbelsäule
- entzündliche Veränderung der Wirbelsäule
- deutlich eingeschränkter Gesundheitszustand (Begleiterkrankungen)
- Lebensalter unter 18 bzw. über 65 Jahre
- mangelnde Deutschkenntnisse
- schwerwiegende Beeinträchtigung des Seh- bzw. Hörvermögens (nicht korrigiert)
- schwerwiegende psychiatrische Zusatzdiagnosen
- Rentenantrag, Antragstellung nach § 51 SGB V

6. Sollen auch Personen teilnehmen, die auf gerichtliche oder behördliche Anordnung in einer Anstalt verwahrt werden?

☐ ja ☒ nein

7. Sollen auch Personen teilnehmen, die sich schon für andere Forschungsvorhaben zur Verfügung gestellt haben?

☐ ja ☒ nein

wie lange muss die letzte Teilnahme zurückliegen?

8. Bei Studien an Minderjährigen ***(nicht zutreffend)***

a. Warum kann die Studie nicht an Erwachsenen (voll Geschäftsfähigen) durchgeführt werden?

b. Sind Aufklärung und Einwilligung der (des) gesetzlichen Vertreter(s) gewährleistet?

☐ ja ☐ nein, weil

c. Sind zusätzliche Aufklärung und Einwilligung der minderjährigen (nicht voll geschäftsfähigen) Versuchsteilnehmer gewährleistet, die selbst in der Lage sind, Wesen, Bedeutung und Tragweite des Versuchs einzusehen und ihren Willen danach zu bestimmen?

☐ ja ☐ nein

9. Probandenversicherung

Wird zugunsten der Versuchsteilnehmer eine Versicherung abgeschlossen?

☐ ja (*bitte Police beifügen, aus der die Versicherungsgesellschaft und die Höhe der Versicherungsleistung hervorgeht)*

☒ nein

10. Schweigepflicht/Datenschutz

Werden die ärztlichen Schweigepflicht- und die Datenschutzbestimmungen beachtet?

Ja. Die Bestimmungen des Datenschutzgesetzes werden eingehalten. Die Probanden werden über die Weitergabe von ärztlichen Angaben informiert und müssen mit der Datenweitergabe einverstanden sein; diesbezügliche Entbindung von der Schweigepflicht (s. Patienteninformation).

11. Entgelt für Probanden

Soll den Versuchsteilnehmern ein Entgelt (Aufwandsentschädigung o.ä.) gezahlt werden?

☐ ja, in Höhe von EUR ☒ nein

11. Wie sollen die Versuchsteilnehmer über Wesen, Bedeutung und Tragweite der Studie **aufgeklärt** werden?

Die Patienten werden schriftlich (s. Patienteninformation) und mündlich durch den Rehabilitationsarzt aufgeklärt. Es besteht die Möglichkeit zu Rückfragen an den zuständigen Rehabilitationsarzt oder die wissenschaftliche Studienleitung.

12. Wie sollen die Versuchsteilnehmer ihre **Einwilligung** in die Teilnahme an der Studie erklären?

Die schriftliche Einwilligungserklärung ist Voraussetzung für die Studienteilnahme (s. Einwilligungserklärung).

Ich weiß, dass auch bei einer positiven Beurteilung des Vorhabens durch die Ethik-Kommission der Medizinischen Fakultät der FAU Erlangen-Nürnberg die ärztliche und juristische Verantwortung für die Durchführung des Projektes uneingeschränkt bei der Leiterin/dem Leiter verbleibt.

Erlangen/Nürnberg
Datum 18.4.2008

Unterschrift des/der Antragstellers/in

Prof. Dr. Klaus Pfeifer
(Name in Druckbuchstaben)

Unterschrift der/des Leiterin/Leiters der Einrichtung, in der das Vorhaben durchgeführt werden soll.
Mit der Durchführung des Forschungsvorhabens einverstanden:

Datum 18.4.2008

Unterschrift des/der Leiters/Leiterin der Einrichtung

Prof. Dr. Klaus Pfeifer
(Name in Druckbuchstaben)

EK_Oct06

Anlage zum Antrag

Literatur

AG Rehabilitation [Böhle, E., Bork, H,. Brüggemann, S., Greitemann, B., Hildebrandt, J., Hofmann, J., Kladny, B., Pfeifer, K] im Experten-Panel der Bertelsmann-Stiftung (2006). Qualitätsmanagement im Versorgungsprozess von Patienten mit Rückenschmerzen. „Best-Practice" - Empfehlungen zu Zielsetzungen, Inhalten und Methoden der Rehabilitation von Patienten mit chronifizierenden oder chronischen Rückenschmerzen. Projektbereicht bei der Bertelsmann-Stiftung.

Bandemer-Greulich, B., Bosse, B., Fikentscher, E., Konzag, T. A., Bahrke, U. (2008). Wirksamkeit psychologischer Interventionen auf die Schmerzverarbeitung innerhalb einer orthopädischen Rehabilitation von chronischen Rückenschmerzen. Psychther Psych Med, 58, 32-37.

Bosse, E., Fikentscher, E., Bandemer-Greulich, U., Müller, K., Bahrke, U., (2007). "Multimorbide" Rückenschmerzpatienten - Charakterisierung und Erfolg innerhalb der stationären orthopädischen Rehabilitation. Phys Med Rehab Kuror, 17, 197-202.

Greitemann et al. (2006). Integriertes Orthopädisch-Psychosomatisches Konzept zur medizinischen Rehabilitation von Patienten mit chronischen Schmerzen des Bewegungsapparates – Langfristige Effekte und Nachhaltigkeit eines multimodalen Programms zur Aktivierung und beruflichen Umorientierung. Z Orthop, 144, 255–266.

Gülich, M., Engel, E.-M., Rose, S., Klosterhuis, H., & Jäckel, W.H. (2003). Leitlinienentwicklung in der Rehabilitation von Rückenschmerzen – Phase 2: Ergebnisse einer KTL-Analyse. Die Rehabilitation 42, 109-117.

Guzmán et al. (2004). Multidisciplinary bio-psycho-social rehabilitation for chronic back-pain (Cochrane Review). In: The Cochrane Library, Issue 3. Chichester, UK: John Wiley & Sons, Ltd.

Hayden et al. (2005). Systematic Review: Strategies for Using Exercise Therapy To Improve Outcomes in Chronic Low Back Pain. Annual Intern Medicine, 142, 776–785.

Hoffman, B.M., Papas, R.K., Chatkoff, D.K. & Kerns, R.D. (2007). Meta-analysis of psychological interventions for chronic low back pain. Health Psychol. 26 (1), 1-9.

Hüppe, A. & Raspe, H. (2003). Die Wirksamkeit stationärer medizinischer Rehabilitation in Deutschland bei chronischen Rückenschmerzen: eine systematische Literaturübersicht 1980-2001. Rehabilitation 42, 143-154

Hüppe, A. & Raspe, H. (2005). Zur Wirksamkeit stationärer medizinischer Rehabilitation in Deutschland bei chronischen Rückenschmerzen: Aktualisierung und methodenkritische Diskussion einer Literaturübersicht. Rehabilitation 44, 24-33.

Kleist, B. et al. (2001). Work Hardening bei chronisch unspezifischen Rückenschmerzen in der stationären medizinischen Rehabilitation. Praxis Klinische Verhaltensmedizin und Rehabilitation, 14 (54), 145-150

Mehnert, A., Büttner, S., Sauer, C., Willmann, U., Bernhardt, R., Höcker, A., Jacobi, C., Herbold, D. & Koch, U. (2007). Wirksamkeit eines integrierten verhaltensmedizinischen orthopädischen Rehabilitationskonzepts hinsichtlich psychosozialer Erfolgsparameter – eine multizentrische Evaluationsstudie. In: Deutsche Rentenversicherung Bund (Hrsg.). Gesund älter werden – mit Prävention und Rehabilitation. DRV-Schriften: Bd. 72, S. 379-381.

Ostelo R.W.J.G., van Tulder, M.W., Vlaeyen, J.W.S., Linton, S.J., Morley, S.J. & Assendelft, W.J.J. (2005). Behavioural treatment for chronic low back pain. The Cochrane Database of Systematic Reviews, Issue1.

Pfingsten, M. & Hildebrandt, J. (2001). Die Behandlung chronischer Rückenschmerzen durch ein intensives Aktivierungskonzept - eine Bilanz von 10 Jahren. AINS 36: 580-589.

Schonstein et al. (2003. Work conditioning, work hardening and functional restoration for workers with back and neck pain (Cochrane Review) In: The Cochrane Library, Issue 3, Oxford: Update Software.

Schöps et al. (2000). Das Münchner Rücken-Intensiv-Programm (MÜRIP). Phys Med Rehab Kuror 10, 120-126.

Schwarz, S., Mangels, M., Holme, M. & Rief, W. (2007). Langzeitevaluation eines verhaltensmedizinischen Ansatzes in der orthopädischen Rehabilitation – eine randomisierte, kontrollierte Studie. In: Deutsche Rentenversicherung Bund (Hrsg.). Gesund älter werden – mit Prävention und Rehabilitation. DRV-Schriften: Bd. 72, S. 381-384.

van Tulder et al. (2003). Exercise therapy for low back pain (Cochrane Review) In: The Cochrane Library, Issue 3, 2003. Oxford: Update Software.

Vickers, A.J. (2005). Analysis of variance is easily misapplied in the analysis of randomized trials: a critique and discussion of alternative statistical approaches. Psychosomatic Medicine, 67, 652-655.

Worringen, U., Reinecke, A. & Mühlig, S. (2006). Das Gesundheitstrainingsprogramm der Deutschen Rentenversicherung Bund. RV aktuell, 53, 497-503.

FRIEDRICH-ALEXANDER
UNIVERSITÄT
ERLANGEN-NÜRNBERG

PHILOSOPHISCHE FAKULTÄT
UND FACHBEREICH THEOLOGIE

Institut für Sportwissenschaft und
Sport, AB Bewegung und Gesundheit
Prof. Dr. phil. Klaus Pfeifer

Julius-Maximilians-
UNIVERSITÄT
WÜRZBURG

Abteilung für Medizinische Psychologie und
Psychotherapie, Medizinische Soziologie und
Rehabilitationswissenschaften
Universität Würzburg
Prof. Dr. med. Dr. phil. Hermann Faller

Klinikeinrichtung

Ärztliche Leitung

14.04.2008

Patienteninformation

zum Forschungsvorhaben „Integrative Patientenschulung zur Optimierung der stationären Rehabilitation bei chronischem Rückenschmerz“

Liebe Patientin, lieber Patient,

wir freuen uns, Sie in der Rehabilitationseinrichtung in XXXX begrüßen zu können. Sie werden hier alle notwendigen und geeigneten Untersuchungen und Behandlungen, die zur wesentlichen Besserung oder Wiederherstellung Ihrer Gesundheit und Leistungsfähigkeit beitragen, erhalten.

Wir sind ständig bemüht, unser Behandlungsangebot den neuesten Erkenntnissen anzupassen und auch einen eigenen Beitrag hierzu zu leisten. Deshalb unterstützen wir das Forschungsvorhaben „Integrative Patientenschulung zur Optimierung der stationären Rehabilitation bei chronischem Rückenschmerz“, das von dem Institut für Sportwissenschaft und Sport der Universität Erlangen-Nürnberg und dem Arbeitsbereich Rehabilitationswissenschaften der Universität Würzburg an unserer Rehabilitationsklinik durchführt wird.

Unsere Ärzte und weitere Mitarbeiter unserer Rehabilitationseinrichtung sind aktiv an der Sammlung der Daten beteiligt und bilden die Verbindungsstelle zu den beiden Forschungsinstituten. Wir möchten Sie bitten, uns und damit das Forschungsvorhaben zu unterstützen. Ihre Teilnahme ist natürlich freiwillig (siehe Hinweise zur Freiwilligkeit).

Bevor Sie jedoch Ihre Einwilligung erteilen, erläutern wir Ihnen zunächst den geplanten Ablauf.

Darstellung des Forschungsvorhabens

Im Vorfeld der Behandlung wird geprüft, ob Sie für die Teilnahme an dem Forschungsprojekt in Frage kommen. Sollten Sie sich darüber hinaus für eine Teilnahme entscheiden, werden Daten folgendermaßen erhoben:

Grundlage bilden die für diese Rehabilitation bekannten und die im Verlaufe Ihres Aufenthaltes gewonnenen medizinischen Daten. Davon sind auch Angaben betroffen, die unserer Rehabilitationseinrichtung von den Kostenträgern oder anderen Sozialleistungsträgern zur Durchführung der Rehabilitation übermittelt werden. Diese Daten werden von unserem Klinikpersonal der Krankenblattakte entnommen. Des Weiteren werden auch gegebenenfalls Daten zum genauen Ablauf Ihrer Behandlung aus Ihrem Therapieplan benötigt. Ohne diese Daten ist das gesamte Forschungsvorhaben nicht sinnvoll durchzuführen. Wir bitten Sie daher, in die Nutzung dieser Daten einzuwilligen (siehe Einwilligungserklärung).

Mit der Studie wollen wir die Wirksamkeit eines nach den neuesten medizinischen Standards entwickelten Therapieprogramms bei chronischem Rückenschmerz überprüfen. Das neu entwickelte Programm zeichnet sich unter anderem dadurch aus, dass Sie gemeinsam mit anderen Patienten in einheitlichen Gruppen betreut werden. Die verschiedenen Behandlungselemente (z. B. medizinische Behandlung, Bewegungstherapie, Einübung von Strategien der Schmerzbewältigung und von Entspannungsverfahren) sowie das Vorgehen des Rehabilitationsteams sind dadurch besonders eng auf einander abgestimmt. Im Vordergrund steht dabei die Nutzung von Therapieeinheiten mit spezifischen und aufeinander abgestimmten Inhalten, Methoden, Medien und Patientenmaterialien für die Behandlung von chronischen Rückenschmerzen.

Die Studie findet in zwei Phasen statt. Falls Sie an der Studie teilnehmen möchten, werden Sie je nach Studienphase entweder dem klinikeigenen oder dem neu entwickelten Behandlungsprogramm zu gewiesen. Im Falle, dass Sie sich gegen die Teilnahme an diesem Forschungsprojekt entscheiden, werden Sie das Ihrem Krankheitsbild entsprechende, reguläre Behandlungsprogramm der Klinik erhalten. Dabei bekommen Sie Behandlungen aus den Behandlungsbereichen (Bewegungs-, Physio-, und physikalische Therapie) in etwa gleichem Umfang wie in dem neu entwickelten Behandlungsprogramm. Beide Behandlungsprogramme – das klinikeigene und das neu entwickelte – sind grundsätzlich dazu geeignet, Ihren Gesundheitszustand zu verbessern.

Wenn Sie mit der Teilnahme an unserem Forschungsvorhaben einverstanden sind, möchten wir Sie bitten, zu Rehabilitationsbeginn und –ende einen Fragebogen auszufüllen. Das Ausfüllen eines Fragebogens wird voraussichtlich rund 45 Minuten Ihrer Zeit in Anspruch nehmen.

Nach einem Zeitraum von einem Jahr möchten wir Sie zu der dann bestehenden Situation noch einmal befragen. Dazu werden Sie von unserer Klinik per Post einen Fragebogen erhalten, mit der Bitte, diesen auszufüllen und in dem beigefügten Freiumschlag an das Forschungsinstitut zurück zu senden. Die Zuordnung zu den

schon vorhandenen Daten erfolgt über die oben auf dem Fragebogen eingedruckte Forschungsnummer (siehe Hinweise zum Datenschutz).

Bevor Sie sich für eine Teilnahme entscheiden, geben wir in den folgenden Abschnitten Erläuterungen zu den Verantwortlichen, zu den Datenflüssen und zum Datenschutz.

Hinweise zum Datenschutz

Datenschutzrechtliche Bestimmungen sind immer dann zu beachten, wenn Einzelangaben (Daten) einer Person zugeordnet werden können. Für die wissenschaftliche Auswertung spielt es jedoch keine Rolle, von welcher Person die Daten stammen und wo diese z. B. wohnt. Wie es die Datenschutzgesetze fordern, werden die für die Auswertung vorgesehenen Forschungsdaten streng getrennt von den personenbezogenen Daten aufbewahrt.

Sobald Sie Ihre Teilnahme an dem Forschungsprojekt zusagen und die Einwilligung unterschrieben haben, trägt ein mit der Durchführung des Forschungsvorhabens betrauter Mitarbeiter Ihren Namen und Ihre Anschrift in eine fortlaufend nummerierte Liste ein. Die Nummer (Forschungsnummer) vor dem Namen ist das Kennzeichen, unter dem ab jetzt alle Forschungsdaten über Sie zusammengetragen werden. In den gesammelten Daten befinden sich keine Angaben, die direkt einen Bezug zu Ihrer Person herstellen lassen, z. B. Ihr Name.

Die Zuordnungsliste verbleibt in der Klinik und ist nur dem zuständigen Klinikpersonal zugänglich. Sie wird weder dem Forschungsinstitut noch anderen Stellen, z. B. der Deutschen Rentenversicherung bekannt gegeben. Sie dient ausschließlich dem Ziel, den Kontakt mit Ihnen herzustellen und die über Sie gewonnenen Daten unter der Forschungsnummer zu sammeln. Am Ende der Datenerhebung, also nach etwa zwei Jahren, werden wir die personenbezogenen Daten (Zuordnungsliste und die Einwilligungserklärungen) vernichten. So lässt sich auch keine Verbindung von den Forschungsnummern zu den Teilnehmern mehr herstellen.

An das Forschungsinstitut werden nur Daten zur statistischen Auswertung weiter gegeben, die keinen Personenbezug haben, also anonymisierte Daten. Bei eventuellen Veröffentlichungen durch das Forschungsinstitut kann kein Bezug zu Ihrer Person hergestellt werden.

Hinweise zur Freiwilligkeit

Die Teilnahme an dem Projekt ist freiwillig. **Sie können sich darauf verlassen, dass Ihre Daten nur verwendet werden, wenn Sie die Einwilligung unterschrieben haben.** Weder aus der Teilnahme noch aus einer Nichtteilnahme erwachsen Ihnen Nachteile. Wie auch Ihre Entscheidung ausfallen mag, sie hat auf die Durchführung der Rehabilitation keinen Einfluss.

Wenn Sie zu einem späteren Zeitpunkt aus dem Forschungsvorhaben ausscheiden möchten, ist das jederzeit, also auch bei bereits erteilter Einwilligung, ohne Angabe von Gründen möglich. Wir werden dann Ihren Namen in der oben beschriebenen Liste unkenntlich machen und Sie nicht mehr anschreiben. Wenn Sie es ausdrücklich

wünschen, werden wir auch Ihre für die Forschung gesammelten Daten löschen und schriftliche Aufzeichnungen (z. B. Fragebogen) vernichten, soweit diese noch existieren.

Wir bitten um Ihre Teilnahme!

Wenn Sie das Vorangegangene gelesen haben, Ihnen der Inhalt klar ist und Sie an dem Forschungsvorhaben teilnehmen möchten, bitten wir Sie, die beigefügte Einwilligungserklärung zu unterschreiben und im Sektretariat abzugeben bzw. in den vorgehenen Antwortkasten zu werfen.

Das hier vorliegende Informationsschreiben können Sie in jedem Fall behalten. Sie erhalten zusätzlich eine Kopie der unterschriebenen Einverständniserklärung.

Falls Fragen offen geblieben sind, stehen Ihnen die unten genannten Mitarbeiter der Rehabilitationsrichtung oder XXX und XXX aus den Arbeitsgruppen der Universitäten Erlangen-Nürnberg und Würzburg gern zur Verfügung.

Ansprechpartner des Forschungsteams

Arbeitsbereich Bewegung und Gesundheit an der Universität Erlangen-Nürnberg

xxxxxx Prof. Dr. Klaus Pfeifer

Arbeitsbereich Rehabilitationswissenschaften an der Universität Würzburg

xxxxxx Prof. Dr. Dr. Hermann Faller

Ansprechpartner in der Rehabilitationseinrichtung
xxxxxx

Prof. Dr. Klaus Pfeifer Prof. Dr. Dr. Hermann Faller Ärztliche Leitung

Friedrich-Alexander-Universität
Erlangen-Nürnberg

Institut für Sportwissenschaft und
Sport, AB Bewegung und Gesundheit
Prof. Dr. phil. Klaus Pfeifer

Julius-Maximilians-
UNIVERSITÄT
WÜRZBURG

Institut für Psychotherapie und Medizinische
Psychologie, AB Rehabilitationswissenschaften
Prof. Dr. med. Dr. phil. Hermann Faller

Klinikeinrichtung

Ärztliche Leitung

Einwilligungserklärung

Name: ______________________________ **Geburtsdatum:** ___________________

Ich bin über Inhalt und Zweck der Studie ***„Integrative Patientenschulung zur Optimierung der stationären Rehabilitation bei chronischem Rückenschmerz“***, die in Zusammenarbeit mit der Rehabilitationseinrichtung XXXX und den Universitäten Erlangen-Nürnberg und Würzburg durchgeführt wird, informiert worden. Zu diesem Zweck wurde mir ein Informationsschreiben ausgehändigt.

Mir wurde versichert, dass keine personenbezogenen Angaben (Name, Geburtsdatum, Adresse) oder sonstige Angaben, die Rückschlüsse auf meine Person zulassen, an Dritte weitergegeben werden und dass im Zusammenhang mit dieser Untersuchung erhobenen Daten gelöscht werden, sobald sie für die weitere wissenschaftliche Auswertung nicht mehr erforderlich sind.

Ich möchte das Forschungsvorhaben durch meine Beteiligung unterstützen und willige ein, Fragebogen auszufüllen, die mir ausgehändigt oder zugeschickt werden. Ich bin damit einverstanden, dass Klinikmitarbeiter behandlungsbezogene und medizinische Daten, ggf. auch von der Deutschen Rentenversicherung Bund mitgeteilte Angaben, aus der Klinikakte bzw. vorhandenen Therapieplänen entnehmen und den Forschungsunterlagen in der zugesicherten anonymen Form zuführen und an das wissenschaftliche Forschungsinstitut weiterleiten. Ich entbinde die Klinikmitarbeiter insoweit von ihrer Schweigepflicht.

Unter der in der Patienteninformation genannten Voraussetzung erkläre ich mein Einverständnis für die Teilnahme an dem Forschungsvorhaben.

Ort, Datum: _______________________

Unterschrift des Patienten / der Patientin: ______________________________

<u>Keine Teilnahme</u> an der Studie

Wir bitten Sie aus organisatorischen Gründen, uns ebenfalls mitzuteilen, wenn Sie an dem Forschungsvorhaben nicht teilnehmen möchten**. Bitte geben Sie dieses Blatt in jedem Fall im Schwesternzimmer ab.**
Des Weiteren ist es für uns von großem Interesse, die Gründe für Ihre Nichtteilnahme zu erfahren. Alle Angaben sind absolut freiwillig, würden uns aber die Planung weiterer Studien erleichtern.

Bitte kreuzen Sie im Folgenden diejenigen Gründe für Ihre Nichtteilnahme an, die auf Sie zu treffen (Mehrfachantworten sind möglich). Alternativ können Sie uns auch gern Ihre persönliche(n) Begründung(en) hinterlassen.

☐ Ich möchte generell keine Forschungsprojekte unterstützen.

☐ Das Ausfüllen von Fragebögen ist mir zu aufwendig.

☐ Ich bin mir unsicher, weil ich nicht genug Informationen habe.

☐ Ich möchte an keinem neuen Programm teilnehmen.

Sonstige Gründe:

___

___

___
