# Supplementary material for: Long-Term Effects of Interprofessional Biopsychosocial Rehabilitation for Adults with Chronic Non-Specific Low Back Pain: A Multicentre, Quasi-Experimental Study
Source: PLoS One. 2015 Mar 13;10(3):e0118609. doi: 10.1371/journal.pone.0118609 (PMC4359119; doi:10.1371/journal.pone.0118609)
Supplement: S1 Intervention — (PDF) [file pone.0118609.s004.pdf]

S1 Intervention description. Control group.

The three participating rehabilitation centres represent the traditional inpatient multidisciplinary orthopedic rehabilitation in Germany (usual care). They were similar regarding spatial and personnelwise requirements, internal organizational procedures and offered interventions. Nevertheless, due to the fact that in the rehabilitation centers no standardised rehabilitation concept for the treatment of chronic low back pain was available, some amount of variation in the treatment duration of single interventions within the three weeks of rehabilitation was reasonable. To control for large variations between the three rehabilitation centres, the duration of single interventions, and the proportion of patients who received single interventions (see section below), as well as the total duration of treatment (see table 1), and the total length of treatment (see table 1) was controlled during the preparation phase. The existing variations between the three rehabilitation centers were in acceptable ranges. Before the start of the control phase, all participating rehabilitation centres declared in a written contract, that no changes of the standard rehabilitation program would be undertaken during the control phase.

Duration of therapy and length of treatment

Table 1: Duration of therapy and length of treatment

	**centre 1**	**centre 2**	**centre 3**
duration of therapy in hours	50,5	44,2	47,5
length in days	22,6	23,7	23,8

Duration of single interventions

The following informations describe in more detail the single interventions of the standard inpatient rehabilitation in the three participating rehabilitation centers.

<u>Presentations with health informations</u>

The major themes of visual presentations with health informations were specific causes of low back pain such as fracture or herniated discs, red flags, and diagnostic procedures as well as treatment options. Further presentations included information about stress and stress management, health effects of exercise on the organism, as well as information about training principles. The extent of presentations with health

informations varied between 9.2h to 13.2h during the three week rehabilitation between the rehabilitation centres. The proportion of patients, who received presentations with health information, was 100%. Presentations were led by physicians, psychologists or physical therapists. Existing questions of the participants were answered during the presentation session. There were only few educational materials provided to the participants.

Exercise therapy and Back School

The major goal of exercise therapy was to improve physical fitness and to reduce physical deconditioning. Exercise therapy included back school with a biomedical approach. Back school in all three participating rehabilitation centers were held by the physical therapists and contained informations about spine anatomy, pathology, and optimal posture during sitting, standing, laying down, standing up, walking, bending, lifting or bearing, lumbar flexion, extension, and ergonomic counseling, and exercises aimed at strengthening the back muscles, stretching and relaxation exercises, muscular stabilization as well as advice on physical activity. The extent of back school between the three rehabilitation centers varied from 8 sessions each 30 minutes duration, over 1 session with 30 minutes duration plus 3 sessions each 45 minutes, to 10 sessions each 30 minutes duration. Questions from the participants were answered during the sessions, but there were only few interactive educational procedures used.

Other interventions of exercise therapy were also provided by physical therapists and included strengthening exercises such as functional gymnastics, weight-lifting training, medical training therapy, endurance exercises such as walking or nordic walking, ergometer cycling and aqua-jogging.

The total extent of exercise therapy varied between 11.5h to 25.3h. The proportion of patients, who received exercise therapy and back school, was 100%. The intensity of physical training was chosen according to physiologic training principles. There were only few materials provided to the participants (e.g. training schedule).

Physical treatments

Physical treatments included massage, superficial heat or cold, transcutaneous electrical nerve stimulation or traction. The major goals were to relieve pain and to improve relaxation. The total extent of physical treatments varied between 5.8h to 10.9h. The proportion of patients, who received physical treatments, was 83%-100%.

Psychological Interventions

Psychological interventions included pain coping groups, stress management such as progressive muscle relaxation, and if necessary individual counselling. The major goal was to improve pain coping. The major themes were:

- introduction of the multimodal approach,
- differences between acute versus chronic pain,
- pain perception, pain management, attention control,
- pain and thoughts, introduction of the gate-control theory, pain memory,
- reduction of maladaptive pain behavior,
- pain and distress, and
- Pleasure as pain management strategy.

The total extent of psychological interventions varied between 4.3h to 9.9h. The proportion of patients, who received psychological interventions, was 45% – 87%.
