# Supplementary material for: Long-Term Effects of Interprofessional Biopsychosocial Rehabilitation for Adults with Chronic Non-Specific Low Back Pain: A Multicentre, Quasi-Experimental Study
Source: PLoS One. 2015 Mar 13;10(3):e0118609. doi: 10.1371/journal.pone.0118609 (PMC4359119; doi:10.1371/journal.pone.0118609)
Supplement: S2 Intervention — (PDF) [file pone.0118609.s005.pdf]

S2 Intervention description. Intervention group.

Key characteristics of PASTOR

PASTOR is characterized by the following key features:

- close orientation to evidence-based best practice recommendations for objectives, contents and methods in the rehabilitation of CLBP for the German rehabilitation setting [1]
- explicit consideration of cognitive-behavioural mechanisms for the development of CLBP [2,3,4], of theories of health behaviour change [5,6,7] and of clinical evidence for active treatments in the management of CLBP [8, 9]
- intensified biopsychosocial [10], and interprofessional approach [11,12] with coordinated objectives, contents and methods
- a standardised comprehensive manualisation of modules with
  - specific subordinate goals and related contents,
  - description of the interactive education format and didactic approaches,
  - detailed description of interprofessional cross references to components of the other modules
  - specifically prepared educational material (short overviews, components, flipcharts, cards) on the influence of known risk factors for the development of CLBP (e.g. fear avoidance beliefs, catastrophizing, distress) for the members of the rehabilitation team as well as for the participants

- use of comprehensible educational material
- interprofessional reinforcement of positive messages
- a shared use of all contents, media and materials by all health professions, especially physicians, sport therapists, physiotherapists and psychologists, during the rehabilitation process
- fixed groups and therapist consistency and
- intensive interprofessional biopsychosocial training for all health professions before the start of the program

In sum, the interprofessional and interdisciplinary characteristics of PASTOR [12] range from an integrative combination of profession related modules within a comprehensive and consistent treatment approach, a collaborative teamwork base in profession related modules, a comprehensible manualisation of the entire treatment program with an explicit description of cross references between all treatment modules, a shared use of methods, media, and materials by all health professions, repetition of positive key messages by all health professions, and an interactive education approach to the interprofessional training of all health professionals carrying out PASTOR.

Distribution of sessions of each module

PASTOR consisted of the six interprofessional therapy modules:

- Education about low back pain (ELBP)
- Behavioural exercise therapy 1 (BET 1)
- Behavioural exercise therapy 2 (BET 2)
- Coping with pain (CWP)
- Relaxation (R)
- Workplace related information (WRI)

During the three week intervention phase, each rehabilitation centre provided these modules over the course of a twelve day rehabilitation program. Table 1 shows the distribution of the sessions for each module (this is identical to the PASTOR overview in S3 Intervention description. PASTOR therapy plan).

Table 1. Distribution of sessions of each module.

	days of therapy													
			1	2	3	4	5	6	7	8	9	10	11	12
interventions	admission	diagnostics	ELBP	ELBP	ELBP				ELBP				ELBP	
			BET1	BET1	BET1	BET1	BET1	BET1	BET1	BET1	BET1	BET1	BET1	BET1
				BET2	BET2	BET2	BET2	BET2	BET2	BET2	BET2	BET2	BET2	BET2
			CWP	CWP	CWP		CWP			CWP			CWP	
				R	R	R	R	R	R	R	R	R	R	R
								WRI			WRI			Dis-charge
						IT								

Modules: ELBP=Education about low back pain; BET 1/2=Behavioural exercise therapy; CWP=Coping with pain; R=Relaxation; AI= Workplace related information; IT=interdisciplinary team meeting

Interprofessional cross references

Single sessions within each module are linked to each other modules with explicit interprofessional cross-references. For example Figure 1 shows an interprofessional cross reference in the first day of PASTOR between the modules ELBP and BET1. During the first session of the ELBP module, a physician provides information about prevalence, course and risk factors for the development of low back pain using interactive short presentations and appealing media. These contents are repeated and consolidated by an exercise therapist in the following session of the module BET 1 using moderated group discussions, flipcharts, exercises and take-home cards for patients. These cards include important information, which were provided during the first session of ELBP and the first session of BET. More of these interprofessional cross-references between the sessions of each module are available in the entire rehabilitation program PASTOR.

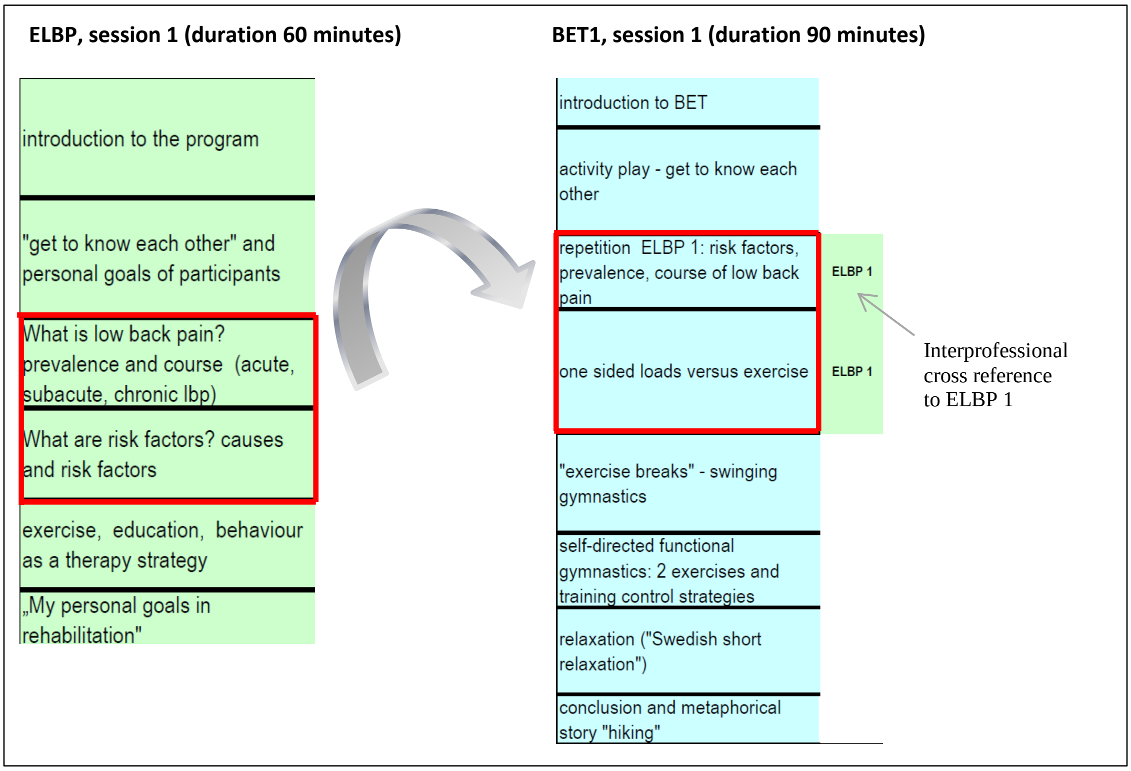

Figure 1. Example for an interprofessional cross reference between the first sessions of the modules ELBP and BET 1.

Trainer manual

A trainer manual is available in German, which describes the rehabilitation program PASTOR in great detail (http://www.forschung-patientenorientierung.de/files/pastor_etm_2012_1.pdf). It includes specific components, short overviews, and exercise collections (see table 2).

All profession related modules include components, which are provided in the single sessions of each module. Every **component** contains a detailed and specific description of each objective and related sub-goals, appropriate content, the use of behavioural techniques and interactive education format, duration, the use of media and combination with other components (see figure 3). These are complemented by **short overviews** in A3 format, which represent briefly the relevant components of a current session. These short overviews have an identical structure and provide information about the current session of each module, the involved profession, the components, the required therapist media, and patient materials (see figure 2).

Table 2. Overview about the composition of the trainer manual.

Content of the trainer manual	Number
Short overviews of ELBP	5
Short overviews of BET 1	12
Short overviews of CWP	6
Short overviews of WRI	2
Components of ELBP	19
Components of BET 1	86
Components of CWP	33
Components of WRI	9
Module BET 2	1
Module R	1
Exercise collection “functional gymnastics”	1
Exercise collection “relaxation”	1
Exercise collection “qi gong”	1
Cards for patients	75
Media for BET-therapists (flip chart)	6
Media for BET-therapists (DVD)	1

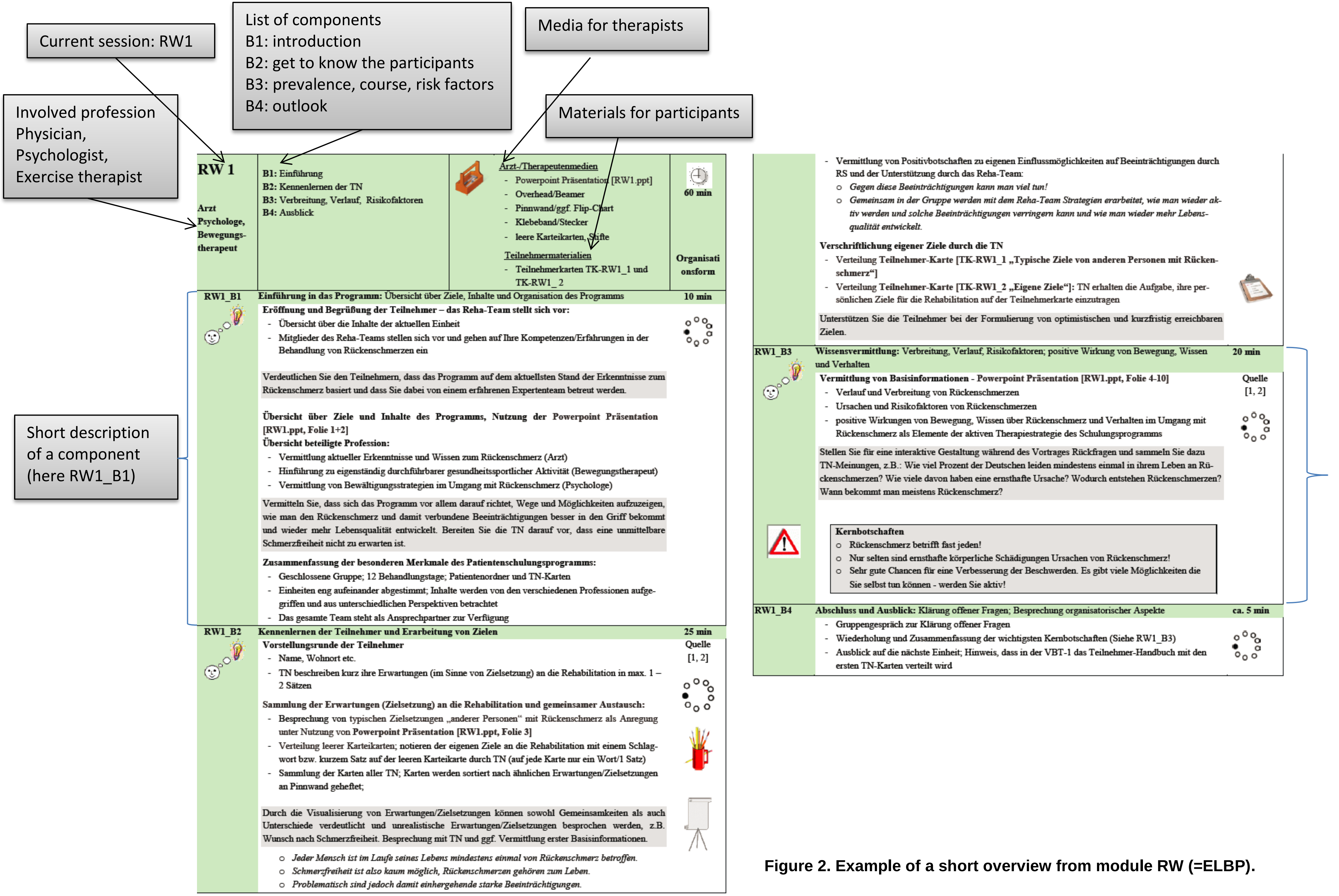

RW 1<br>Arzt<br>Psychologe,<br>Bewegungs-therapeut	B1: Einführung<br>B2: Kennenlernen der TN<br>B3: Verbreitung, Verlauf, Risikofaktoren<br>B4: Ausblick	Arzt-/Therapeutenmedien<br>- Powerpoint Präsentation [RW1.ppt]<br>- Overhead/Beamer<br>- Pinnwand/ggf. Flip-Chart<br>- Klebeband/Stecker<br>- leere Karteikarten, Stifte<br>Teilnehmermaterialien<br>- Teilnehmerkarten TK-RW1_1 und TK-RW1_2	60 min<br>Organisationsform
RW1_B1	**Einführung in das Programm:** Übersicht über Ziele, Inhalte und Organisation des Programms		10 min

Eröffnung und Begrüßung der Teilnehmer – das Reha-Team stellt sich vor:
- Übersicht über die Inhalte der aktuellen Einheit
- Mitglieder des Reha-Teams stellen sich vor und gehen auf Ihre Kompetenzen/Erfahrungen in der Behandlung von Rückenschmerzen ein

Verdeutlichen Sie den Teilnehmern, dass das Programm auf dem aktuellsten Stand der Erkenntnisse zum Rückenschmerz basiert und dass Sie dabei von einem erfahrenen Expertenteam betreut werden.

Übersicht über Ziele und Inhalte des Programms, Nutzung der Powerpoint Präsentation [RW1.ppt, Folie 1+2]
Übersicht beteiligte Profession:
- Vermittlung aktueller Erkenntnisse und Wissen zum Rückenschmerz (Arzt)
- Hinführung zu eigenständig durchführbarer gesundheitssportlicher Aktivität (Bewegungstherapeut)
- Vermittlung von Bewältigungsstrategien im Umgang mit Rückenschmerz (Psychologe)

Vermitteln Sie, dass sich das Programm vor allem darauf richtet, Wege und Möglichkeiten aufzuzeigen, wie man den Rückenschmerz und damit verbundene Beeinträchtigungen besser in den Griff bekommt und wieder mehr Lebensqualität entwickelt. Bereiten Sie die TN darauf vor, dass eine unmittelbare Schmerzfreiheit nicht zu erwarten ist.

Zusammenfassung der besonderen Merkmale des Patientenschulungsprogramms:
- Geschlossene Gruppe; 12 Behandlungstage; Patientenordner und TN-Karten
- Einheiten eng aufeinander abgestimmt; Inhalte werden von den verschiedenen Professionen aufgegriffen und aus unterschiedlichen Perspektiven betrachtet
- Das gesamte Team steht als Ansprechpartner zur Verfügung

RW1_B2	**Kennenlernen der Teilnehmer und Erarbeitung von Zielen**	25 min

Vorstellungsrunde der Teilnehmer — Quelle [1, 2]
- Name, Wohnort etc.
- TN beschreiben kurz ihre Erwartungen (im Sinne von Zielsetzung) an die Rehabilitation in max. 1 – 2 Sätzen

Sammlung der Erwartungen (Zielsetzung) an die Rehabilitation und gemeinsamer Austausch:
- Besprechung von typischen Zielsetzungen „anderer Personen“ mit Rückenschmerz als Anregung unter Nutzung von **Powerpoint Präsentation [RW1.ppt, Folie 3]**
- Verteilung leerer Karteikarten; notieren der eigenen Ziele an die Rehabilitation mit einem Schlagwort bzw. kurzem Satz auf der leeren Karteikarte durch TN (auf jede Karte nur ein Wort/1 Satz)
- Sammlung der Karten aller TN; Karten werden sortiert nach ähnlichen Erwartungen/Zielsetzungen an Pinnwand geheftet;

Durch die Visualisierung von Erwartungen/Zielsetzungen können sowohl Gemeinsamkeiten als auch Unterschiede verdeutlicht und unrealistische Erwartungen/Zielsetzungen besprochen werden, z.B. Wunsch nach Schmerzfreiheit. Besprechung mit TN und ggf. Vermittlung erster Basisinformationen.

- *Jeder Mensch ist im Laufe seines Lebens mindestens einmal von Rückenschmerz betroffen.*
- *Schmerzfreiheit ist also kaum möglich, Rückenschmerzen gehören zum Leben.*
- *Problematisch sind jedoch damit einhergehende starke Beeinträchtigungen.*

- Vermittlung von Positivbotschaften zu eigenen Einflussmöglichkeiten auf Beeinträchtigungen durch RS und der Unterstützung durch das Reha-Team:
  - *Gegen diese Beeinträchtigungen kann man viel tun!*
  - *Gemeinsam in der Gruppe werden mit dem Reha-Team Strategien erarbeitet, wie man wieder aktiv werden und solche Beeinträchtigungen verringern kann und wie man wieder mehr Lebensqualität entwickelt.*

Verschriftlichung eigener Ziele durch die TN
- Verteilung Teilnehmer-Karte [TK-RW1_1 „Typische Ziele von anderen Personen mit Rückenschmerz“]
- Verteilung Teilnehmer-Karte [TK-RW1_2 „Eigene Ziele“]: TN erhalten die Aufgabe, ihre persönlichen Ziele für die Rehabilitation auf der Teilnehmerkarte einzutragen

Unterstützen Sie die Teilnehmer bei der Formulierung von optimistischen und kurzfristig erreichbaren Zielen.

RW1_B3	**Wissensvermittlung:** Verbreitung, Verlauf, Risikofaktoren; positive Wirkung von Bewegung, Wissen und Verhalten	20 min

Vermittlung von Basisinformationen - Powerpoint Präsentation [RW1.ppt, Folie 4-10] — Quelle [1, 2]
- Verlauf und Verbreitung von Rückenschmerzen
- Ursachen und Risikofaktoren von Rückenschmerzen
- positive Wirkungen von Bewegung, Wissen über Rückenschmerz und Verhalten im Umgang mit Rückenschmerz als Elemente der aktiven Therapiestrategie des Schulungsprogramms

Stellen Sie für eine interaktive Gestaltung während des Vortrages Rückfragen und sammeln Sie dazu TN-Meinungen, z.B.: Wie viel Prozent der Deutschen leiden mindestens einmal in ihrem Leben an Rückenschmerzen? Wie viele davon haben eine ernsthafte Ursache? Wodurch entstehen Rückenschmerzen? Wann bekommt man meistens Rückenschmerz?

Kernbotschaften
- Rückenschmerz betrifft fast jeden!
- Nur selten sind ernsthafte körperliche Schädigungen Ursachen von Rückenschmerz!
- Sehr gute Chancen für eine Verbesserung der Beschwerden. Es gibt viele Möglichkeiten die Sie selbst tun können - werden Sie aktiv!

RW1_B4	**Abschluss und Ausblick:** Klärung offener Fragen; Besprechung organisatorischer Aspekte	ca. 5 min

- Gruppengespräch zur Klärung offener Fragen
- Wiederholung und Zusammenfassung der wichtigsten Kernbotschaften (Siehe RW1_B3)
- Ausblick auf die nächste Einheit; Hinweis, dass in der VBT-1 das Teilnehmer-Handbuch mit den ersten TN-Karten verteilt wird

Figure 2. Example of a short overview from module RW (=ELBP).

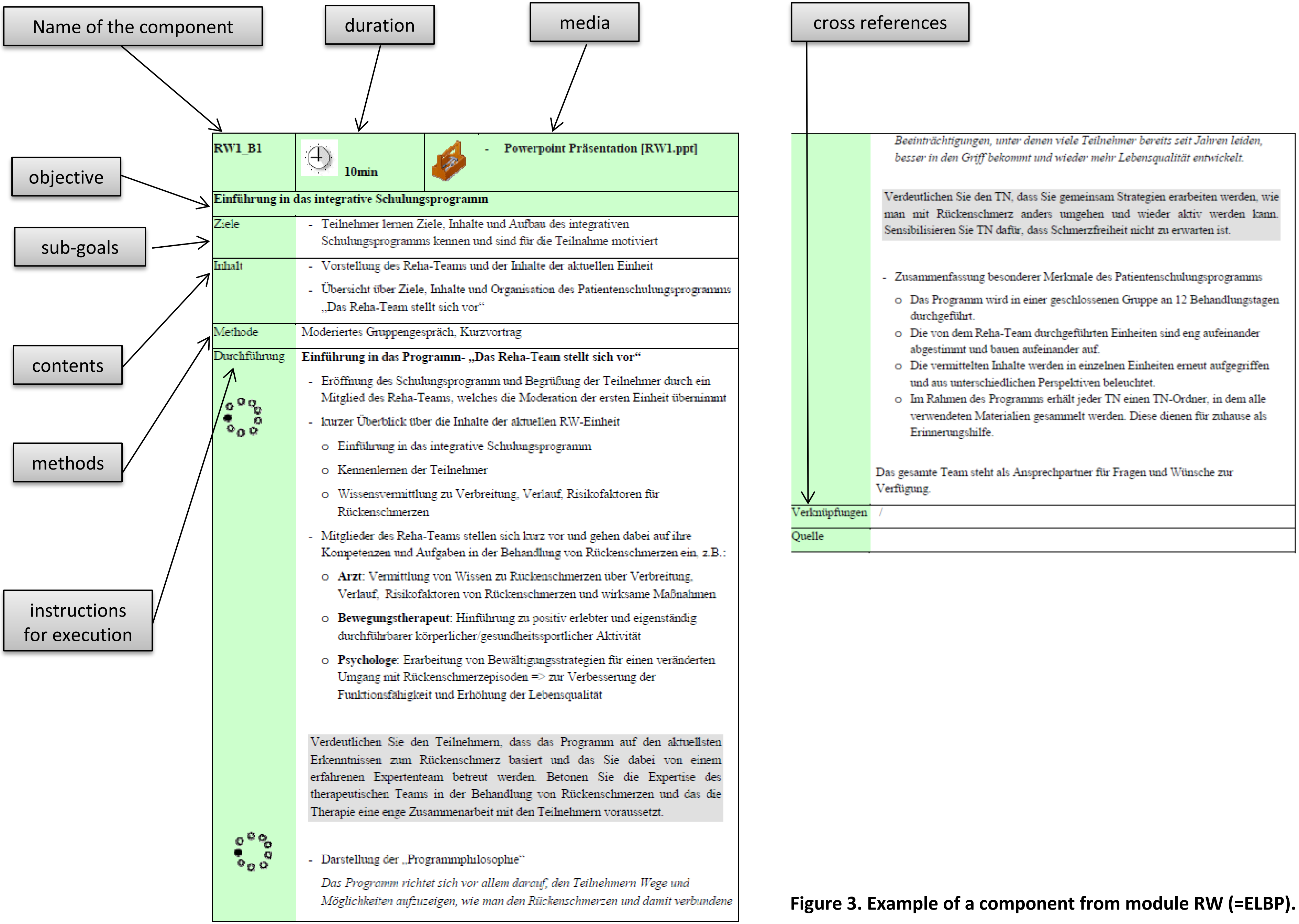

RW1_B1	10min	- **Powerpoint Präsentation [RW1.ppt]**
**Einführung in das integrative Schulungsprogramm**		
Ziele	- Teilnehmer lernen Ziele, Inhalte und Aufbau des integrativen Schulungsprogramms kennen und sind für die Teilnahme motiviert	
Inhalt	- Vorstellung des Reha-Teams und der Inhalte der aktuellen Einheit<br>- Übersicht über Ziele, Inhalte und Organisation des Patientenschulungsprogramms „Das Reha-Team stellt sich vor“	
Methode	Moderiertes Gruppengespräch, Kurzvortrag	
Durchführung	**Einführung in das Programm- „Das Reha-Team stellt sich vor“**<br>- Eröffnung des Schulungsprogramm und Begrüßung der Teilnehmer durch ein Mitglied des Reha-Teams, welches die Moderation der ersten Einheit übernimmt<br>- kurzer Überblick über die Inhalte der aktuellen RW-Einheit<br>o Einführung in das integrative Schulungsprogramm<br>o Kennenlernen der Teilnehmer<br>o Wissensvermittlung zu Verbreitung, Verlauf, Risikofaktoren für Rückenschmerzen<br>- Mitglieder des Reha-Teams stellen sich kurz vor und gehen dabei auf ihre Kompetenzen und Aufgaben in der Behandlung von Rückenschmerzen ein, z.B.:<br>o **Arzt**: Vermittlung von Wissen zu Rückenschmerzen über Verbreitung, Verlauf, Risikofaktoren von Rückenschmerzen und wirksame Maßnahmen<br>o **Bewegungstherapeut**: Hinführung zu positiv erlebter und eigenständig durchführbarer körperlicher/gesundheitssportlicher Aktivität<br>o **Psychologe**: Erarbeitung von Bewältigungsstrategien für einen veränderten Umgang mit Rückenschmerzepisoden => zur Verbesserung der Funktionsfähigkeit und Erhöhung der Lebensqualität<br><br>Verdeutlichen Sie den Teilnehmern, dass das Programm auf den aktuellsten Erkenntnissen zum Rückenschmerz basiert und das Sie dabei von einem erfahrenen Expertenteam betreut werden. Betonen Sie die Expertise des therapeutischen Teams in der Behandlung von Rückenschmerzen und das die Therapie eine enge Zusammenarbeit mit den Teilnehmern voraussetzt.<br><br>- Darstellung der „Programmphilosophie“<br>*Das Programm richtet sich vor allem darauf, den Teilnehmern Wege und Möglichkeiten aufzuzeigen, wie man den Rückenschmerzen und damit verbundene*	

	Beeinträchtigungen, unter denen viele Teilnehmer bereits seit Jahren leiden, besser in den Griff bekommt und wieder mehr Lebensqualität entwickelt.<br><br>Verdeutlichen Sie den TN, dass Sie gemeinsam Strategien erarbeiten werden, wie man mit Rückenschmerz anders umgehen und wieder aktiv werden kann. Sensibilisieren Sie TN dafür, dass Schmerzfreiheit nicht zu erwarten ist.<br><br>- Zusammenfassung besonderer Merkmale des Patientenschulungsprogramms<br>o Das Programm wird in einer geschlossenen Gruppe an 12 Behandlungstagen durchgeführt.<br>o Die von dem Reha-Team durchgeführten Einheiten sind eng aufeinander abgestimmt und bauen aufeinander auf.<br>o Die vermittelten Inhalte werden in einzelnen Einheiten erneut aufgegriffen und aus unterschiedlichen Perspektiven beleuchtet.<br>o Im Rahmen des Programms erhält jeder TN einen TN-Ordner, in dem alle verwendeten Materialien gesammelt werden. Diese dienen für zuhause als Erinnerungshilfe.<br><br>Das gesamte Team steht als Ansprechpartner für Fragen und Wünsche zur Verfügung.
Verknüpfungen	/
Quelle	

Figure 3. Example of a component from module RW (=ELBP).

Interprofessional education approach

The interactive education approach within the modules of ELBP, CWP, R, and WRI include short presentations, and moderated group discussions, working in small groups, films, short stories, as well as individual work. The interactive education approach within the modules of BET 1 and 2 include moderated group discussions with the use of flipcharts and take-home cards for patients for knowledge transfer, demonstration or verbal instructions by the exercise therapist, individual practice or group exercises, monitoring tasks and attention shift, connecting cognitive and motor learning processes, as well as perception of tension and relaxation of muscles. In every module participants have the opportunity to ask questions and describe their own experiences.

Therapist media and patient materials

Therapist media (e.g. flipcharts) and take-home cards for patients support education on various topics. They are designed with a patient oriented focus and include cues to action. All cards are gradually distributed in the modules ELBP (2 cards), BET1/ 2 (44 cards), CWP (22 cards/ work sheets), and WRI (7 cards) by the members of the rehabilitation team and are collected by the patients in a folder (see cards in figure 4 as example).

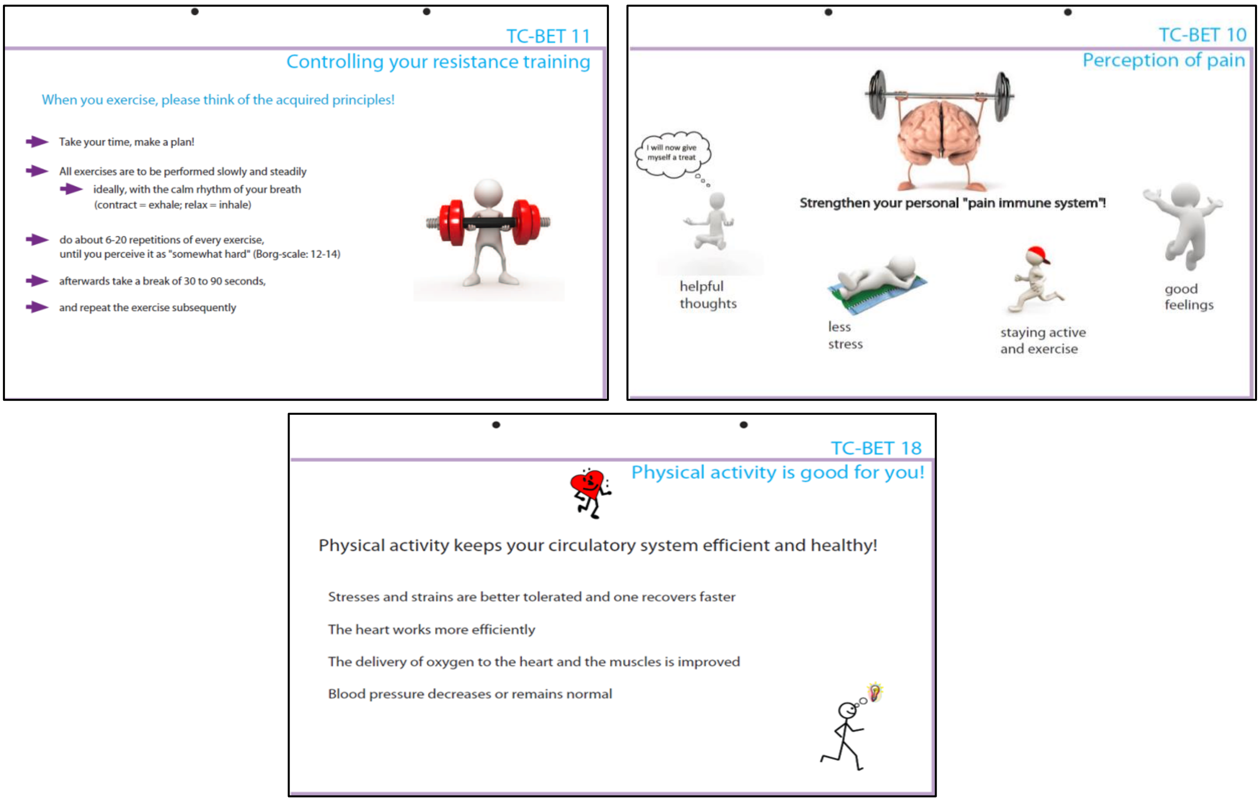

Figure 4. Examples for take-home cards from the BET module.

References

1. Hofmann J, Böhle E, Bork H, Brüggemann S, Greitemann B, Hildebrandt J, et al. Best-practice-recommendations for objectives, contents and methods in the outpatient and inpatient rehabilitation of chronic low back pain. Phys Med Rehabil Kurortmed. 2010;20: 32–39.
2. Hasenbring MI, Verbunt JA. Fear-avoidance and Endurance-related Responses to Pain: New Models of Behavior and Their Consequences for Clinical Practice. Clin J Pain. 2010;26: 747–753.
3. Leeuw M, Goossens MEJB, Linton SJ, Crombez G, Boersma K, Vlaeyen JWS. The fear-avoidance model of musculoskeletal pain: current state of scientific evidence. J Behav Med. 2007;30: 77–94.
4. Pincus T, Smeets RJEM, Simmonds MJ, Sullivan MJL. The fear avoidance model disentangled: improving the clinical utility of the fear avoidance model. Clin J Pain. 2010;26: 739–746.
5. Geidl W, Hofmann J, Göhner W, Sudeck G, Pfeifer K. Verhaltensbezogene Bewegungstherapie - Bindung an einen körperlich aktiven Lebensstil. Rehabilitation. 2012;51: 259–268.
6. Schwarzer R, Lippke S, Luszczynska A. Mechanisms of health behavior change in persons with chronic illness or disability: The Health Action Process Approach (HAPA). Rehabil Psychol. 2011;56: 161–170.
7. Biddle SJ, Fuchs R. Exercise psychology: A view from Europe. Psychol Sport Exerc. 2009;10: 410–419.
8. van Middelkoop M, Rubinstein SM, Verhagen AP, Ostelo RW, Koes BW, van Tulder MW. Exercise therapy for chronic nonspecific low-back pain. Best Pract Res Clin Rheumatol. 2010;24: 193–204.
9. Hayden JA, van Tulder MW, Tomlinson G. Systematic review: strategies for using exercise therapy to improve outcomes in chronic low back pain. Ann Intern Med. 2005;142: 776–785.
10. Waddell G. The biopsychosocial model. In: Waddell G, editor. The back pain revolution. Edinburgh: Churchill Livingstone; 2004. pp. 265–282.
11. Körner M. Interprofessional teamwork in medical rehabilitation: a comparison of multidisciplinary and interdisciplinary team approach. Clin Rehabil. 2010;24: 745–755.
12. World Health Organisation. Framework for Action on Interprofessional Education & Collaborative Practice. 2010. Available: http://www.who.int/hrh/resources/framework_action/en/. Accessed 4 April 2013.
