# Supplementary material for: Long-Term Effects of Interprofessional Biopsychosocial Rehabilitation for Adults with Chronic Non-Specific Low Back Pain: A Multicentre, Quasi-Experimental Study
Source: PLoS One. 2015 Mar 13;10(3):e0118609. doi: 10.1371/journal.pone.0118609 (PMC4359119; doi:10.1371/journal.pone.0118609)
Supplement: S3 Intervention — (PDF) [file pone.0118609.s006.pdf]

S3 Intervention description. PASTOR therapy plan.

Moduls/Days	1	2	3	4	5	6	7	8	9	10	11	12
Education about low back pain (ELBP) (sessions 1 to 5) (30-60 minutes)	t = 60´<br>introduction to the Program<br>"get to know each other" and personal goals of participants<br>What is low back pain? prevalence and course (acute, subacute, chronic lbp)<br>What are risc factors? causes and risk factors<br>exercise, education, behaviour as therapy strategy<br>„My personal goals in rehabilitation"	t = 45´<br>review<br>Why persists my back pain? lack of causes, possibilities and limitations of diagnostic procedures<br>factors of chronification<br>consequences of pain<br>pain memonry<br>(CWP 1)	t = 45´<br>review<br>dealing with low back pain: passive and active self-management strategies<br>the spine as facinating and strong system<br>relationsship between physical activity and functional ability<br>muscle soreness<br>(BETI_2)				t = 30´<br>review and take home messages<br>exchange of experiences about therapy<br>behavior during recurring pain episodes "flare ups"<br>information about pain killers; red flags<br>distribution of the back book (german version)<br>(CWP)				final discussion; review/ reflection; answering questions about the back book<br>final discussion about aftercare (BETI_12 CWP 6)<br>adoption; clarification of open questions	
behavioral exercie therapy (BET I) (sessions 1 to 12) 90 minutes	introduction BET<br>activity play - get to know each other<br>repetition ELBP 1: risk factors, prevalence, course of low back pain (ELBP 1)<br>one sides loads versus exercise (ELBP 1)<br>"exercise breaks" - swinging gymnastics<br>self-directed functional gymnastic: 2 exercises and training control strategies<br>relaxation ("Swedish short relaxation")<br>conclusion and metaphorical story "hiking"	activity play - get to know each other<br>repetition ELBP 2: consequen-ces of pain, positiv effects of physical activity/ exercises (ELBP 2)<br>"exercise breaks" - swinging gymnastics<br>introduction to walking and training control strategies (subjective perceived exertion)<br>self-directed functional gymnastic: further exercises and training control strategies<br>metaphorical story "mushroom picker"	activity play - get to know each other<br>repetition ELBP 3: the spine as facinating and strong system (ELBP 3)<br>"exercise breaks" - swinging gymnastics<br>introduction to walking and training control strategies (subjective perceived exertion & manual pulse measurement)<br>self-directed functional gymnastic: further exercises and training control strategies<br>relaxation	activity play - positive exercise experiences<br>effects of physical activity on the organism: „Exercise is good for your body" (ELBP 3)<br>active play ("ball transportation") (ELBP 3)<br>effects of physical activity on mood and well-being (ELBP 2)<br>functional gymnastic (*standard program*)	activity play - positive exercise experiences<br>physical activity/ exercise as pleasure; mood management (CWP 3)<br>functional gymnastic (*standard program*)<br>action planning - introduction	activity play - positive exercise experiences<br>repetition UMS 4 „coper, avoider, endurer" (CWP 4)<br>activity play - positive exercise experiences<br>education: muscularly stabilization of the spine: How does it work? (ELBP 3)<br>introduction of lumbar stabilization exercises: activation of deep and global trunk muscles<br>functional gymnastic (*standard program*)<br>action planning - reinforcement, homework "pedometer/ step counter"	activity play - positive exercise experiences<br>education: muscularly stabilization of the spine (ELBP 3)<br>training of stabilization exercises<br>variation of the body awareness exercise „human pendulum"<br>muscularly stabilization during activities of daily life (ELBP 3)<br>functional gymnastic (*standard program*)<br>relaxation	activity play - positive exercise experiences<br>repetition muscularly stabilization during activities of daily life<br>… during work related physical activities<br>… one sided loads and compensation strategies (ELBP 3)<br>repetition ELBP 4: behavior during recurring pain episodes "flare ups" (ELBP 4)<br>functional gymnastic (*standard program*)<br>relaxation	activity play - positive exercise experiences<br>repetition - self-directed exercises and BET II<br>physical activity in everyday life<br>alternative exercises "Qi Gong"<br>functional gymnastic (*standard program*)<br>relaxation	activity play - positive exercise experiences<br>action and coping planning for home – activity goals after rehabilitation, barriers and barrier management<br>alternative exercises "Qi Gong"<br>functional gymnastic (*standard program*)<br>relaxation	activity play - positive exercise experiences<br>action and coping planning for home – activity goals after rehabilitation, barriers and barrier management<br>alternative exercises "Qi Gong"<br>functional gymnastic (*standard program*)<br>relaxation	activity play - positive exercise experiences<br>alternative exercises "Qi Gong"<br>information about aftercare, support in contacting providers (ELBP 5, CWP 6)<br>final discussion and "take home messages"
BET II (sessions 1 to 11) 45- 60 minutes		introduction to self-directed aerobic exercises (walking, cycling swimming) (BET I)	introduction to self-directed weight-lifting training (BET I)	introduction to self-directed aerobic exercises (walking, cycling swimming) (BET I)	introduction to self-directed weight-lifting training (BET I)	self-directed health enhancing exercises (e.g. walking; muscle training) (BET I)	self-directed health enhancing exercises (e.g. walking; muscle training) (BET I)	self-directed health enhancing exercises (e.g. walking; muscle training) (BET I)	self-directed health enhancing exercises (e.g. walking; muscle training) (BET I)	self-directed health enhancing exercises (e.g. walking; muscle training) (BET I)	self-directed health enhancing exercises (e.g. walking; muscle training) (BET I)	self-directed health enhancing exercises (e.g. walking; muscle training) (BET I)
Coping with pain (CWP) (sessions 1 to 6) 60 minutes	introduction UMS (aims, content, group rules<br>pain perception, gate-control theory, movie clips, "pain defense system" (ELBP1)<br>"exercise breaks" (BETI)<br>short movie "Basketball", effects of distraction, coping with acute pain<br>summary, key messages	review ("pain defense system", pain memory, individual experiences) (ELBP1)<br>repetition „mushroom picker" BET I_ 2 (BETI)<br>thoughts avalanche, "pain-mood-circle" (ELBP2)<br>"exercise breaks" (BETI)<br>pain and cognitions - photo story "well-being circle"<br>summary, key messages	review (coping with acute pain)<br>effects of relaxation and physical activity, imaginary journey (BETI, BETTII) (ELBP2)<br>"exercise breaks" (BETI)<br>pleasure, well-being, recreation<br>summary, key messages		review (activation of "pain defense system", individual experiences)<br>fear-avoidance-beliefs, case example "Martina" (BET I)<br>"exercise breaks" (BET I)<br>pain persistence behaviour, case example "Thorsten" (BET I)<br>adaptive pain coping strategies when dealing with light or heavy pain<br>summary, key messages			review<br>stress reactions, conse-quences of pain, job dissatis-faction and low back pain (ELBP 2)<br>"exercise breaks" (BETI)<br>meaning of stress promoting beliefs/ attitudes<br>Stress management environment, appraisal, relaxation)<br>summary, key messages			review<br>repetition personal goals, progress in therapy, action- and coping planning for dealing with back pain (ELBP 1) (BETI)<br>"exercise breaks" (BETI)<br>dealing with recurring pain episodes "flare ups" (ELBP 4)<br>information about aftercare (ELBP 5)<br>final discussion	
Relaxation (R) (sessions 1 to 11) 30´- 45 minutes		introduction to relaxation: progressive muscle relaxation (CWP)	practice of relaxation: progressive muscle relaxation (CWP)	practice of relaxation: progressive muscle relaxation (CWP)	practice of relaxation: progressive muscle relaxation (CWP)	practice of relaxation: progressive muscle relaxation (CWP)	practice of relaxation: progressive muscle relaxation (CWP)	practice of relaxation: progressive muscle relaxation (CWP)	practice of relaxation: progressive muscle relaxation (CWP)	practice of relaxation: progressive muscle relaxation (CWP)	practice of relaxation: progressive muscle relaxation (CWP)	practice of relaxation: progressive muscle relaxation (CWP)
Work place related information (WRI) (sessions 1 and 2) 60 minutes				**interdisziplinary team meeting**		rehabilitation and retirement<br>definitions of social medicine<br>work place related risc factors, occupational diseases of the spine?<br>performance evaluation, case examples<br>final discussion and participants materials			review<br>Disabilities Act (Social Code IX), case examples<br>employment participation benefits and vocational rehabilitation<br>final discussion			

Legend

Colour of each cell represents each component (ELBP= green BET I = light blue, BET II = yellow, CWP = red, R = purple, WRI = beige)!

Coloured cells without short description (e.g. ELBP 1) = connection to other components
