# Supplementary material for: Long-Term Effects of Interprofessional Biopsychosocial Rehabilitation for Adults with Chronic Non-Specific Low Back Pain: A Multicentre, Quasi-Experimental Study
Source: PLoS One. 2015 Mar 13;10(3):e0118609. doi: 10.1371/journal.pone.0118609 (PMC4359119; doi:10.1371/journal.pone.0118609)
Supplement: S1 Measurement Instruments — (PDF) [file pone.0118609.s007.pdf]

S1 Measurement instruments.

Functional Ability

The FFbH-R [1] consists of twelve items with a three-stage answering scale (2=yes; 1=yes, but with difficulty; 0=no, or only with assistance). The summary score describes the low back pain associated functional ability in activities of daily living (e.g. “Can you wash and dry yourself from head to toe?”) in adults on a scale of 0% (minimum functional ability) to 100% (maximum functional ability).

Mental and physical health status

Mental and physical health status during the past four weeks was assessed by using the German version of the SF-12 Health Survey [2]. Based on twelve items the mental and physical health statuses were calculated as two dimensions of health-related quality of life. The scores were summarized, calibrated and transformed in a scale ranging from zero to 100. Higher scores represent higher quality of life.

Pain intensity

Pain intensity was measured by three numerical rating-scales taken from the German pain questionnaire [3] with an eleven-stage answering scale ranging from 0=“no impairment” to 10=“completely impaired” for the pain intensity categories “at the moment”, “on average” and “greatest” adapted for the time period of the last week. The average pain intensity was calculated as the mean value of the three scales.

Physical activity

Physical activity was assessed by self-report with the “Freiburg Questionnaire of physical activity” [4]. The questionnaire consists of eight items and allows patients to rate their physical activities levels in the dimensions of daily routine activities, leisure time activities, and sport activities. For all physical activities, participants were asked to indicate duration (minutes or hours) during the last week or month that they engaged in a physical activity (e.g. “Did you ride your bicycle on your way to your work or for shopping the last week?” [yes/no], “If so, how long have you cycled?” [minutes or hours]). For sport activities, participants were also asked to describe the type of the activities (e.g. “Did you perform sport activities last week?” [yes/no], “If so, what type of sport activities?” [type and minutes or hours/ week or month]). A

subscore was calculated for each dimension of basic activity, leisure time activity, as well as a total activity score for all activity reports (hours per week).

Pain Coping Strategies

The German Pain Management Questionnaire (“FESV”) is a measure of cognitive and behavioural pain coping strategies [5]. It contains a cognitive and a behavioural domain, and each domain has three dimensions. Every dimension is rated by four items. The cognitive domain comprises the dimensions action-oriented coping (e.g. “If I have pain, I have a plan how to handle that pain”), cognitive restructuring (e.g. “If I have pain, I will not give up”), and subjective coping competence (e.g. “If I have pain, I remind myself that I can deal with it much better than in the past”). The behavioural domain consists of the dimensions “mental distraction” (e.g. “If I have pain, I thumb through a magazine”), “counter activities” (e.g. “If I have pain, I distract myself with activities around my home or garden”), and “relaxation” (e.g. “If I have pain, I concentrate on calm and steady breathing”). Altogether there are 24 items and the responses recorded on a 6-point scale (ranging from 1=“complete disagreement” to 6=“complete agreement”). Responses are summed up in a total score, with a possible range from 4 to 24. Higher values represent a greater use of the respective strategy.

Fear-avoidance response and endurance response to pain

The Avoidance-Endurance Questionnaire (AEQ) [6] was used to assess two patterns of responses to pain: the fear-avoidance response and the endurance response. Nine sub-scales are available to measure affective (anxiety/depression; positive mood), cognitive (help-/hopelessness; catastrophizing; thought suppression) and behavioural (avoidance of social activities; avoidance of physical activities; pain persistence behaviour; humor/distraction) responses to pain.

The fear-avoidance response pattern is represented by five sub-scales with anxiety/depression (seven items), help-/hopelessness (nine items), catastrophizing (three items), avoidance of social activities (six items) and avoidance of physical activities (five items). The endurance response pattern consists of four sub-scales including positive mood (three items), thought suppression (four items), humor/distraction (five items) and pain persistence behaviour (four items). Respondents are asked to record their answers based on the past 14 days on a 7-

point scale (ranging from 0="never" to 6="every time"). Furthermore, behavioural responses to pain are recorded for mild pain, as well as for severe pain.

References

1. Kohlmann T, Raspe H. Der Funktionsfragebogen Hannover zur alltagsnahen Diagnostik der Funktionsbeeinträchtigung durch Rückenschmerzen (FFbH-R). Rehabilitation. 1996;34: I–VIII.
2. Bullinger M, Kirchberger I. Der SF-36 Fragebogen zum Gesundheitszustand: Handanweisung. 1st ed. Göttingen: Hogrefe; 1998.
3. Nagel B, Gerbershagen HU, Lindena G, Pfingsten M. Entwicklung und empirische Überprüfung des Deutschen Schmerzfragebogens der DGSS. Schmerz. 2002;16: 263–270.
4. Frey I, Berg A., Grathwohl D., Keul J. Freiburger Fragebogen zur körperlichen Aktivität – Entwicklung, Prüfung und Anwendung. Soz Präventivmed. 1999;44: 55–64.
5. Geissner E. Fragebogen zur Erfassung der Schmerzverarbeitung (FESV): Manual. 1st ed. Göttingen: Hogrefe; 2001.
6. Hasenbring MI, Hallner D, Rusu AC. Fear-avoidance- and endurance-related responses to pain: Development and validation of the Avoidance-Endurance Questionnaire (AEQ). Eur J Pain. 2009;13: 620–628.
