# Supplementary material for: Long-Term Effects of Interprofessional Biopsychosocial Rehabilitation for Adults with Chronic Non-Specific Low Back Pain: A Multicentre, Quasi-Experimental Study
Source: PLoS One. 2015 Mar 13;10(3):e0118609. doi: 10.1371/journal.pone.0118609 (PMC4359119; doi:10.1371/journal.pone.0118609)
Supplement: S1 Table — (PDF) [file pone.0118609.s008.pdf]

S1 Table. LMM results secondary analysis.

Table: LMM (secondary analyses) - secondary outcomes

Outcome	Control group mean change	Intervention group mean change	adjusted mean difference (95% CI)	*P*	d
**Physical health status (SF 12)**					
t1-t2	5.32	5.82	0.50 (-0.99 to 1.99)	0.509	0.029
t2-t3	-1.38	-1.68	-0.30 (-1.89 to 1.30)	0.714	-0.016
total	3.93	4.14	0.20 (-1.64 to 2.05)	0.828	0.009
**Mental health status (SF 12)**					
t1-t2	6.51	7.13	0.62 (-1.35 to 2.58)	0.537	0.027
t2-t3	-6.14	-5.36	0.78 (-1.36 to 2.91)	0.475	0.031
total	0.37	1.77	1.39 (-0.66 to 3.45)	0.184	0.058
**Low back pain intensity (NRS)**					
t1-t2	-1.48	-1.53	-0.05 (-0.34 to 0.25)	0.755	-0.013
t2-t3	0.47	0.20	-0.27 (-0.65 to 0.09)	0.148	-0.063
total	-1.01	-1.33	-0.32 (-0.71 to 0.07)	0.103	-0.071
**Sport activity (hours/week) (FfkA)**					
t1-t2	---	---	---	---	---
t1-t3/ total	0.15	0.77	0.63 (0.12 to 1.13)	0.015	0.106
**Total physical activity (hours/week) (FfkA)**					
t1-t2	---	---	---	---	---
t2-t3/total	1.13	1.81	0.68 (-1.18 to 2.54)	0.473	**0.031**
**Action-oriented coping (FESV)**					
t1-t2	1.56	3.92	2.36 (1.50 to 3.22)	**<0.001**	**0.232**
t2-t3	-0.65	-0.80	-0.15 (-0.96 to 0.66)	0.719	-0.016
total	0.91	3.12	2.21 (1.29 to 3.13)	**<0.001**	**0.203**
**Subjective coping competence (FESV)**					
t1-t2	0.78	2.38	1.60 (0.91 to 2.29)	**<0.001**	**0.197**
t2-t3	-0.21	-0.70	-0.49 (-1.22 to 0.24)	0.189	-0.057
total	0.57	1.68	1.11 (0.25 to 1.97)	**0.012**	**0.110**
**Cognitive restructuring (FESV)**					
t1-t2	0.97	3.43	2.47 (1.68 to 3.26)	**<0.001**	**0.265**
t2-t3	-0.17	-1.13	-0.96 (-1.79 to -0.13)	**0.024**	**-0.098**
total	0.80	2.30	1.51 (0.60 to 2.41)	**0.001**	**0.141**
**Counter activities (FESV)**					
t1-t2	0.82	3.04	2.21 (1.51 to 2.94)	**<0.001**	**0.263**
t2-t3	-0.92	-0.65	0.27 (-0.48 to 1.02)	0.473	0.031
total	-0.11	2.40	2.50 (1.63 to 3.37)	**<0.001**	**0.244**
**Mental distraction (FESV)**					
t1-t2	1.30	3.10	1.80 (1.0 to 2.61)	**<0.001**	**0.190**
t2-t3	-0.88	-0.64	0.23 (-0.59 to 1.04)	0.588	0.023
total	0.44	2.46	2.03 (1.12 to 3.0)	**<0.001**	**0.187**
**Relaxation (FESV)**					
t1-t2	2.15	4.23	2.09 (1.25 to 2.92)	**<0.001**	**0.213**
t2-t3	-0.36	-1.19	-0.83 (-1.67 to 0.02)	0.055	-0.083
total	1.79	3.05	1.26 (0.36 to 2.16)	**0.006**	**0.119**
**Help-/hopelessness (AEQ)**					
t1-t2	-0.29	-0.58	-0.29 (-0.45 to -0.13)	**<0.001**	**-0.158**
t2-t3	0.14	0.09	-0.05 (-0.24 to 0.14)	0.617	-0.022
total	-0.15	-0.49	-0.34 (-0.55 to -0.12)	**0.002**	**-0.133**
**Catastrophizing (AEQ)**					

t1-t2	-0.13	-0.24	-0.12 (-0.27 to 0.05)	0.131	-0.065
t2-t3	0.12	0.05	-0.07 (-0.25 to 0.11)	0.437	-0.034
total	-0.003	-0.19	-0.19 (-0.39 to 0.02)	0.072	-0.078
**Thought suppression (AEQ)**					
t1-t2	-0.06	-0.15	-0.09 (-0.31 to 0.14)	0.455	-0.032
t2-t3	-0.09	-0.08	0.01 (-0.25 to 0.26)	0.946	0.003
total	-0.15	-0.23	-0.08 (-0.36 to 0.20)	0.599	-0.023
**Anxiety/depression (AEQ)**					
t1-t2	-0.66	-0.91	-0.25 (-0.44 to -0.06)	**0.009**	**-0.114**
t2-t3	0.50	0.47	-0.03 (-0.24 to 0.18)	0.800	-0.011
total	-0.16	-0.44	-0.28 (-0.50 to -0.05)	**0.015**	**-0.105**
**Positive mood (AEQ)**					
t1-t2	0.59	0.75	0.16 (-0.04 to 0.36)	0.123	0.067
t2-t3	-0.42	-0.41	0.01 (-0.23 to 0.25)	0.919	0.004
total	0.17	0.34	0.17 (-0.09 to 0.43)	0.196	0.056
**Avoidance of physical activities when dealing with severe pain (AEQ)**					
t1-t2	-0.23	-0.55	-0.32 (-0.50 to -0.14)	**<0.001**	**-0.150**
t2-t3	-0.01	-0.04	-0.03 (-0.22 to 0.16)	0.730	-0.015
total	-0.23	-0.59	-0.36 (-0.57 to -0.15)	**0.001**	**-0.144**
**Avoidance of social activities when dealing with severe pain (AEQ)**					
t1-t2	-0.18	-0.53	-0.35 (-0.54 to -0.16)	**<0.001**	**-0.157**
t2-t3	-0.09	-0.17	-0.08 (-0.30 to 0.14)	0.459	-0.032
total	-0.27	-0.70	-0.43 (-0.69 to -0.18)	**<0.001**	**-0.147**
**Humor/distraction when dealing with severe pain (AEQ)**					
t1-t2	0.24	0.51	0.27 (0.09 to 0.46)	**0.004**	**0.125**
t2-t3	-0.03	0.01	0.04 (-0.17 to 0.24)	0.716	0.016
total	0.21	0.52	0.31 (0.10 to 0.53)	**0.005**	**0.122**
**Pain persistence behaviour when dealing with severe pain (AEQ)**					
t1-t2	0.02	0.11	0.09 (-0.06 to 0.24)	0.231	0.052
t2-t3	-0.05	-0.05	-0.01 (-0.19 to 0.18)	0.945	-0.003
total	-0.03	0.06	0.09 (-0.10 to 0.28)	0.375	0.039

LMM=linear mixed model; t1=baseline, t2=end of rehabilitation, t3=12-month follow-up; CI=confidence interval; *P*=significance value; bold=significant between-group difference (P<0.05); d=Effect size Cohen's d for the between group difference; SF 12=Short Form 12, NRS=Numeric Rating Scale, FfkA=Freiburg Questionnaire of Physical Activity, FESV=Pain Management Questionnaire, AEQ=Avoidance Endurance Questionaire
