# Supplementary material for: Long-Term Effects of Interprofessional Biopsychosocial Rehabilitation for Adults with Chronic Non-Specific Low Back Pain: A Multicentre, Quasi-Experimental Study
Source: PLoS One. 2015 Mar 13;10(3):e0118609. doi: 10.1371/journal.pone.0118609 (PMC4359119; doi:10.1371/journal.pone.0118609)
Supplement: S1 Ethical Approval — (PDF) [file pone.0118609.s009.pdf]

Medizinische Fakultät
Ethik-Kommission

Friedrich-Alexander-Universität
Erlangen-Nürnberg

Ethik-Kommission • Krankenhausstraße 12 • D-91054 Erlangen

Geschäftsstelle der Ethik-Kommission
Tel. (09131) 85-22270
Fax (09131) 85-26021
E-mail:ethik@zuv.uni-erlangen.de
http://www.ethik.med.uni-erlangen.de

Herrn
Prof. Dr. K. Pfeifer
Institut für Sportwissenschaft und Sport
Gebbertstr. 123 b
91058 Erlangen

Erlangen, 07.05.2008

Antrag an die Ethik-Kommission/Re.-No. 3807
Integrative Patientenschulung zur Optimierung der stationären Rehabilitation bei chronischem Rückenschmerz.
Patienteninformation und Einwilligungserklärung; Fragebogen –Keine Teilnahme an der Studie
(Eingang der vollständigen Unterlagen am 28.04.2008)

Sehr geehrter Herr Kollege Pfeifer,

die Ethik-Kommission der Medizinischen Fakultät hat in ihrer Sitzung vom 06.05.2008 über berufsethische und berufsrechtliche Aspekte Ihres oben bezeichneten Antrags beraten.

Die Studie wurde mit der folgenden Auflage zustimmend bewertet:

In der Patientenaufklärung muss erläutert werden, worin genau die Abweichungen von der Standardtherapie bestehen.

Auch bei einer positiven Beurteilung des Vorhabens durch die Ethik-Kommission der Medizinischen Fakultät der Friedrich-Alexander-Universität Erlangen-Nürnberg verbleibt die ärztliche und juristische Verantwortung für die Durchführung des Projekts uneingeschränkt bei Ihnen und Ihren Mitarbeitern/innen.

Sollten sich zu diesem Projekt ethisch relevante Nachträge ergeben, bitte ich Sie, diese der Ethik-Kommission unverzüglich zusammen mit einer Bewertung der Nutzen-Risiko-Relation bekannt zu geben. Änderungen in den Dokumenten sind zum Zweck einer beschleunigten Bearbeitung deutlich zu kennzeichnen. Die Ethik-Kommission erbittet einen Kurzbericht nach Abschluss der Studie.

Die Gültigkeit des Votums der Ethik-Kommission ist an die im Antrag angegebene Laufzeit der Studie gebunden.

Mit freundlichen kollegialen Grüßen

(Prof. Dr. med. P. Betz)
Stellv. Vorsitzender der Ethik-Kommission

Anlage:
Teilnehmerliste

An der Sitzung der Ethik-Kommission der Medizinischen Fakultät der Universität Erlangen-Nürnberg am 06.05.2008 haben teilgenommen:

Frau Prof. Dr. med. Kerstin Amann
Stellv. Frauenbeauftragte der Medizinischen Fakultät
Fachgebiet Pathologie
Institut für Pathologie

Herr Prof. Dr. med. Peter Betz
Stellv. Vorsitzender der Ethik-Kommission
Fachgebiet Rechtsmedizin
Direktor des Instituts für Rechtsmedizin

Herr Prof. Dr. med. M. Fromm
Fachgebiet Klinische Pharmakologie und Klinische Toxikologie
Direktor am Institut für Experimentelle und Klinische Pharmakologie und Toxikologie

Herr Prof. Dr. med. Dieter Harms
Fachgebiet Kinderheilkunde
ehem. Leitender Oberarzt an der Kinder- und Jugendklinik

Herr Dietmar Klieber
Vorsitzender Richter am Oberlandesgericht Nürnberg a.D.

Herr Prof. Dr. med. Torsten Kuwert
Fachgebiet Klinische Nuklearmedizin
Direktor der Nuklearmedizinischen Klinik

Herr Prof. Dr. phil. Günter R. Schmidt
Fachgebiet Praktische Theologie (Katechetik und Religionspädagogik)
ehem. Leiter am Institut für Praktische Theologie

Herr Prof. Dr. med. Dr. rer. nat. Helmut Schwilden
Fachgebiet Experimentelle Anästhesiologie
Leiter der Experimentellen Anästhesiologie, Anästhesiologische Klinik
